# Supplementary material for: Mechanistic model of MAPK signaling reveals how allostery and rewiring contribute to drug resistance
Source: Mol Syst Biol. 2023 Jan 26;19(2):e10988. doi: 10.15252/msb.202210988 (PMC9912026; doi:10.15252/msb.202210988)
Supplement: Supplementary file 1 — Expanded View Figures PDF [file MSB-19-e10988-s005.pdf]

Expanded View Figures

Figure EV1. Overview calibrated model simulation and experimental data.

Experiments were performed in A375 cell lines in 5% FBS medium following 24 h of drug adaptation (unless otherwise noted). EGF stimulation was at 100 ng/ml. Data are shown as point-ranges with average over technical replicates ($n = 2$) as point and estimated standard deviation (over all data points) as line. Data from different experiments (biological replicates) are shown separately. Median simulations are shown as thick lines and shading indicates 80% percentiles over 50 best parameter sets.

A Phospoproteomic training data (RAFi dose response). Experiments were performed after 24 h pretreatment with drug/DMSO without EGF stimulation.

B Proteomic training data (RAFi dose response). Experiments were performed after 24 h pretreatment with drug/DMSO without EGF stimulation.

C Transcriptomic training data. RAFi dose response (left three panels) and time course (right three panels). Dose response was performed after 24 h pretreatment with drug/DMSO without EGF stimulation. Time-course measurements were collected after 24 h pretreatment with 1 μM vemurafenib.

D Time resolved RAFi and MEKi drug response immunofluorescence data.

E Time course immunofluorescence data for different pretreatment times (for drug adaption). Pretreatment time indicates the time between drug treatment (1 μM vemurafenib) and EGF stimulation (100 ng/ml).

© 2023 The Authors

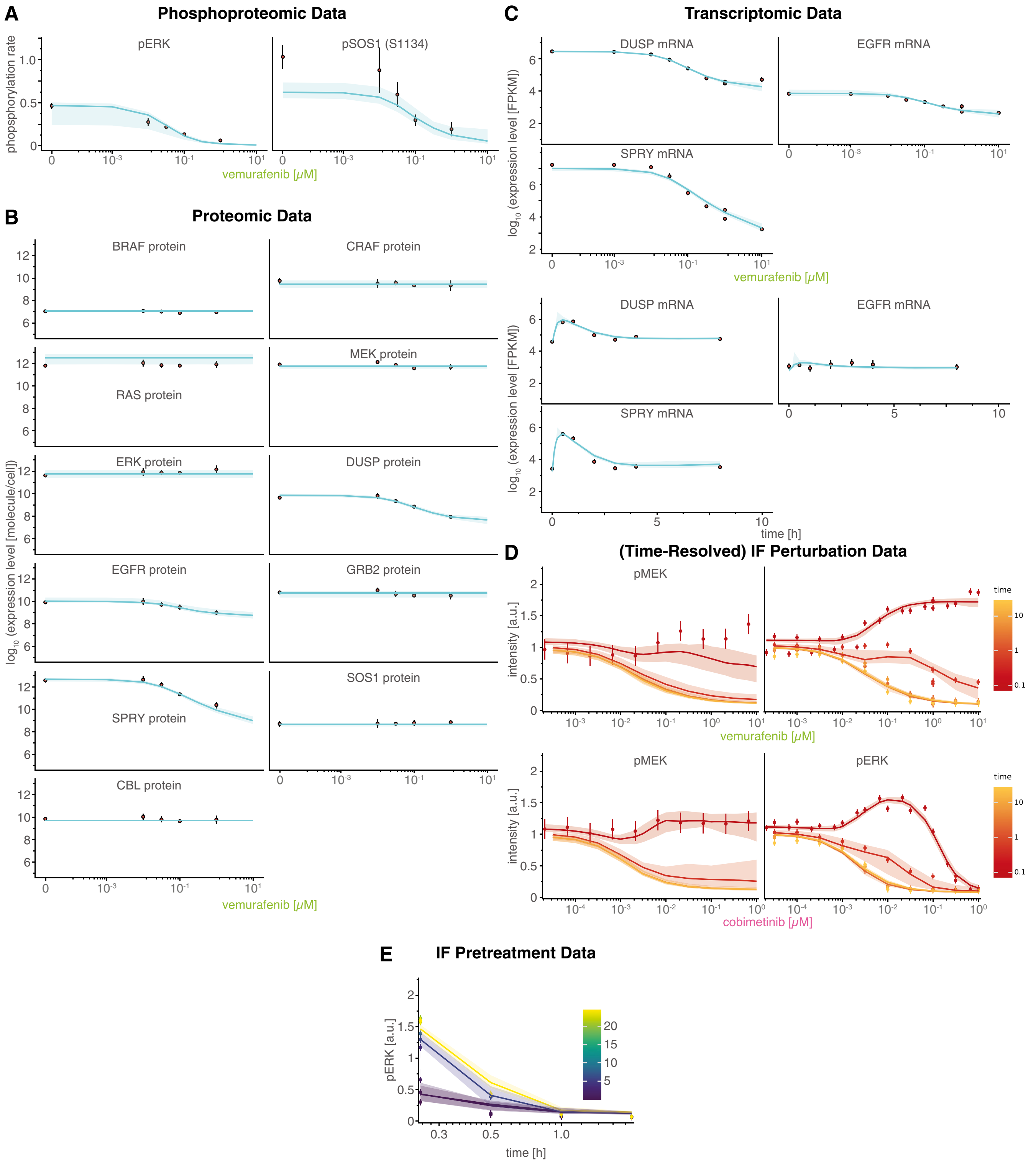

Figure EV1.

© 2023 The Authors

Figure EV2. Variability in parameter estimates.

A Boxplot of parameter estimates for best 50 parameter sets. Optimization boundary is indicated as dashed lines. Type of parameters are indicated by suffix: _0 (expression level), _dG (thermodynamic encoding of affinity, not on log scale), _ddG (thermodynamic encoding of allosteric interactions, not on log scale), _eq (baseline expression level), _gexpslope (RNA synthesis scaling factor), _kM (pERK concentration at which 50% activation is achieved), _kbase (baseline phosphorylation rate), _kcat (catalytic rate), _kcatr (normalized kcat), _kdeg (degradation rate), _kf (binding rate), _offset (background intensity), _scale (observable scaling). Central band shows media, box extends from lower to upper quartile values and whiskers show full range excluding outliers (points more than 1.5 interquartile ranges away from lower and upper quartiles).

B Correlation plots of parameter estimates. Only statistically significant ($P > 0.05$, Bonferroni-Holm corrected two-tailed t-test) correlations are shown. Coloring shows positive (red)/negative (blue) correlation.

© 2023 The Authors

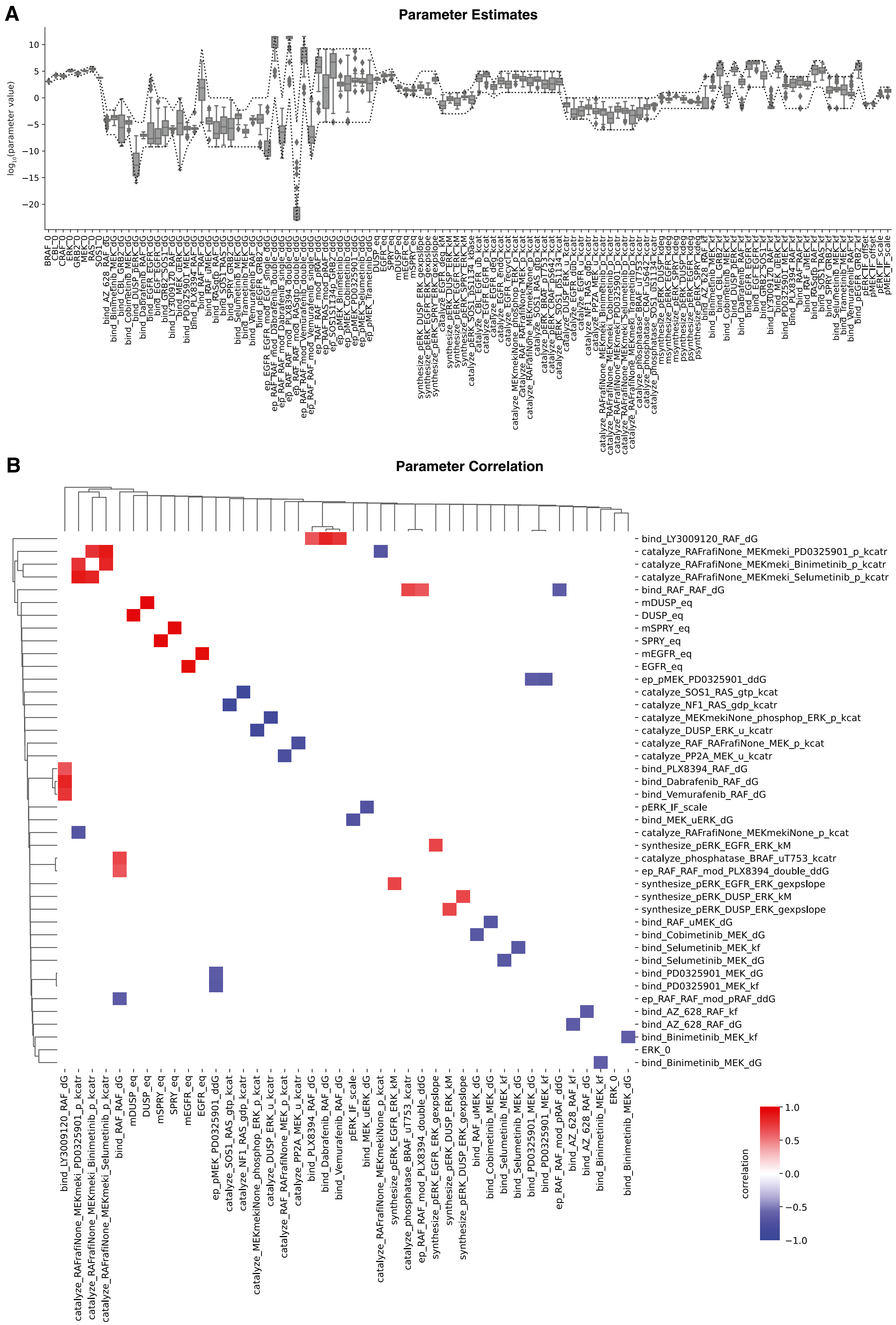

Figure EV2.

 © 2023 The Authors

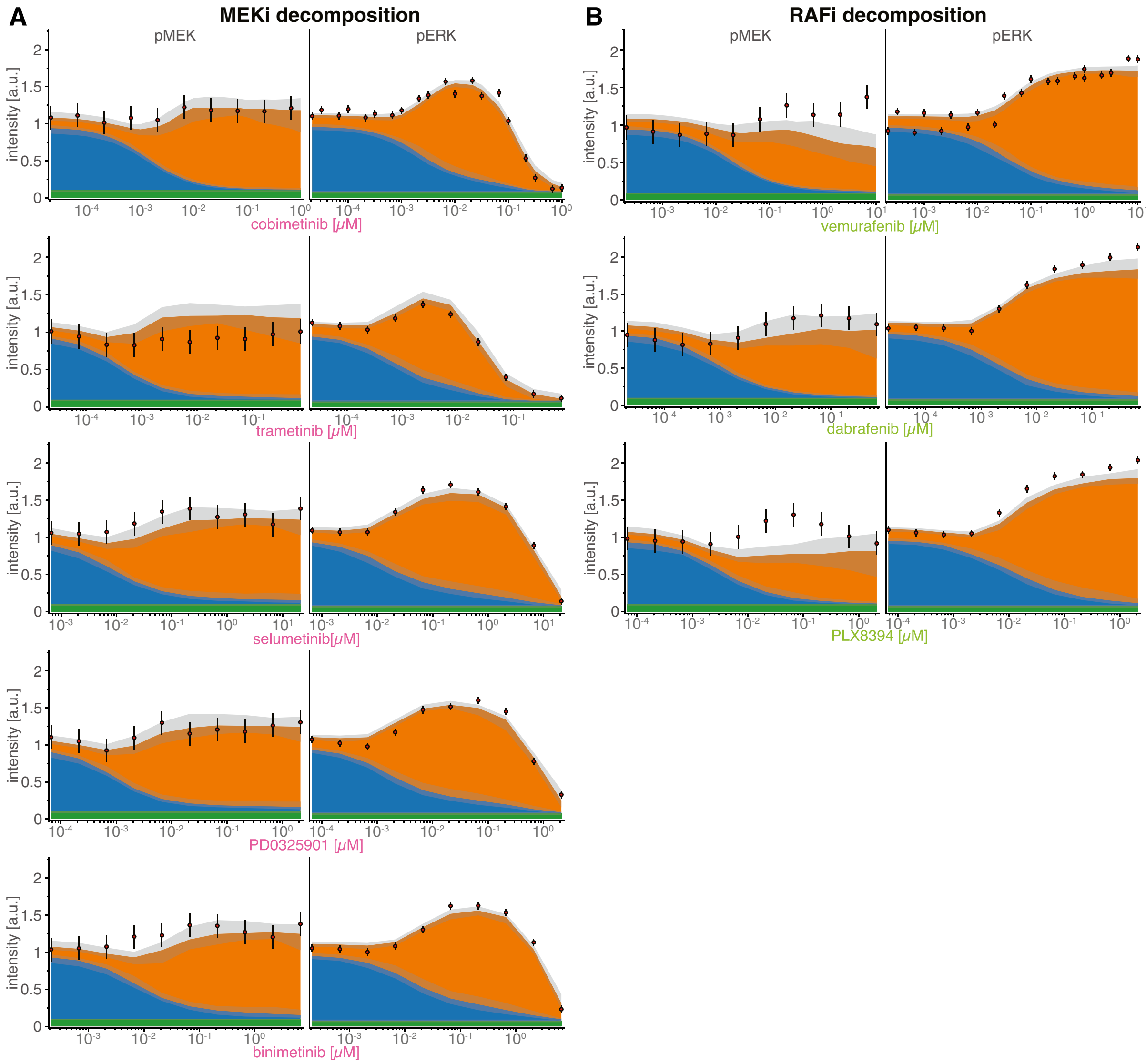

Figure EV3. Causal Decomposition of RAS and $BRAF^{V600E}$ channels (extended).

Experiments were performed in A375 cell lines in 5% FBS medium after 24 h of drug adaptation. EGF stimulation was at 100 ng/ml.

A, B Comparison of experimental data and decomposed model simulations for pERK (left panels) and pMEK (right panels) at 5 min after EGF stimulation for five different MEK inhibitors (A) and three different RAF inhibitors (B). Data are shown as point-ranges with average over technical replicates ($n = 2$) as point and estimated standard deviation (over all data points) as line. Median (over 50 best parameter sets) simulations are shown as stacked areas with color corresponding to channels (blue: $BRAF^{V600E}$, orange: RAS). Shading indicates 80% percentiles over 50 best parameter sets.

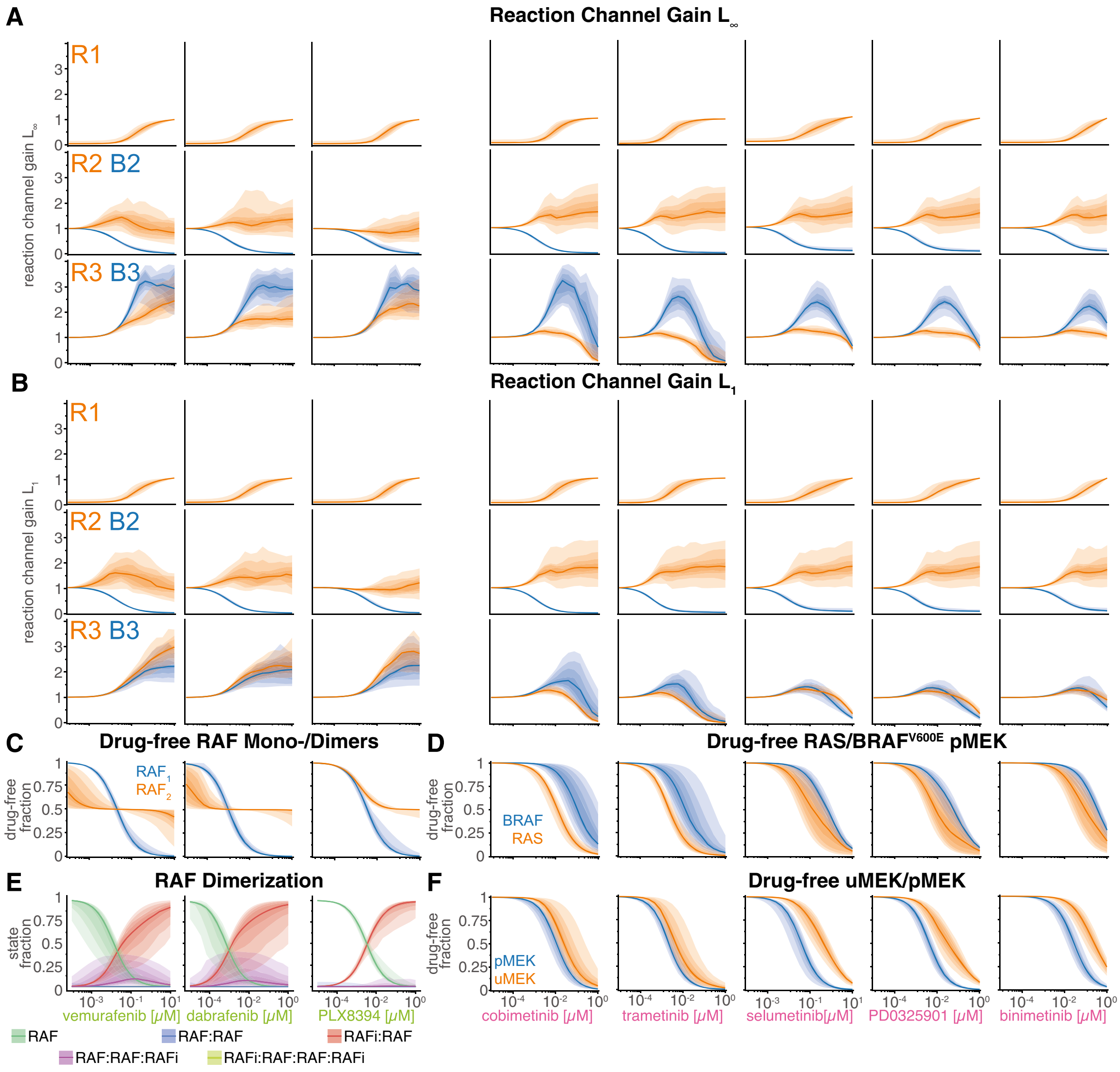

Figure EV4. Quantification of gain in RAS and BRAF^{V600E} channels (extended).

A, B Quantification of signal transmissions in terms of gain (L_1 and L_∞) along the edges of the simplified network in Fig 4A for different concentrations of three different RAF inhibitors (columns 1–3) and five different MEK inhibitors (columns 4–8). Color indicates the reaction channel (blue: BRAF^{V600E}, orange: RAS). Solid lines show median, and shading indicates 20, 40, 60 and 80% percentiles over 50 best parameter sets.

C, D Quantification of drug-free protomer fractions. Columns correspond to different RAFi/MEKi, as in A/B. Colors indicate different complexification (C), reaction channel (D) or post-translational states (F). Solid lines show median, and shading indicates 20, 40, 60 and 80% percentiles over 50 best parameter sets.

E Simulated Assembly of RAF-RAFi complexes in response to different RAFi. Each color corresponds to a different complex. Complex assembly was quantified for RAFi-adapted cells at 5 min after EGF stimulation. Solid line shows median, and shading indicates 20, 40, 60 and 80% percentiles over 50 best parameter sets.

F Quantification of drug-free protomer fractions. Columns correspond to different MEKi, as in A/B. Colors indicate different post-translational states. Solid lines show median, and shading indicates 20, 40, 60 and 80% percentiles over 50 best parameter sets.

 © 2023 The Authors

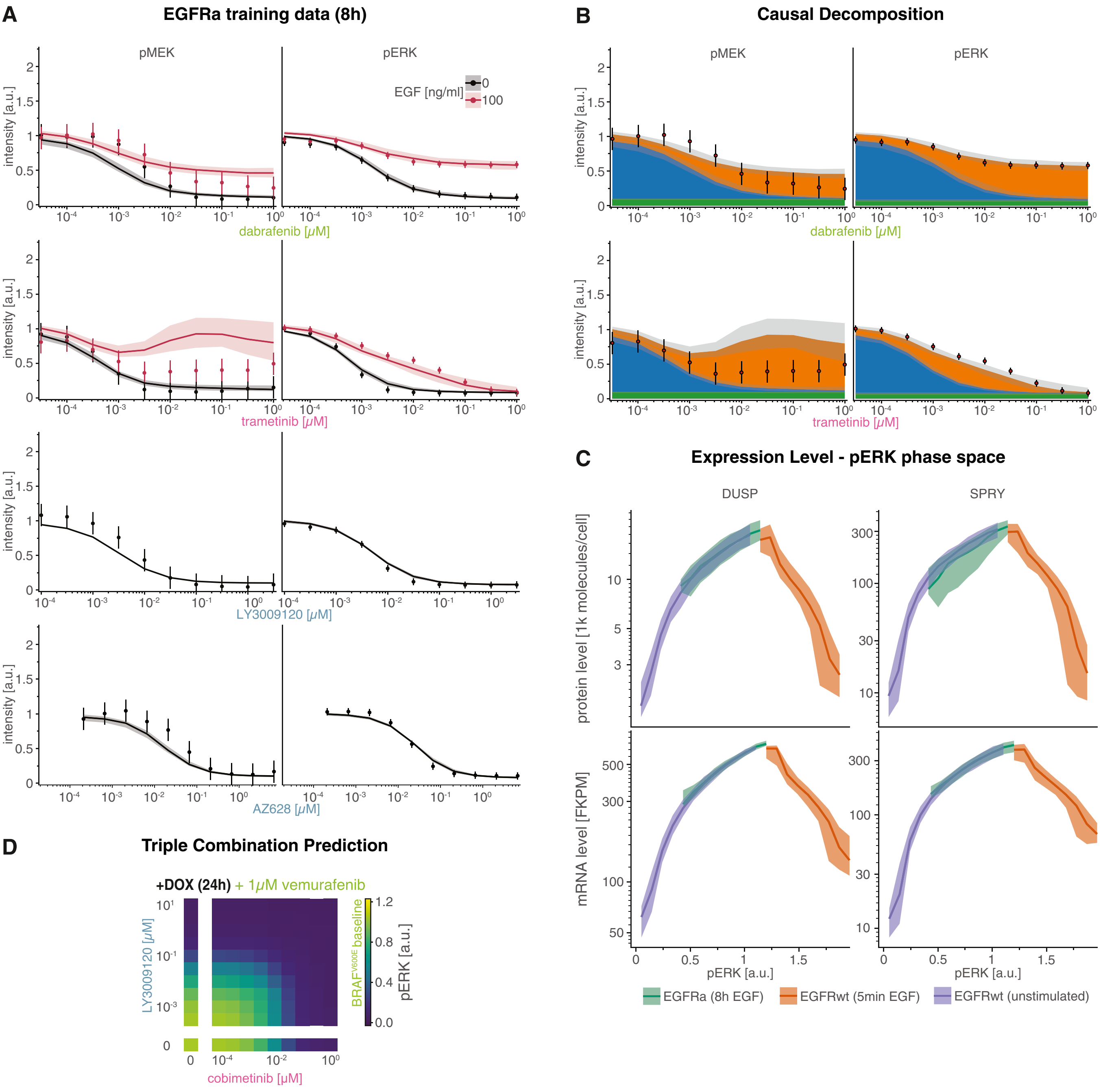

Figure EV5. Additional training data for EGFR upregulation and Causal Decomposition.

Experiments were performed in CRISPRa-EGFR A375 cell lines in 5% FBS medium after 24 h of drug adaptation. EGF stimulation was at 100 ng/ml. Data are shown as point-ranges with average over technical replicates ($n = 2$) as point and estimated standard deviation (over all data points) as line. Data from different experiments (biological replicates) are shown separately. Thick lines or stacked areas show median simulations and shading indicates 80% percentiles over 50 best parameter sets.

A Model simulations and experimental data for pMEK (left) and pERK (right) in EGF stimulated (8 h) and unstimulated conditions.

B Comparison of experimental data and decomposed model simulations at 5 min after EGF stimulation. Simulations are shown as stacked areas with color corresponding to channels (blue: $BRAF^{V600E}$, orange: RAS).

C Relationship between DUSP and SPRY expression levels and ERK phosphorylation levels under different experimental conditions (shown as different colors). pERK levels were binned into 20 equidistant discrete levels.

D Predicted dose response for combinations of LY3009120 and cobimetinib at 1 μM vemurafenib. Simulations were performed for $BRAF^{V600E}$ $NRAS^{Q61K}$ double mutant cells that were adapted to all three drugs.

© 2023 The Authors
