# Supplementary material for: Mechanistic model of MAPK signaling reveals how allostery and rewiring contribute to drug resistance
Source: Mol Syst Biol. 2023 Jan 26;19(2):e10988. doi: 10.15252/msb.202210988 (PMC9912026; doi:10.15252/msb.202210988)
Supplement: Supplementary file 4 — PDF+ [file MSB-19-e10988-s001.pdf]

Article

TRANSPARENT PROCESS

OPEN ACCESS

molecular systems biology

Mechanistic model of MAPK signaling reveals how allostery and rewiring contribute to drug resistance

Fabian Fröhlich[1,†], Luca Gerosa[1,†,‡], Jeremy Muhlich[1] & Peter K Sorger[1,*]

Abstract

BRAF is prototypical of oncogenes that can be targeted therapeutically and the treatment of BRAF^{V600E} melanomas with RAF and MEK inhibitors results in rapid tumor regression. However, drug-induced rewiring generates a drug adapted state thought to be involved in acquired resistance and disease recurrence. In this article, we study mechanisms of adaptive rewiring in BRAF^{V600E} melanoma cells using an energy-based implementation of ordinary differential equation (ODE) modeling in combination with proteomic, transcriptomic and imaging data. We develop a method for causal tracing of ODE models and identify two parallel MAPK reaction channels that are differentially sensitive to RAF and MEK inhibitors due to differences in protein oligomerization and drug binding. We describe how these channels, and timescale separation between immediate-early signaling and transcriptional feedback, create a state in which the RAS-regulated MAPK channel can be activated by growth factors under conditions in which the BRAF^{V600E}-driven channel is fully inhibited. Further development of the approaches in this article is expected to yield a unified model of adaptive drug resistance in melanoma.

Keywords allosteric interactions; drug resistance; kinetic modeling; MAPK pathway; rewiring
Subject Categories Cancer; Computational Biology; Signal Transduction
DOI 10.15252/msb.202210988 | Received 17 February 2022 | Revised 29 November 2022 | Accepted 15 December 2022 | Published online 26 January 2023
Mol Syst Biol. (2023) 19: e10988

Introduction

Eukaryotic signal transduction allows cells to regulate their growth, differentiation, and morphogenesis in response to external stimuli (Ullrich & Schlessinger, 1990; Hunter, 2000). In its reliance on receptor tyrosine kinase (RTK) autophosphorylation, assembly of signaling complexes on receptor tails, and activation of mitogen activated protein kinases (MAPKs; Box 1) signal transduction initiated by the binding of epidermal growth factor (EGF) to the EGF receptor (EGFR) is prototypical of growth-promoting signal transduction systems. The MAPK cascade comprises the RAF, MEK and ERK kinases, which regulate downstream factors such as ELK, ETS1 and AP1 transcription factors, as well as changes in cell motility and morphology (Lavoie *et al*, 2020). EGFR signaling has also been studied extensively using dynamical systems analysis (Starbuck & Lauffenburger, 1992; Kholodenko *et al*, 1999; Resat *et al*, 2003; Blinov *et al*, 2006; Chen *et al*, 2009; Gerosa *et al*, 2020) leading to better understanding of signal transduction in general as well as development of new modeling methods.

Oncogenic mutations are common in signal transduction networks and the V600E mutation in BRAF is an exemplar of these (Sanchez-Vega *et al*, 2018). In melanoma (Davies *et al*, 2002), thyroid cancer (Kebebew *et al*, 2007), colorectal cancer (Clarke & Kopetz, 2015), and other tissues, BRAF^{V600E} mutations cause constitutive activation of the MAPK pathway and oncogenic transformation. In cutaneous melanoma, inhibitors of the BRAF (BRAFi) and MEK (MEKi) kinases (e.g., vemurafenib and cobimetinib) are prototypical of highly effective targeted anti-cancer drugs (English & Cobb, 2002; Samatar & Poulikakos, 2014). A combination of BRAFi and MEKi is the current first-line treatment for metastatic melanoma (Sullivan & Flaherty, 2012) and frequently results in rapid tumor shrinkage. However, BRAF^{V600E} tumors usually develop resistance to RAFi/MEKi therapy within months to years, reducing long-term survival. The frequent and rapid rise of drug resistance in melanoma and the innate refractoriness of other MAPK-driven cancers to existing drugs has spurred extensive work aimed at understanding resistance mechanisms. Blocking the emergence of drug-resistant states is widely thought to be the key to achieving better patient outcomes with RAFi/MEKi drugs and precision oncology in general.

Resistance to MAPK inhibition occurs over a range of timescales. Adaptive resistance, which is reversible and does not involve acquisition or selection for mutations, can be observed within a few days of drug exposure (Fallahi-Sichani *et al*, 2017; Marin-Bejar *et al*, 2021; Oren *et al*, 2021). In cultured cells, adaptive resistance can last for months, giving rise to persister cells in which oncogenic BRAF signaling remains strongly inhibited but cells continue to grow, albeit more slowly than in the absence of drugs (Lito *et al*, 2012). In patients and in cultured cells, acquisition of recurrent mutations, commonly in RTKs or components (or regulators) of the MAPK cascade, leads to reactivation of MAPK signaling and unrestrained cell growth (Long *et al*, 2014; Shi *et al*, 2014). The relationship between adaptive and acquired resistance is not fully

1 Laboratory of Systems Pharmacology, Department of Systems Biology, Harvard Medical School, Boston, MA, USA
*Corresponding author. Tel: +1 617 432 6902; E-mail: peter_sorger@hms.harvard.edu
†These authors contributed equally to this work
‡Present address: Genentech, Inc., South San Francisco, CA, USA

© 2023 The Authors. Published under the terms of the CC BY 4.0 license.

Box 1. The core of the MAPK pathway is a three-enzyme cascade comprising RAF–MEK–ERK kinases (HUGO: ARAF/BRAF/RAF1, MAP2K1/MAP2K2, and MAPK1/MAPK3) that transduces signals from extracellular stimuli, most commonly growth factors and receptor tyrosine kinases (RTKs; Lavoie *et al*, 2020). Three-enzyme cascades involving closely related kinases also transmit signals from cytokines and their receptors. Driving oncogenic mutations are found in multiple components in or upstream of the MAPK pathway (Burotto *et al*, 2014), commonly KRAS (G12C/D/V, G13C/D), NRAS (Q61H/K; Prior *et al*, 2012), BRAF (V600E/K) and less commonly MEK and ERK (Gao *et al*, 2018). $BRAF^{V600E}$ or closely related mutations (e.g., $BRAF^{V600K}$) are found in ~50% of cutaneous melanomas and RAF/MEK therapy is the first line treatment option for BRAF-mutant metastatic melanoma (Flaherty *et al*, 2012). BRAF mutations are also found in ~10% of colorectal cancers and several other tumor types (Davies *et al*, 2002), but RAF/MEK therapy is rarely effective in these settings.
The binding of growth factors to RTKs induces their intracellular auto-phosphorylation, followed by association of SH2 and SH3-containing proteins with phosphorylated tyrosine residues on receptor tails. Subsequent signalosome assembly involves adaptor proteins such as GRB2, enzymes that modify second messengers such as PI3Ks, and guanine nucleotide exchange factors (GEFs) such as SOS1 (Lemmon & Schlessinger, 2010). GEFs convert one or more of the N, K, and H RAS GTPases (depending on cell type) into the active GTP-bound form, and GTP-bound RAS then activates the ARAF/BRAF/RAF1 kinases by recruiting them to the plasma membrane and inducing their dimerization. BRAF/RAF1 homo- and heterodimers are the primary mediators of MEK phosphorylation (ARAF has low kinase activity). Phosphorylated and active MEK then phosphorylates ERK on two proximate residues. Both phosphorylation steps are potentiated by the assembly of multi-protein complexes involving 14-3-3 and KSR scaffolding proteins (Lavoie & Therrien, 2015). Active ERK phosphorylates transcription factors, cytoskeletal proteins, and other kinases and is the proximate functional output of the MAPK cascade. Changes in the levels or activities of proteins such as DUSP4/6 phosphatases, which remove activating phosphorylation modifications, and SPRY2/4 proteins, which sequester GRB2, as well as inhibitory phosphorylation of EGFR, SOS1 and RAF act as negative-feedback mechanisms and enforce homeostatic control over MAPK activity.

understood and is an area of active investigation (Shaffer *et al*, 2017; Schuh *et al*, 2020). However, it has been proposed that DNA replication may be less faithful, or DNA damage responses less effective, in adapted than drug-naïve cells, leading to an accumulation of resistance mutations (Shaffer *et al*, 2017; Russo *et al*, 2019; Schuh *et al*, 2020).

A paradox of the drug adapted state in $BRAF^{V600E}$ mutant melanoma is that MAPK activity is essential for proliferation of this cell type and yet oncogenic BRAF signaling remains strongly inhibited. Analysis of cell-average MAPK levels led to the suggestion that partial MAPK rebound (to ~5 to 20% of the kinase activity in drug-naïve cells) is sufficient for cell survival and proliferation (Lito *et al*, 2012). However, more recent single-cell studies show that adapted cells experience sporadic MAPK pulses of ~90 min duration and that these pulses are sufficient for cyclin D transcription and passage of a subset of cells into S phase (Gerosa *et al*, 2020). Pulses appear to arise from growth factors that act in an autocrine/paracrine manner by binding to EGFR and other RTKs expressed on persister cells. This finding raises a further question: how precisely can oncogenic MAPK signaling be repressed while receptor-mediated MAPK signaling remains active? The accepted explanation is that the cell signaling has become "rewired" in adapted cells (Lee *et al*, 2012; Ding *et al*, 2018; Wei *et al*, 2020).

In the absence of a new mutation, rewired networks are postulated to transmit or propagate oncogenic signals by different combinations or activity states of cell signaling proteins than drug-naïve networks. In some cases, rewiring is thought to involve a switch from one mitogenic pathway to another, from MAPK to PI3K-AKT signaling, for example, but in drug resistant melanoma, the same MAPK components appear to be essential in the original and rewired states. More generally, rewiring is one of the several concepts in translational cancer biology that are intuitively plausible, but have not yet been subjected to quantitative, mechanistic modeling and analysis.

One way to gain deeper insight into rewiring at a mechanistic level is to perform the type of dynamical systems analysis that has previously proven effective in the study of RTK-MAPK signaling (Kholodenko *et al*, 1999; Chen *et al*, 2009; Schöberl *et al*, 2009; Kholodenko, 2015; Rukhlenko *et al*, 2018). This commonly involves constructing networks of ordinary differential equation (ODEs) to represent the precise temporal evolution of signal transduction networks under different conditions. ODEs are a principled way to represent cellular biochemistry in a continuum approximation and, with the addition of "compartments", can also model the assembly of multi-protein complexes and transport between cellular compartments (Aldridge *et al*, 2006). In the case of the A375 melanoma cells used in this study, quantitative proteomics shows that proteins in the MAPK pathway are present at 10^2 to 10^4 molecules per cell (Gerosa *et al*, 2020), so continuum mass-action models represent an appropriate approximation (conversely, intrinsic noise is expected to be low).

Combinatorial complexity represents a substantial challenge to modeling even relatively restricted sets of signaling proteins. The presence of multiple reversible, post-translational modifications, protein–protein, and protein-small molecule interactions often makes the number of distinct biochemical species 10–1,000 fold greater than the number of gene products (Faeder *et al*, 2005; Box 3). Rule-based modeling was developed specifically to address this challenge and uses abstract representations of binding patterns and reactions to describe combinatorically complex networks in a compact programmatic formalism. Rules automatically generate ODE networks describing diverse types of reactions and molecular assemblies (Faeder *et al*, 2005; Hlavacek *et al*, 2006; Lopez *et al*, 2013) for subsequent model calibration and exploration.

An additional challenge in modeling MAPK signaling is that it involves allosteric regulation, in which the affinities of RAS, RAF and small molecules for each other are determined by protein conformation and oligomerization state. In conventional ODE modeling, a large number of parameters are necessary to describe the dependency of such affinities on states of assembly. However, protein–protein and protein–small molecule binding and unbinding does not consume energy and thermodynamic formalisms that impose energy conservation provide rigorous means to constrain the number of binding parameters to a minimal, principled set (Box 3; Ollivier *et al*, 2010; Sekar *et al*, 2016). The use of thermodynamics to derive kinetic rates was pioneered by Arrhenius (1889) and subsequently

 © 2023 The Authors

derived independently by Eyring (1935), Evans and Polanyi (1935), but it is only recently that practical approaches have emerged for using thermodynamic formalisms in reaction models (Olivier *et al*, 2005; Honorato-Zimmer *et al*, 2015; Kholodenko, 2015; Gawthrop & Crampin, 2017; Mason & Covert, 2018; Rukhlenko *et al*, 2018; Klosin *et al*, 2020; Gollub *et al*, 2021). Applications of these methods to signal transduction remain limited, in part because of the complexity of relevant models, but Kholodenko and colleagues have pioneered the application of thermodynamic balance to MAPK signaling (Rukhlenko *et al*, 2018).

Model calibration and non-identifiability represents a final challenge in modeling networks of readily reversible reactions. Model calibration (estimating parameter values that minimize the deviation from experimental data) is compute-intensive (Fröhlich *et al*, 2017) and even after calibration, parameters can assume wide ranges, a property known as non-identifiablity (Chis *et al*, 2011; Raue *et al*, 2011; Kreutz *et al*, 2012; Wieland *et al*, 2021). When models are combinatorically complex and non-identifiable, it can be difficult to quantify fluxes, explain how signaling state arise and trace how species of interest are created by upstream reactions and consumed downstream. This complicates the quantification of signal propagation through the reaction network, a prerequisite for the investigation of concepts of such as network rewiring.

In this article, we described a second-generation MAPK Adaptive Resistance Model (MARM2.0) that seeks to explain the rewiring of EGFR/MAPK signaling occurring in drug adapted $BRAF^{V600E}$ melanoma cells. MARM2.0 builds on a large body of structural, biochemical and theoretical work on EFGR/MAPK signaling and feedback regulation (Solit *et al*, 2006; Poulikakos *et al*, 2010; Lito *et al*, 2012, 2013; Hatzivassiliou *et al*, 2013; Haling *et al*, 2014; Yao *et al*, 2015) and is constructed using rule-based modeling in PySB with thermodynamic balance. By developing and applying a new approach to causal tracing that facilitates the analysis of "signal flow" in large ODE model, we show how rewiring alters the organization and amplification/attenuation characteristics of multiple reaction channels operating in parallel in the MAPK cascade. We describe how rewiring, in conjunction with a timescale separation between signal transduction and transcriptional feedback, generates a drug adapted state in which the RAS-regulated MAPK channel can be active under conditions in which the $BRAF^{V600E}$-driven channel is fully inhibited. Additionally, we show that how the concept of a reaction channel provides an intuitive explanation for resistance to RAF and MEK inhibitors individually and in combination in different BRAF mutant cancers.

Results

A structure-based model of EGFR and ERK signaling

The MAPK signaling cascade (Box 1) and its immediate regulators constitute no more than two dozen unique gene products, but the binding of these proteins to each other gives rise to a remarkably large number of molecular species, many of which have distinct activities. Moreover, the complexity of the MAPK cascade increases substantially when we consider states that are bound and unbound to drugs. For example, BRAF/CRAF can exist in monomeric, homo- and heterodimeric forms, with either one or two subunits bound to RAFi, each with or without RAS-GTP bound as an activator. Drug binding occurs preferentially to some BRAF oligomers and not others (Box 2), and can strongly influence association with upstream and downstream factors. To recapitulate the responses of

Box 2. Multiple small molecule inhibitors targeting individual MAPK kinases are FDA approved, but combinations of RAF and MEK inhibitors are the most widely used clinically. A subtle relationship exists between the mechanism of action of these drugs, kinase conformation, and formation of mutli-protein complexes. In the absence of upstream stimuli, RAF kinases are found in cells as monomers; activation by RAS-GTP causes dimerization. Some activating BRAF mutations (Yao *et al*, 2015) and splice variants (Poulikakos *et al*, 2011) also promote dimerization, but $BRAF^{V600E/K}$ kinases are constitutively activated without requiring dimerization. Whether RAF is present as a monomer, heterodimer or homodimer profoundly influences the enzyme's sensitivity to inhibition (Yao *et al*, 2015). The FDA-approved RAF inhibitors vemurafenib, dabrafenib, and encorafenib are ATP-competitive type I½ kinase inhibitors (Roskoski, 2016) that preferentially bind to the alpha-C helix-out, DFG-in conformation assumed by $BRAF^{V600E/K}$; this state differs from the alpha-C helix-in (and DFG-in) state found in activated wild-type RAF (Karoulia *et al*, 2017), whereas binding of type I½ BRAF inhibitors to $BRAF^{V600E/K}$ inactivates the enzyme, binding to wild-type RAF monomers promotes kinase dimerization and activation, leading to amplification of MAPK signaling, a phenomenon termed paradoxical activation (Hall-Jackson *et al*, 1999; Hatzivassiliou *et al*, 2010; Poulikakos *et al*, 2010). To prevent this, "paradox breaker" RAF inhibitors such as PLX8394 have been developed (Zhang *et al*, 2015; Tutuka *et al*, 2017; Yao *et al*, 2019). These are type I½ inhibitors that, by virtue of locking the R506 side-chain in the out conformation, do not promote dimerization (Karoulia *et al*, 2017). Both regular and paradox breaker type I½ inhibitors have a lower affinity for the 2nd protomer in a RAF dimer, which typically assumes the inactive alpha-C helix-in, DFG-out conformation. Thus, the structural differences between monomers and dimers (rather than mutations in the ATP binding pocket) are the basis of the selectivity of clinically approved RAF inhibitors for cells transformed by BRAF mutant kinases. However, the inability of type I½ inhibitors to fully inhibit homo- and hetero-dimer RAF kinases is a primary mechanism of drug resistance in cancers with sustained RAS-GTP signaling, including EGFR-driven signaling in $BRAF^{V600E/K}$ colorectal cancer. In contrast, so-called "panRAF" type II inhibitors, such as the Phase 1 compound LY3009120 (Peng *et al*, 2015) and preclinical compound AZ-628 (Noeparast *et al*, 2018), bind RAF in the alpha-C helix-in, DFG-out conformation and inhibit both RAF protomers with similar potency. These inhibitors can achieve more complete MAPK suppression but appear to cause additional toxicity, presumably by interfering with MAPK activity in non-cancer cells. Multiple type II inhibitors are currently under clinical investigation for solid tumors (Yen *et al*, 2021), including melanoma, but, so far, none have been approved for use in humans.

FDA-approved MEK inhibitors such as cobimetinib, trametinib and binimetinib, are type III non-ATP competitive (allosteric) inhibitors that lock the MEK kinase in a catalytically inactive state, limit movement of the activation loop, and decrease phosphorylation by RAF (Wu & Park, 2015). Most of these MEK inhibitors are more potent at preventing ERK activation by $BRAF^{V600E/K}$ than by RAF acting downstream of mutant RAS (Hatzivassiliou *et al*, 2013; Lito *et al*, 2014) or RTKs (Gerosa *et al*, 2020). The reasons for this are not fully understood, but are thought to be inhibitor specific and include mechanisms such as the lower affinity of MEK inhibitors for phosphorylated when compared with unphosphorylated MEK, and differences in RAF–MEK binding (Hatzivassiliou *et al*, 2013; Pino *et al*, 2021).

© 2023 The Authors

cells to RAFi in a mechanistic computational model, it is necessary for the allosteric interactions that control association of RAF with upstream and downstream factors and with RAFi to be described in detail (Rukhlenko *et al*, 2018).

To accomplish this, we generated a compartmentalized ODE model of MAPK signaling (the MAPK Adaptive Resistance Model MARM2.0) that extends a simpler and recently published model (MARM1.0). MARM1.0 was used in an experimental study we recently published (Gerosa *et al*, 2020) that uses modeling as an explanatory tool but does not involve any model analysis. Such analysis is the focus of the current article and its updated model. MARM2.0 was calibrated using data described in Gerosa *et al* with the addition of drug-response data that is unique to the current study. Moreover, both MARM1.0 and MARM2.0 build on an earlier model of RAF-RAFi interaction developed by Kholodenko (2015), but with the inclusion of more proteins and complexes. Model expansion was greatly facilitated by the use of rule-based BNG models in the domain-specific Python language PySB (Blinov *et al*, 2004; Lopez *et al*, 2013). More specifically, MARM1.0 & 2.0 extend the RAF–MEK–ERK model of Kholodenko with the addition of upstream activation and multiple feedback mechanisms relevant to acquired resistance to RAF inhibitors (Lito *et al*, 2012) and a more detailed description of MAPK enzymes themselves (Fig 1A). Compared with MARM1.0, MARM2.0 is compartmentalized (compartments: *extracellular space*, *plasma membrane*, *cytoplasm* and *endosomal membrane*), it adds EGFR-CBL interaction and endosomal recycling, and includes mRNA species in the description of transcriptional feedback control; it also accounts for the direct inhibitory action of ERK on RAF, a reaction omitted in MARM1.0. In total, MARM2.0 has 17 distinct molecular species: 11 proteins, three mRNA species and three small molecule inhibitor classes. Proteins include EGFR, BRAF, CRAF, MEK and ERK, the dual specificity phosphatase DUSP, guanine nucleotide exchange factor SOS1, GTPase RAS, E3 ubiquitin ligase CBL, adaptor protein GRB2, and RTK negative regulator SPRY (ellipses in Fig 1A). EGF, RAFi, panRAFi and MEKi, (depicted as colored circles and rounded boxes in Fig 1A) are optionally present and values for kinetic and energetic parameters can be set so that the inhibitors can correspond to any of 10 different small molecules that are used as human therapeutics or pre-clinical tools. These comprise the RAFi compounds vemurafenib, dabrafenib, PLX8394, the panRAFi (Box 2) compounds LY3009120 and AZ628, and MEKi compounds cobimetinib, trametinib, selumetinib, binimetinib and PD0325901.

To maintain model tractability, we lumped together paralogs, combined phosphorylation sites having similar functions, and simplified other aspects of EFGR regulation, which exhibits particularly high combinatorial complexity (Blinov *et al*, 2006). MARM2.0 nonetheless has over 10^5 biochemical reactions, illustrating how transient binding (binding interactions are summarized in Fig 1B) among a few kinases, their regulators, and inhibitory drugs generates an elaborate biochemical network. With respect to paralogs, we made the following assumptions: "RAS" stands in for KRAS, NRAS, and HRAS, "MEK" for MAP2K1 and MAP2K2, "ERK" for MAPK1 and MAPK3, "DUSP" for DUSP4 and DUSP6, and "SPRY" for SPRY2 and SPRY4 (lumping of paralogs is depicted in Fig 1A by thick outlines). This is equivalent to assuming that all paralogs have the same kinetic rate constants. In some cases, paralogs are known to be very similar (e.g., MAPK1, MAPK3) but in other cases they are functionally distinct (e.g., KRAS, NRAS and HRAS). The three RAS paralogs are expressed at similar levels in A375 cells and we did not distinguish among them because we do not yet have relevant training data. However, MARM could easily be modified for future studies that focus on differences between RAS species. We did not lump BRAF and CRAF into a single RAF species due to the unique role that $BRAF^{V600E}$ plays as an oncogene; ARAF was omitted due to its low kinase activity. We also lumped together multi-site phosphorylation of EGFR (on Y1068, Y1086, Y1173, etc.), MEK (MAP2K1: S218, S222; MAP2K: S222, S226) and ERK (MAPK1: T185, Y187; MAPK3: T202, Y204) as single post translational modifications for each protein. The underlying phosphorylation reactions were implemented as two-step reactions comprising substrate binding and phosphorylation steps. Finally, mRNA species were included for DUSP, EGFR and SPRY to model transcriptional feedback with distinct, lumped translation rates for each species (depicted by dark green arrows in Fig 1A). This made it possible to calibrate models on time-course and dose–response transcriptomic data.

To model RTK-induced MAPK activation, we focused on EGFR autophosphorylation at Y1068, Y1086 and Y1173, which creates GRB2 binding sites (Batzer *et al*, 1994) as well as EGFR ubiquitination by CBL (Alwan *et al*, 2003) and subsequent endocytosis and recycling. EGFR endocytosis and recycling rates were dependent on EGFR levels, as previously described (Starbuck & Lauffenburger, 1992; Resat *et al*, 2003). The "addition" of EGF to MARM2.0 promotes EGFR dimerization and trans-phosphorylation, recruitment of GRB2-SOS1 complexes to phospho-tyrosine residues on receptor tails and consequent GTP loading and activation of RAS. Receptors are then subjected to endocytosis leading to either their degradation or recycling. GTP-loaded RAS (RAS-GTP) promotes RAF dimerization and initiates the RAF–MEK–ERK (MAPK) cascade (Box 1). When $BRAF^{V600E}$ is present, it constitutively phosphorylates MEK in the absence of upstream signals. Phosphorylated MEK (pMEK) phosphorylates ERK (pERK), which indirectly upregulates expression of proteins that act as negative regulators of RTK signal transduction (these intermediate steps are represented as lumped reactions). Multiple negative regulatory mechanisms are known, and we modeled five of them. Three involved transcriptionally-mediated changes in protein abundance for (i) EGFR itself, (ii) DUSP, which antagonize ERK signaling by dephosphorylating the T and Y residues in the T-Y-X motif in the ERK activation loop (Saha *et al*, 2012) and (iii) SPRY, which has multiple biochemical activities, among which we modeled sequestration and inactivation of GRB2 (Lao *et al*, 2006). Two involved phosphorylation mediated changes in protein–protein interactions, namely (iv) SOS1 binding to GRB2 and (v) RAF dimerization. SOS1 is phosphorylated on S1134 and S1161 sites by RSK creating a 14-3-3 docking site, which sequesters the protein in an inactive conformation (Corbalan-Garcia *et al*, 1996; Kamioka *et al*, 2010). RSK is transcriptionally and post-translationally activated by ERK, but we represented this with a single pERK dependent phosphorylation reaction and a phosphorylation dependent energy pattern that modulates SOS1-GRB2 affinity. CRAF and BRAF are phosphorylated by ERK on multiple residues, decreasing their affinity for RAS and other RAF molecules (Dougherty *et al*, 2005; Ritt *et al*, 2010). Guided by previous models of this process (Rukhlenko *et al*, 2018), we implemented a single, pERK dependent phosphorylation of RAF monomers that involves energy patterns that control the affinity between RAF monomers and

 © 2023 The Authors

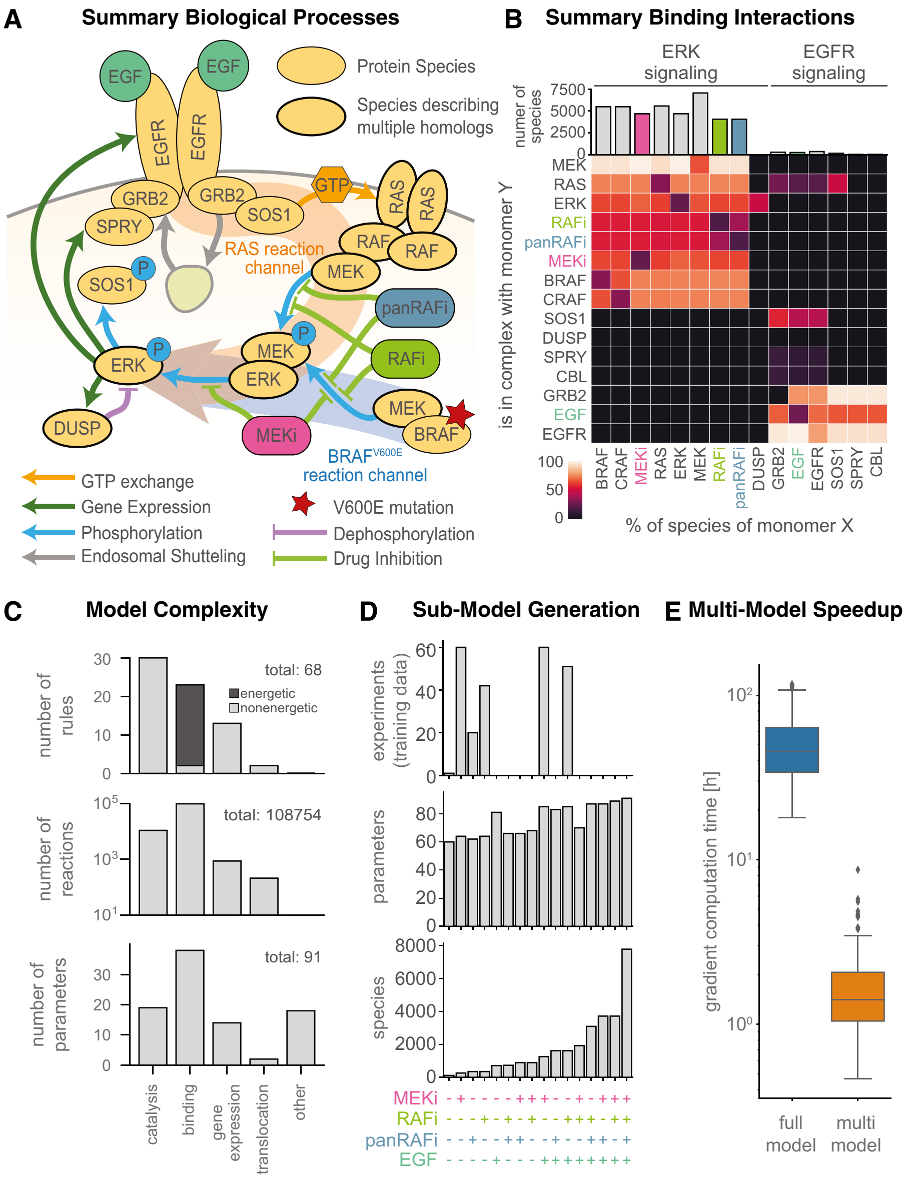

Figure 1. Thermodynamic model of EGFR and ERK signaling.

A Schematic overview of processes described in the model.

B Summary of model species and oligomerization in the model. Coloring of tiles indicates percentage with respect to total of monomer species (per row). Columns for the drug and growth factor perturbations RAFi, panRAFi, MEKi and EGF are highlighted according to the respective color in (A).

C Statistics of model rules, reactions and parameters. Catalysis includes (de-) phosphorylation, GTP-exchange and (de-)ubiquitination. Other parameters include initial conditions and scaling factors and background intensities.

D Number of experiments and sizes of respectively resized models according to the multi-model optimization scheme. A plus on the bottom indicates that the respective perturbation was applied in the corresponding experiment, color as in (A/B).

E Comparison of gradient computation time for the full-model and multi-model optimization approach. Central band shows media, box extends from lower to upper quartile values and whiskers show full range excluding outliers (points more than 1.5 interquartile ranges away from lower and upper quartiles).

between RAF and RAS (Box 4). To describe allosteric drug interactions involving RAFi and panRAFi, we included energy patterns for RAFi-RAF_2 trimers and $RAFi_2$-RAF_2 tetramers. For MEKi, we included a phosphorylation dependent energy pattern for MEKi-MEK interaction (Box 2) but no energy patterns for allosteric RAF–MEK-MEKi complexes. This means that we did not model disruption of RAF–MEK interaction by MEKi since it has been reported that this is not the mechanism determining the potency of the MEK inhibitors in our study (Pino *et al*, 2021). Instead, we used a scaling factor (whose value was determined during model calibration) to encode a reduction in the rate of phosphorylation of MEK-MEKi complexes, when compared with apo-MEK, by $BRAF^{V600E}$. This approach captures MEKi-mediated inhibition of MEK phosphorylation by $BRAF^{V600E}$ in cell lines by one or more of the several structurally

© 2023 The Authors

related mechanisms reported in the literature. A comprehensive description of these implementations, and of MARM2.0 more generally, is provided in the Code EV1 as a Jupyter Notebook (Model Documentation.ipynb).

MARM 2.0 includes 68 rules and 91 free parameters (kinetic rates, energies, scaling factors, etc.; total 115 free parameters when MARM2.0 is instantiated for all of 10 small molecule RAF/MEK inhibitors). Six rules described transcript turnover, seven protein turnover, 24 phosphorylation, 23 binding and three sets of two rules each described GTP/GDP exchange, ubiquitination, and translocation between cellular compartments (Fig 1C). For example, the binding rule "*Rule('BRAF_and_uMEK_bind_and_dissociate', BRAF (mek=None) + MEK(phospho='u', raf=None) ¦ BRAF(mek=1) % MEK(phospho='u', raf=1), ...)*" describes binding of BRAF to unphosphorylated MEK (uMEK), a prerequisite for MEK phosphorylation. Binding requires MEK to be unphosphorylated (*phospho = 'u'*), but does not specify any dependence on RAS, BRAF, CRAF or RAFi. Implementation of PySB rules generated > 7,700 molecular species and > 100,000 biochemical reactions with most proteins participating in > 4,000 species, a reflection of the combinatorial complexity described above. Binding rules accounted for > 85% of all reactions in the model (96,874 of 108,754 reactions total) and > 90% (21/23) of these binding rules were formulated as "energetic rules" with binding affinities expressed in terms of normalized Gibbs free energy differences (ΔG; Box 3). Binding and unbinding rates were then computed according to the Arrhenius law. To facilitate programmatic model formulation within an energetic framework, we implemented support for the eBNG framework (Hogg, 2013; Harris *et al*, 2016) in PySB. This enabled specification of allosteric interactions using differences in free energy differences (ΔΔG, Box 3), which is a principled way of establishing context dependent binding and unbinding rates (with the balance encoded by the parameter ϕ).

ODE description of ERK pulsing enabled use of population average and Perturbational experiments to describe the behavior of single cells

Imaging studies have established that the A375 $BRAF^{V600E}$ melanoma cell line used in this study enters a drug adapted condition within 24 h of exposure to RAFi and/or MEKi and that this state is retained for at least 2–4 days, allowing it to be approximated as pseudo steady state (Gerosa *et al*, 2020). Unless explicitly stated otherwise, data were collected after a 24 h period of drug adaptation and model simulations were pre-equilibrated to these conditions. Once adapted to RAFi, $BRAF^{V600E}$ melanoma cells experience transient pulses of ERK activity at irregular intervals, consistent with a stochastic regulatory mechanism (Gerosa *et al*, 2020). In principle, BNG/PySB models can be instantiated as stochastic, agent-based systems to represent such stochastic fluctuations (Sneddon *et al*, 2011). However, the reactions in MARM2.0 involve sufficiently abundant proteins (~10^2 to 10^6 copies per cell) that intrinsic stochasticity is not expected to arise spontaneously. Thus, the irregular pulsing by drug adapted A375

Box 3. Changes in protein assembly and conformation, often mediated by post-translational modification, are the structural basis for much of signal transduction. For example, generating the active conformation of CRAF requires both N-terminal phosphorylation and association with a second RAF family member to stabilize the active state. Because formation of protein–protein interactions does not consume energy, a strict relationship exists between conformation and binding affinity (Tsai & Nussinov, 2014): when binding increases the stability of a specific conformational state, that state will also have higher binding affinity for its interacting partner. Since this relationship is transitive, binding affinities can be coupled through conformational states, giving rise to long-range, higher-order dependencies in oligomeric complexes. Such higher-order dependencies can create ultrasensitive responses, which are often involved in cell fate decisions or homeostasis.

A conformational state is defined by a specific local minimum in the Gibbs free energy landscape. The relative stability of a conformational state S can be expressed as free energy difference ΔG_c with respect to a reference state S_0. Stabilizing or destabilizing conformational states is equivalent to changes in this free energy difference (i.e., $\Delta\Delta G_c$). Similarly, binding reactions can be characterized by the difference ΔG_b between the Gibbs free energies of binding educts and binding products, which is proportional to the logarithm of their dissociation constant K: $\Delta G_b = -RTlog(K)$, where R is the gas constant and T is the temperature. Energy conservation guarantees that a ligand (L)-induced change to the free energy of a conformational state S ($\Delta\Delta G_c$) is equal to the difference $\Delta\Delta G_b$ in the affinity of L for S when compared with S_0. This equilibrium description can be extended to dynamic behavior by means of the Arrhenius Equation (Arrhenius, 1889), which defines reaction propensities according to the free energy of the transition state (Sekar *et al*, 2016). Such an energy-based formulation enforces Wegscheider–Lewis cycle conditions on kinetic parameters (Wegscheider, 1911), ensuring detailed balance for equilibrium states, but also constraining dynamics of non-equilibrium processes. By ensuring energy conservation, the effective number of parameters needed to describe multimeric oligomerization processes is reduced (Kholodenko, 2015) and rigorous constraints are placed on the structures of models describing species that adopt multiple conformational states.

Energy conservation provides a natural framework for the specification of structure-based kinetic models that include allosteric interactions (Rukhlenko *et al*, 2018) and has been incorporated into a rule-based modeling form as energy-BioNetGen (eBNG; Sekar *et al*, 2016). In eBNG, allosteric interactions are encoded using energy patterns that permit specification of $\Delta\Delta G_b$. For example, a kinetic model for the binding of RAF inhibitors (RAFi in text, I in figure) to RAF kinases (RAF in text, R in figure; Box 4A) can be constructed using one rule for RAF dimerization (turquoise) and another for drug binding to RAF (black), which generates 12 reversible reactions (Box 4B). Allostery for drug binding to the 1st or 2nd protomer of a RAF dimer is imposed using the thermodynamic factors f (orange) and g (purple), which change $\Delta\Delta G_b$ via two energy patterns. The contribution of these thermodynamic factors to kinetic rates is exemplified by the relationship between Gibbs free energies and rate constants for RAF dimerization that are RAFi-dependent (Box 4C; no RAFi, black; one RAFi, orange; two RAFi purple). The parameter ϕ, controls whether $\Delta\Delta G_b$ influences educt states (ϕ = 0) or product states (ϕ = 1, depicted in C) or a mixture (0 < ϕ < 1). Using PySB, all 12 reactions depicted in Box 4B can be specified using two rules and four energy-patterns (Box 4D). Thus, PySB code automatically generates symbolic reaction rates that parameterize the reaction network according to allosteric effects whose magnitudes are set by the thermodynamic factors f and g (Box 4E). In this way, models of complex drug-protein interactions, such as resistance mediated by formation of RAF dimers, can be easily parameterized in terms of the baseline equilibrium constant for RAF dimerization (K_{RR}). We illustrated this by simulations with f = 0.001 and g = 1,000 (Box 4F) which represent a type I½ RAF inhibitor that avidly binds the 1st RAF protomer but has a 10^6-fold lower affinity for the 2nd protomer in a RAF dimer.

 © 2023 The Authors

Box 4.

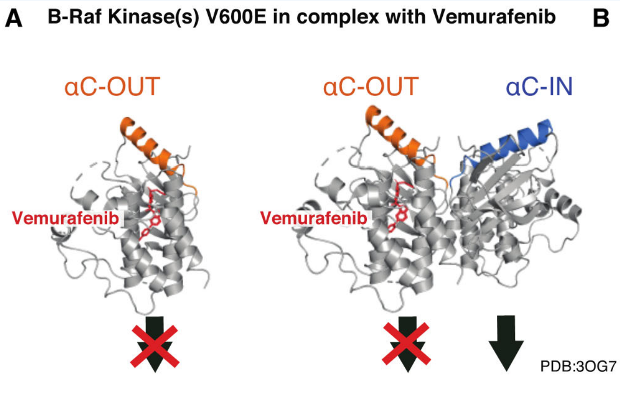

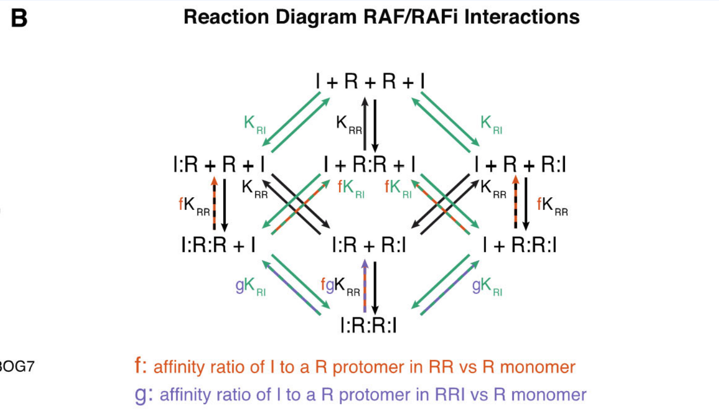

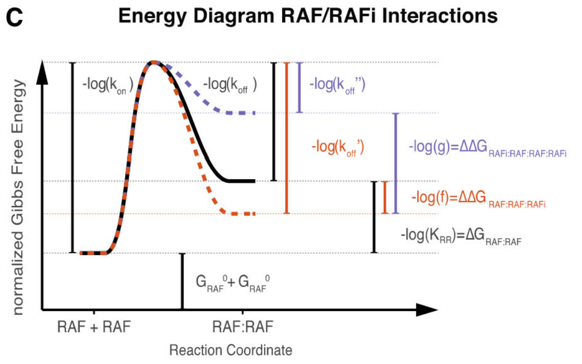

D PySB Implementation

```
Rule('RR', R(r=None) + R(r=None) | R(r=1) % R(r=1), phi_RR, Ea0_RR, energy=True)
Rule('RI', R(i=None) + I(r=None) | R(i=1) % I(r=1), phi_RI, Ea0_RI, energy=True)

EnergyPattern('ep_RR', R(r=1) % R(r=1), log(K_RR))
EnergyPattern('ep_RI', R(i=1) % I(r=1), log(K_RI))
EnergyPattern('ep_RRI', R(r=1, i=None) % R(r=1, i=2) % I(r=2), log(f))
EnergyPattern('ep_IRRI', I(r=3) % R(r=1, i=3) % R(r=1, i=2) % I(r=2), log(f) + log(g))
```

E Reaction rates

Rule	Reaction		Forward rate	Reverse rate	Dissociation constant
RR	R + R	RR	$0.5 \cdot k_{RR}$	kr_{RR}	$\frac{2.0 \cdot kr_{RR}}{k_{RR}}$
RI	R + I	RI	k_{RI}	kr_{RI}	$\frac{kr_{RI}}{k_{RI}}$
RR	RI + R	RRI	$f^{-\phi_{RR}} \cdot k_{RR}$	$f^{1-\phi_{RR}} \cdot kr_{RR}$	$\frac{f \cdot kr_{RR}}{k_{RR}}$
RR	RI + RI	IRRI	$0.5 \cdot k_{RR} \cdot \left(\frac{1}{f \cdot g}\right)^{\phi_{RR}}$	$kr_{RR} \cdot (f \cdot g)^{1-\phi_{RR}}$	$\frac{2.0 \cdot f \cdot g \cdot kr_{RR}}{k_{RR}}$
RI	RR + I	RRI	$2.0 \cdot f^{-\phi_{RI}} \cdot k_{RI}$	$f^{1-\phi_{RI}} \cdot kr_{RI}$	$\frac{0.5 \cdot f \cdot kr_{RI}}{k_{RI}}$
RI	RRI + I	IRRI	$g^{-\phi_{RI}} \cdot k_{RI}$	$2.0 \cdot g^{1-\phi_{RI}} \cdot kr_{RI}$	$\frac{2.0 \cdot g \cdot kr_{RI}}{k_{RI}}$

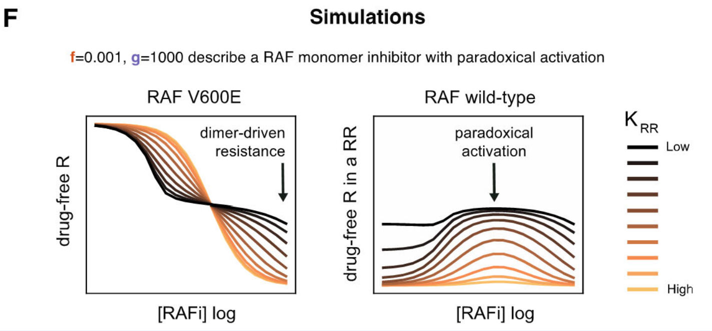

(A) Protein structures of monomeric and dimeric $BRAF^{V600E}$ protomers bound to vemurafenib. (B) Binding diagram for RAF and RAFi molecules. Formulas next to reaction arrows indicate the dissociation constants of the respective reactions. Arrow color indicates type of reaction (black: RAF dimerization, turquoise: RAFi binding). Dashed line color indicates the thermodynamic parameters that modulate the respective reactions (orange: f, purple: g). (C) Illustration of relationship between Gibbs free energies and kinetic rates for RAF dimerization. Modulation of kinetic rates through a context specific energy patterns that depends on the number of bound RAFi molecules is indicated in orange (one RAFi bound, parameter f) and purple (two RAFi bound, parameter g). Energies are normalized by the factor 1/RT, where R is Gas constant and T is the temperature. The diagram shows the specific situation of $\phi = 1$, where only reaction product stability is modulated. (D) PySB code to define the rules and energy-patterns that describe the diagram in (B). (E) Table of context dependent forward and reverse reaction rates. k is the binding rate, kr is the unbinding rate, with corresponding PySB rule indicated as subscript. (F) Model simulations for different values of K_{RR} with f = 0.001 and g = 1,000.

cells appears to originate not in the noise of intracellular reactions, but instead in the spatially restricted release of growth factors acting in an autocrine and paracrine manner (Gerosa *et al*, 2020). In the absence of better understanding of these extracellular processes, they are difficult to represent computationally. Moreover, calibration of stochastic models is substantially more difficult than for deterministic models (Fröhlich *et al*, 2016).

Fortunately, experiments showed that addition of any of several different exogenous growth factors to RAFi- or MEKi-adapted cells generates synchronous ERK pulses having the same dynamics and drug sensitivities as asynchronous pulses arising spontaneously

(Gerosa *et al*, 2020). Because single cells are much more similar to each other during ligand-induced than spontaneous pulsing, induced pulses are more amenable to characterization using standard transcriptional profiling and protein mass spectrometry methods. A further advantage is that synchronous pulses can be modeled at the population level by an ODE-model that is a reasonable simulacrum of single cell biology. In the current work, we used data from pulses generated by growth factors to provide insight into spontaneous pulses; consequently, we focused only on mechanisms downstream of receptor activation. Future work will be required to understand the origins and spatial distributions of ligands in the micro-environment of drug adapted cells undergoing asynchronous and spontaneous pulsing.

To further constrain MARM2.0, we used targeted proteomics with calibration peptides to measure the absolute abundances of two phospho-proteins (Fig EV1A) and all 11 protein species (Fig EV1B); data were collected at five vemurafenib concentrations yielding 55 data points for model calibration. In addition, we extracted relative abundances for three mRNA species (Fig EV1C) from genome-wide transcript profiling performed at eight vemurafenib concentrations and seven timepoints following EGF stimulation (yielding 45 calibration data points). Immunofluorescence imaging of pERK and pMEK provided the greatest amount of data (847 data points) and involved 234 different experimental conditions each having a different concentration of one or more of the following perturbations: EGF, RAFi, panRAFi or MEKi. Imaging data had single cell resolution, but population averages were used for model calibration, since we aimed to model the behavior of an average single cell. Training data were complimented with 2,209 immunofluorescence data points in 1,647 conditions for model validation (Dataset EV1), which are described in greater detail below.

Rule-Based modeling enables efficient calibration through Multi-Model optimization

To calibrate MARM2.0 on experimental data, we used gradient-based numerical optimization, which performs well for large models (Villaverde *et al*, 2019). Optimization is nonetheless challenging for a model with as many reactions as MARM2.0: weighted least squares minimization of an objective function required simulation for each of the 234 training conditions for every evaluation of the objective function, and this took minutes to perform. Optimization required hundreds of evaluations of the objective function and its derivatives, resulting in calibration runtimes on the order of weeks to months even on a cluster computer. However, we found that it was possible to exploit patterns in the perturbational data to substantially reduce the number of species in a condition-specific manner, thereby accelerating calibration (Fröhlich *et al*, 2019; Städter *et al*, 2021): in our calibration dataset, 122 conditions involved one perturbation (RAFi, panRAFi or MEKi individually), 111 conditions involved two perturbations (RAFi or MEKi followed by addition of EGF) and only one involved no perturbation, (Fig 1D, top). In the absence of a perturbing agent, all model species involving that agent (e.g., RAF bound to RAFi, Fig 1B) as well as a subset of downstream species (e.g., pEGFR activated by EGF) have zero concentrations and need not be modeled. To automatically generate, compile and track sub-models omitting zero concentration species for a diverse range of perturbations, we created routines that exploited the programmatic features of PySB (Lopez *et al*, 2013) and BNGL network generation (Blinov *et al*, 2004; see MultiModelFitting in Materials and Methods). This yielded models having up to 1.5 times fewer parameters than MARM2.0 itself (60–85 parameters compared to 91; Fig 1D, middle) and up to 68-fold fewer species (113–1,612 species compared to 7,774; Fig 1D, bottom). Multi-model objective calibration was performed using pyPESTO (a python reimplementation of the Parameter Estimation Toolbox; Stapor *et al*, 2018) allowing consistent generation of a full model based on calibration of sub-models; this is an exact approach that does not reduce the accuracy of the objective function or gradient evaluation. Overall, we found that using PySB to match model structure to data structure reduced median gradient evaluation time ~33-fold (from 46 to 1.4 h on a single compute core; Fig 1E), which for MARM2.0 extrapolated to a reduction of ~24 weeks in real time and ~450 years in CPU time (assuming 10^3 cores with a computational budge of 5 days each). Since multiple rounds of model refinement and calibration were necessary over the course of the current work, a 33-fold improvement in calibration time had a major impact. We expect that multi-model objective calibration will be broadly useful with other models involving perturbational datasets.

Following calibration, MARM2.0 quantitatively captured the effects of RAFi and MEKi treatment on baseline pERK levels in the drug adapted state and during transient EGF stimulation. Relatively few parameters converged on unique values (Fig EV2) due to the known non-identifiability of biochemical models having explicit forward and back reactions (Gutenkunst *et al*, 2007) as well as incomplete convergence of the optimizer due to limitations in the computational budget. We therefore used parameter sets from the 5% of optimization runs having the lowest value of the objective function (50 parameter sets) to generate a set of dynamical trajectories that approximated the impact of parametric uncertainty on simulations. For a large fraction of data points (34.3%), we found that 80% of simulated trajectories fell within experimental error bounds (Figs 2 and EV1), demonstrating good agreement between the calibrated model with experimental data. This does not constitute a rigorous quantification of parameter uncertainty (Fröhlich *et al*, 2014), but does account for correlation in parameter values (Eydgahi *et al*, 2013) and was the only practical approach given the number of parameters and species in MARM2.0.

Causal decomposition untangles intertwined BRAF^{V600E} and RAS driven signaling

When cells were adapted to RAFi (vemurafenib unless otherwise noted) for 24 h, steady-state pERK levels decreased with drug concentrations. In striking contrast, the amplitude of pERK pulses generated by adding exogenous EGF increased with RAFi concentration (Fig 2A, left). Thus, EGF (and other growth factors applied in a similar manner) induced pERK in proportion to the degree of BRAF^{V600E} inhibition. When MEKi (cobimetinib unless otherwise noted) was used over a dose range, a biphasic response was observed: below ~0.1 μM MEKi EGF-induced pERK levels increased with MEKi concentration but above ~0.1 μM MEKi they fell (Fig 2A, right). In all cases, the effects of EGF were transient and pERK levels returned to their drug adapted baseline levels within 1–2 h. The calibrated MARM2.0 model recapitulated all of these phenomena and we therefore sought a molecular explanation via model analysis.

 © 2023 The Authors

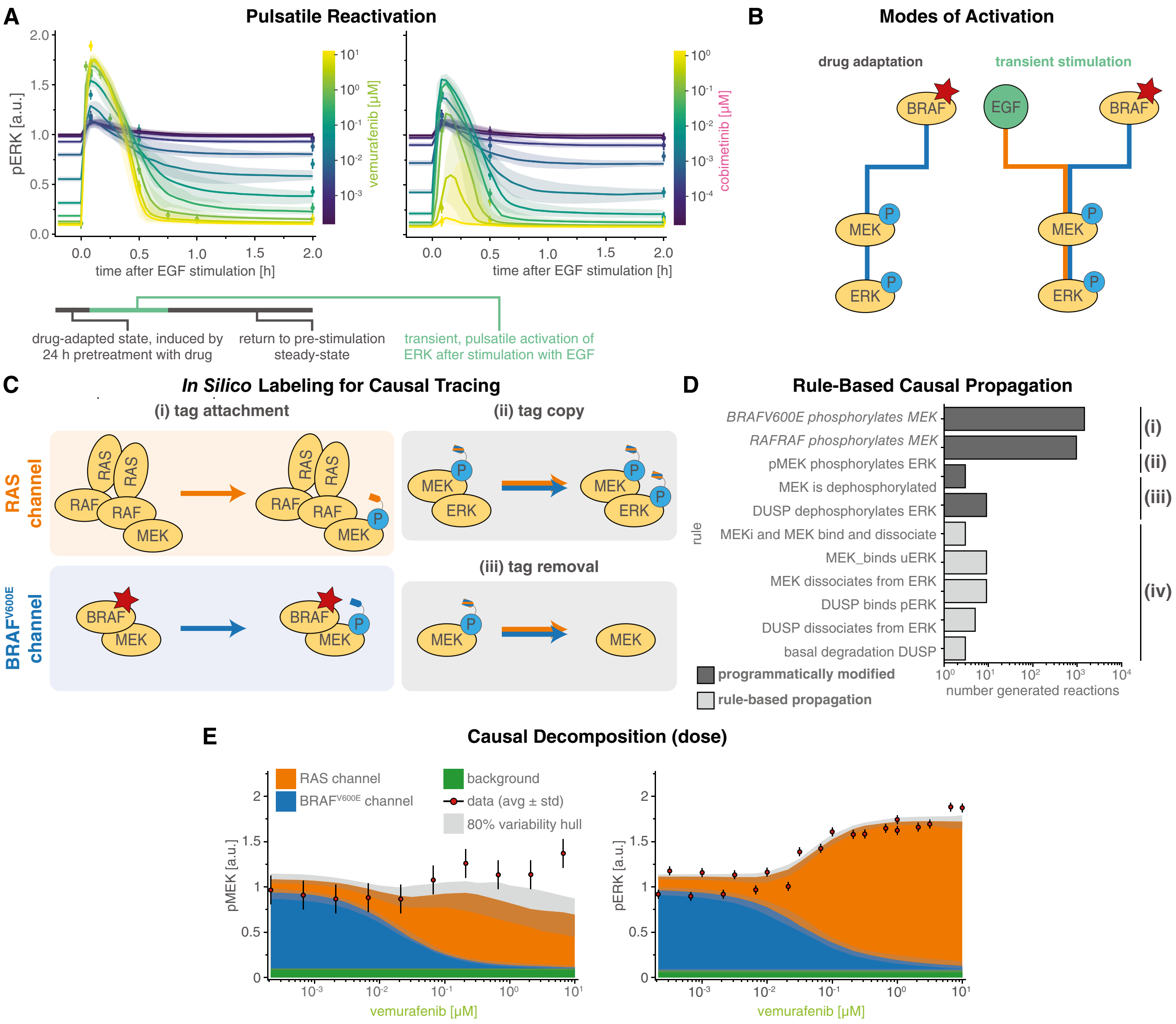

Figure 2. Causal decomposition of RAS and BRAF^{V600E} channels.

A Time course of pre- and post-stimulation pERK levels. Colors denote different concentrations of vemurafenib (RAFi) and cobimetinib (MEKi). Solid lines show median simulation values and shading indicates 80% percentiles over 50 best parameter sets.

B Toggling of reaction channels via their upstream activators BRAF^{V600E} (blue) or EGF (orange) during the two phases of pulsatile reactivation shown in (A): drug adaptation (left) and transient stimulation (right).

C Schematic for tracing of causal history using synthetic sites.

D, E (D) Rules affected by causal decomposition (E) Comparison of experimental data and decomposed model simulations (pMEK, pERK) at 5 min after EGF stimulation. Median (over 50 best parameter sets) simulations are shown as stacked areas with color indicating reaction channel (blue: BRAF^{V600E}, orange: RAS). Shading indicates 80% percentiles over 50 best parameter sets. Experiments were performed in A375 cell lines in 5% FBS medium following 24 h of drug adaptation. Cells were stimulated with EGF at a final concentration of 100 ng/ml. Data are shown as point-ranges with average over technical replicates (n = 2) as point and estimated standard deviation (over all datapoints) as a line. Data from different experiments (biological replicates) are shown separately.

Experimentally determined pMEK and pERK levels measure the sum of active MAPK kinases generated by oncogenic and chronically active BRAF^{V600E} and by transiently active EGFR (Fig 2B). To decompose these two sources of MAPK activity, we defined a "RAS reaction channel," which encompasses all reactions initiated by (RAS-GTP)$_2$-RAF$_2$ oligomers, and a "BRAF^{V600E} reaction channel" encompassing all MAPK reactions downstream of the BRAF oncogene. We use "reaction channel" in this sense to describe a set of proteins and protein–protein interactions that transduce a signal via post-translational modifications and/or formation of multi-protein assemblies. Because assemblies formed during immediate early signal transduction are typically transient (due to relatively low affinities), and modifications are reversible, a single protein species can participate in multiple reaction channels, but any

specific molecule is assumed to be part of a single channel at specific point in time. In principle, a signaling network may be decomposable into reaction channels based on a variety of criteria and we chose the most obvious one: the origin of the signal (i.e., the most upstream activating event). In MARM2.0, this is constitutively active $BRAF^{V600E}$ for the $BRAF^{V600E}$ reaction channel and ligand bound RTKs (represented in our models by EGFR) for the RAS reaction channel. We then tracked individual MEK and ERK phosphorylation events based on whether they could be traced back to $BRAF^{V600E}$ or RAS-GTP. These definition of reaction channels is related to the known dichotomy between RAFi-sensitive monomeric and RAFi-resistant dimeric RAF signaling (Baljuls *et al*, 2013); however, since MARM2.0 allows $BRAF^{V600E}$ dimerization and binding to RAS-GTP, but requires formation of complete (RAS-GTP)$_2$-RAF$_2$ dimers for activation by RAS, MEK phosphorylation by oncogene-containing dimers ($BRAF^{V600E}{}_2$, $BRAF^{V600E}$-CRAF, (RAS-GTP)-$BRAF^{V600E}{}_2$ and (RAS-GTP)-$BRAF^{V600E}$-CRAF) is attributed to the $BRAF^{V600E}$ channel. Only phosphorylation by (RAS-GTP)$_2$-RAF$_2$ dimers, the normal physiological tetramer, is attributed to the RAS channel. Thus, our formulation of reaction channels recapitulates the causal dependency of ERK activity on upstream signaling events rather than precisely subdividing the system based on postulated resistance mechanisms.

In agent-based modeling, it is straightforward to keep track of the upstream origins of a single molecule or event and thereby generate causal traces or "stories" (Boutillier *et al*, 2018), but ODE models only describe the properties of ensembles of molecules. To perform *causal decomposition*, i.e., to apply causal tracing of reaction channels to ODE models, we applied an *in silico* labeling strategy that involved adding a virtual "tag" to pMEK (Fig 2C, Materials and Methods Section Causal Signal Decomposition) at the time of its generation by (RAS-GTP)$_2$-RAF$_2$ (orange, top left panel) or $BRAF^{V600E}$ (blue, bottom left panel). The tag was copied from pMEK to pERK upon ERK activation (blue/orange, top right panel) and removed during dephosphorylation (blue/orange, bottom right panel). Implementing this approach required modification of only of a few PySB rules (Fig 2D) and did not change model dynamics.

For causal decomposition of MARM2.0 under a range of conditions, computational labeling of both pMEK and pERK was necessary, since the two active forms do not have the same proportionality (degree of amplification) in the two reaction channels: in the $BRAF^{V600E}$ channel, the MEK phosphorylation rate is lower when MEKi is bound to uMEK, generating a higher ratio of *apo*-pMEK to pMEK-MEKi than in the RAS channel, in which the MEK phosphorylation rate is independent of MEKi binding. The origins of this phenomenon are described in greater detail below. Since MEKi inhibits the catalytic activity of pMEK, amplification from pMEK to pERK is higher in the $BRAF^{V600E}$ than the RAS channel.

The value of causal decomposition was illustrated when we investigated the experimentally observed pMEK levels that remained roughly constant over a 10^5-fold range of RAFi concentrations (as monitored at the 5-min peak of an EGF-induced pulse, Fig 2E left). Causal decomposition showed that this unexpected behavior arose from a steady reduction in the activity of the $BRAF^{V600E}$ channel (blue) with increasing RAFi and a simultaneous and offsetting increase in signaling in the RAS channel (orange). This was true of all three RAFi and five MEKi tested (Fig EV3) and represents a classic case of pathway rewiring that is obscured at the level of total MAPK activity.

Slow transcriptional feedbacks imprint drug adapted state and unravel cyclic causal dependencies

Experimental data (Pratilas *et al*, 2009; Lito *et al*, 2012; Gerosa *et al*, 2020) and model trajectories show that DUSP (blue), SPRY (orange), and EGFR (green) proteins (dark colors) and mRNA (light colors) levels are substantially lower in cells adapted to RAFi for 24 h when compared with drug-naïve cells (Figs 3A left and EV2B and F). This is consistent with the known role of MAPK activity in promoting the expression of negative (feedback) regulators. However, it raises the question: why is pERK only transiently activated by EGF in drug adapted cells if feedback is suppressed? When we simulated the induction of ERK pulses by exogenous EGF in drug adapted cells, we observed modest increases in EGFR, DUSP and SPRY mRNA levels (Fig 3A right), consistent with respective experimental training data (Fig EV1C). However, at the protein level DUSP and SPRY remained almost constant and EGFR decreased. We surmised that this reflected the operation of transcriptional feedback on a longer timescale (> 2 h) than a typical EGF-mediated pulse (30–90 min). Thus, the transience of ERK activation is not a consequence of negative feedback at the level of the MAPK pathway.

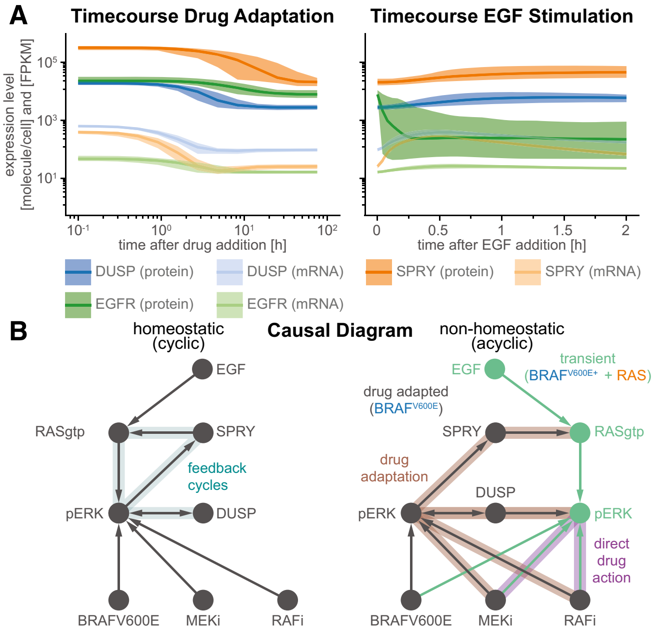

Figure 3. Transcriptional feedback imprints a sparse drug adapted state.

A Simulated time courses of pre- (left) and post-stimulation (right) protein (dark colors) and mRNA (light colors) expression levels of genes that are subject to transcriptional control by pERK. Solid lines show median values, shading indicates variability across 80% of 50 best parameter sets.

B Schematic of the structural causal model for the effect of RAFi and MEKi on pERK under homeostatic (left) and non-homeostatic (right) conditions. Drug adapted signaling elements are in gray, transient signaling elements are in cyan. Feedback cycles have blue shading (left only). Drug adaptation effects have brown shading and direct drug action has purple shading (right only).

 © 2023 The Authors

Instead, model analysis showed that changes in EGFR protein levels were a consequence of receptor endocytosis, and degradation. Thus, it is EGFR trafficking that controls the duration of a pERK pulse in drug adapted cells, consistent with existing models of EGFR (Starbuck & Lauffenburger, 1992; preprint: Dessauges *et al*, 2021) and other transmembrane receptors (Becker *et al*, 2010). However, on the longer timescale of drug adaptation, transcriptional feedback is the primary determinant of pERK levels. Similar separations in timescale have been previously observed in other aspects of EGFR and MAPK signaling. For example, individual kinase phosho-states turn over on timescale of seconds but measurable changes in MAPK activity are a least hundred-fold slower, requiring minutes to hours (Kholodenko *et al*, 1999; Kleiman *et al*, 2011; Reddy *et al*, 2016). Thus, slow population average responses mask underlying biochemical reactions happening on faster timescales.

The presence of feedback loops in a network usually generates cycles in the causal diagram (preprint: Mooij *et al*, 2013; Fig 3B left), complicating model analysis (preprint: Pearl & Dechter, 2013; preprint: Spirtes, 2013). In the case of MARM2.0, a cycle involving positive regulation of feedback regulators by MAPK activities means, for example, that pERK activity could ultimately control DUSP levels or DUSP levels could control pERK activity. However, timescale separation makes it possible to generate an acyclic causal diagram for MARM2.0 (Hyttinen *et al*, 2012; Fig 3B right), in which the effects of RAFi and MEKi on pERK are split into the rapid and immediate effects of drug on kinase activity (*direct drug action*, purple shading) and a slower process involving changes in the levels of feedback proteins (*drug adaption*, brown shading). Prior to EGF stimulation, when only the BRAF^{V600E} channel is active (Fig 2B left), MEKi and/or RAFi levels control pERK levels in drug adapted cells (*drug adapted pERK*; gray in Fig 3B), which in turn determine DUSP and SPRY concentration and, thus, the strength of negative feedback on pERK in the RAS channel (*transient pERK*, turquoise in Fig 3B). The indeterminacy between drug adapted pERK and DUSP levels remains (illustrated by a bidirectional edge in the graph), but this does not affect the determinacy between drug adapted DUSP and transient pERK levels. Thus, timescale separation during drug adaption facilitates the establishment of a cell state that has distinct "rewired" signal transduction properties and is not altered by a single pERK pulse. Moreover, this timescale separation enables us to causally attribute these changes in signal transduction properties to distinct drug effects.

MAPK signaling is rewired by drug adaptation and direct inhibition

The ratio of output to input signals in a network (the gain) is a fundamental property of a signal transduction system that can be used quantify rewiring. Gain often varies along a series of reactions in a single channel—for example, the number molecules of pERK generated per molecule of RAS-GTP when stimulated with EGF ligand. Gain could in principle be quantified by sensitivity (Goldbeter & Koshland, 1981), but as a mathematical concept, sensitivity is defined at steady-state, whereas signaling in the RAS channel is transient. Sensitivity could also be computed pointwise at every time point (Chen *et al*, 2009), but this would not account for the fact that input and output signals for any specific step in a network often have different timescales. For example, modeling revealed conditions in which an input signal (e.g., pEGFR levels) had started to fall following EGF stimulation, while a downstream event (e.g., formation of active RAS-GTP) was still increasing. We therefore defined the gain of a reaction channel as the ratio of L_∞ or L_1 norms (with respect to a logarithmic timescale) between input and output signals in corresponding model trajectories (see Materials and Methods; Signaling Gain). The L_1 norm quantifies the area under the curve of the signal, whereas the L_∞ norm quantifies the height of the peak of the signal. Both represent scalar, time-independent quantities. For simplicity, we normalized gain to equal 1 in the absence of inhibition.

Gain for each of the two MAPK reaction channels was investigated graphically using a formalism in which each node represents a "signal" that is defined as the sum of active model species, and edges represent signaling steps that are defined as the action of one or more PySB reaction rules. Gain was computed along each edge of the graph by computing the ratio of norms of input and output nodes. The graph in Fig 4A has been arranged so that each signaling step (edge) is affected by as few drug actions as possible—ideally only one—allowing changes in gain to be attributed to direct drug action (purple) or drug adaptation (brown). The graph contains three steps for the RAS channel (orange; steps R1-R3) and two steps for the BRAF^{V600E} channel (blue; steps B2-B3) with the channels "aligned" at the third step (pMEK phosphorylation of ERK; Fig 4A). We then used the calibrated model to compute time-resolved signals for all nodes at multiple drug concentrations (Fig 4B) and determined the gain (Fig 4C). To visually summarize the inhibitor concentration-dependent states of the graph, we generated separate representations for RAFi (Fig 4D) and MEKi (Fig 4E), with signal activity indicated as node opacity and gain as edge opacity.

We found that adaptation to RAFi and MEKi had a similar impact on the first step (R1) for both drugs (Fig 4C, top panels). At low to medium drug concentrations (RAFi: ~10^{-4} to10^{-2} μM, MEKi ~10^{-5} to 10^{-3} μM), the gain from pEGFR to RAS-GTP was close to zero, representing almost complete inhibition of EGF-mediated signaling by the combined actions of feedback regulators such as SPRY. At medium to high drug concentrations (RAFi: ~10^{-2} to 10^{-1} μM, MEKi: ~10^{-3} to 10^{-0} μM) a reduction in the levels of feedback regulators led to a relief of feedback and an increase in gain. Moreover, the transcriptional control of EGFR expression by MAPK activity resulted in decreasing input activity (pEGFR, top left Fig 4B) at higher drug concentrations (decreasing pEGFR opacity, Fig 4D and E) of both drugs. At the second step, for medium to high RAFi and MEKi concentrations, we found that B2 had gain close to zero, but R2 gain was close to one (Fig 4C, middle panels), indicating channel-specific effects for both drugs. For RAFi, we attributed this channel specificity to difference in the affinity of the RAFi for monomeric RAF in the BRAF^{V600E} channel and dimeric RAF in the RAS channel (orange vs. blue colored nodes). The difference in affinity is determined by the thermodynamic parameter $\Delta\Delta G_{dim}$ (Box 3), which encodes the ratio of drug affinities for the first and second protomers of a RAF dimer; for vemurafenib this difference was estimated to be ~600-fold (median of values from best 5% of fits). Thus, even at 10 μM, the highest vemurafenib concentration tested, ~75% of RAF dimers had one protomer not bound to drug (Fig 4F, left), a configuration that is active as a kinase (Karoulia *et al*, 2017). Estimated ranges for $\Delta\Delta G_{dim}$ were similar for the two other type I½ RAFi drugs we tested (dabrafenib and PLX8394; Figs EV2 and

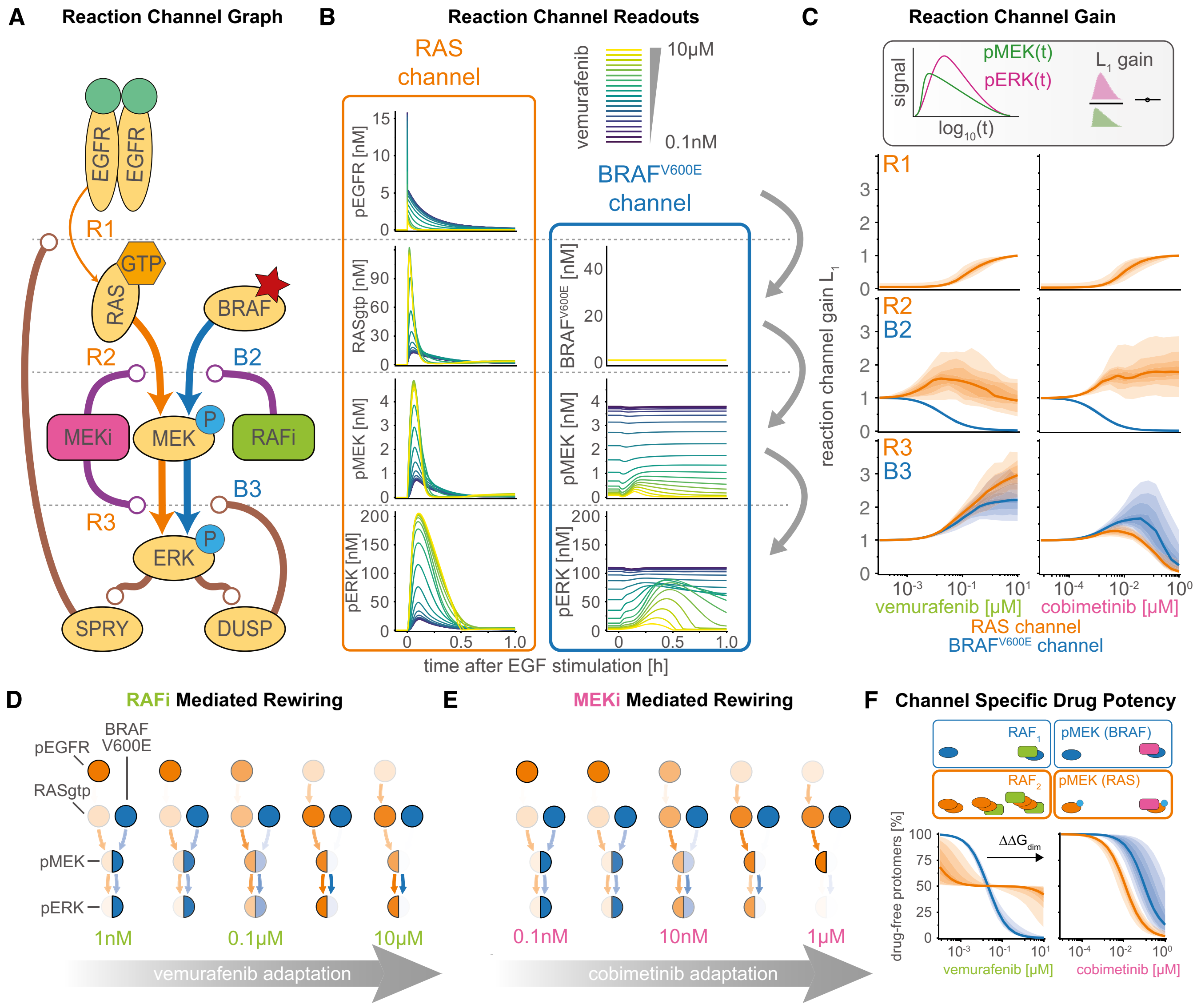

Figure 4. Quantification of signal transduction in RAS and BRAF^{V600E} channels.

A Simplified network model depicting intertwined RAS and BRAF^{V600E} channels and feedbacks. Nodes in the network correspond to key steps in signal transduction in each of the reaction channels. Lines ending with arrowheads indicate signaling flow along multiple reaction steps and lines with ending with circles indicate direct drug action (purple) and drug adaptation (brown) as introduced in Fig 3B.

B Decomposition of RAS and BRAF^{V600E} channel signal activity at the different nodes of the simplified network from (A) for different concentrations of vemurafenib. Color indicates vemurafenib concentration. Simulations were performed for a single, representative parameter vector.

C Quantification of signal transmissions in terms of gain along the edges of the simplified network in (A) (top, R1; middle R2/B2; bottom R3/B3) for different concentrations of vemurafenib (left) and cobimetinib (right). Color indicates the reaction channel. Solid lines show median values, and shading indicates 20, 40 60 and 80% percentiles over 50 best parameter sets.

D, E Visualization of pathway rewiring as a result of drug adaptation in the simplified network in (A). The opacity of nodes denotes the median normalized signaling activity (shown in B); the opacity of arrows denotes median normalized gain (shown in C), where 100% corresponds to a signaling gain of 2.

F Quantification of efficacy of drug inhibition for RAF monomers (blue) and RAF dimers (orange). For RAF dimers, each protomer is counted individually. Solid lines show median values, and shading indicates 20, 40, 60 and 80% percentiles over 50 best parameter sets. Experiments were performed in CRISPRa-EGFR A375 cell lines in 5% FBS medium following 24 h of drug adaptation. Cells were stimulated with EGF at a final concentration of 100 ng/ml. Single drug response data are shown as point-ranges with average over technical replicates (n = 2) as point and estimated standard deviation (over all datapoints) as line. Data from different experiments (biological replicates) are shown separately.

EV4C). For MEKi, we attributed the channel specific potency in the second step to a decrease in MEK phosphorylation rate by BRAF^{V600E} for BRAF-uMEK-MEKi complexes when compared with BRAF-uMEK complexes; modeling suggested a ~6.9×10^3-fold decrease in phosphorylation rate for BRAF-uMEK-cobimetinib when compared with apo BRAF-uMEK. Estimated values were similar (> 3,000 fold) for trametinib, but substantially lower (< 500 fold) for binimetinib, PD0325901 and selumetinib, consistent with

    © 2023 The Authors

previously reported differences in the activity of these drugs (Pino *et al*, 2021). In all cases, the combination of lower RAFi affinity or lower MEKi-dependent phosphorylation rate resulted in incomplete inhibition of pMEK in the RAS channel (Fig EV3).

In the third step, we found that gain from pMEK to pERK (B3 and R3) increased at medium to high concentrations of RAFi (Fig 4C, bottom left panel), due to a reduction in DUSP expression levels. In contrast, MEKi did not have any effect on B3/R3 gain at medium concentrations (~10^{-3} to 10^{-2} μM; Fig 4C, bottom right panel). This was unexpected, since the analysis described above shows that DUSP levels are controlled by drug adapted pERK levels, which are lower at medium concentrations of MEKi and RAFi (blue, middle panels, Fig 4C). However, B3/R3 are the only steps in which the model implements two distinct effects for MEKi: increases in ERK activity as a result of drug adaptation, i.e., DUSP downregulation, (brown, Fig 4A) and reductions in ERK activity via direct drug action on MEK (purple, Fig 4A). Modeling suggested that direct drug action and adaptation balanced each other at intermediate MEKi concentrations and direct inhibition became dominant only at high concentrations. We observed few channel specific effects when comparing R3 to B3, suggesting that neither small differences in the *apo*-pMEK to MEKi-pMEK ratio (Figs 4F, right and EV4D) nor in MEKi affinity for uMEK when compared with pMEK (Fig EV4F) resulted in substantial channel-specific differences in MEKi potency in the third step (see Materials and Methods Section on Causal Decomposition for a detailed explanation of both mechanisms). Thus, we concluded that the ~100-fold shift in MEKi potency for pERK activated by EGFR when compared with $BRAF^{V600E}$ activated pERK (Figs 2A and EV3A) primarily arises in the second step as a result of a lower rate of phosphorylation rate of MEKi-uMEK by $BRAF^{V600E}$ compared with apo uMEK.

Additionally, we found that, at high concentrations of RAFi (Fig 4D, rightmost diagram), step B3 (blue) had high gain (due to low DUSP activity) under conditions in which the channel transmitted no signal and was functionally inactive (due to RAFi-$BRAF^{V600E}$ binding). This "signaling primed" configuration implies that the anti-proliferative effects of RAFi are highly sensitive to anything able to activate MEK directly, such as a mutation in the kinase. Consistent with this, activating mutations such as $MEK1^{C121S}$ are observed to give rise to acquired drug resistance in patients (Wagle *et al*, 2011). A directly analogous state of high gain but low activity is observed in the RAS channel in adpated cells and potentiates the mitogenic effects of ligand-mediated RTK activation and of RAS mutation (e.g., $NRAS^{Q61K}$ discussed below). It is possible that identifying signaling steps with low activity but high gain may be generally useful in pinpointing mechanisms involved in acquired drug resistance.

Pulsatile signaling induces apparent drug interactions

MEK and RAF inhibitors are normally used in combination. To study drug interaction and also test the predictive power of MARM2.0 in conditions distinct from those used for model training, we simulated the effects of RAFi plus MEKi combinations on pERK levels with a model trained on single-drug responses alone (the model training described above). Drug dose–response relationships were then visualized as surface plots (Fig 5A) and isobolograms (Fig 5B). In the absence of stimulation with exogenous growth factors (Fig 5A (i)), we predicted a monotonic decrease in pERK levels with increasing doses of both drugs (left panels) and experimental data were in agreement (right panels). In BRAFi-adapted and EGF stimulated cells, we predicted a more complex landscape (Fig 5A (ii)), in which pERK was relatively drug resistant along a L-shaped region (red dashed outline) at intermediate MEKi and high RAFi concentrations with a gradual decrease at high MEKi concentrations. Using isobolograms, we observed disconnected level sets (bottom, Fig 5B), recapitulating the non-monotonic pERK response to MEKi 5 min after EGF stimulation in Fig 2A, in which peak pERK levels first rose and then fell with increasing drug concentration. Experimental data (right panel, Fig 5A (ii)) were qualitatively similar to predictions (left panel) and differences were primarily in the magnitude of pERK, not the shape of the response surface (bottom, Fig 5B). Disconnected isobolograms (bottom, Fig 5B) are noteworthy, because measures of drug interactions such as Loewe additivity (Loewe, 1928) or the Chou–Talalay combination index (Chou *et al*, 1993) require a one-to-one mapping between dose and response (a bijective curve) and cannot be applied in this context. However, comparing pERK levels to null models for Bliss independence (Bliss, 1939; Bliss, Fig 5C) and highest single agent (Lehár *et al*, 2007; HSA, Fig 5D) revealed negligible drug interaction (white) in the absence of EGF (top panels) in simulation (left) and experimental data (right). Under conditions of EGF stimulation (bottom panels), we observed substantial discordance between the magnitude and sign of drug interaction as scored by Bliss criteria (Fig 5C) and HSA (Fig 5D). Thus, existing definitions of drug synergy and antagonism do not adequately describe the complex dose–response landscapes we observed.

When we decomposed dose–response surfaces for EGF-stimulated conditions (left, Fig 5E) into $BRAF^{V600E}$ (middle) and RAS channels (right), we observed little RAFi and MEKi interaction in the $BRAF^{V600E}$ channel (left, Fig 5F) and either strong synergy (blue) or strong antagonism (red) in the RAS channel depending on drug concentration (right). When we computed gain in the RAS channel for R1, R2 and R3 (Fig 4A) at different drug concentrations, we observed low gain for R1 at RAFi and MEKi concentrations below 10 and 1 nM respectively (first panel, Fig 5G), gain close to 1 for R2 at all concentrations (second panel) and low gain for R3 at MEKi at > 1 μM and high gain at RAFi at > 0.1 μM (third panel). When the total gain for steps R1–R3 was computed as pointwise multiplication of the three surfaces, the L-shaped region of drug resistant pERK (fourth panel) was regenerated (Fig 5A (ii)). Thus, the overall drug response landscape can be explained by the superposition of adaptive drug response on R1 (brown, first panel), and direct drug effects on R3 (purple, third panel).

Sustained signaling does not induce drug interaction

To study the effects of RAFi and MEKi on signaling in the RAS channel under conditions of sustained rather than transient EGFR activation, we over-expressed EGFR using CRISPRa (Gerosa *et al*, 2020), yielding two cell lines with 4-fold (light blue) and 9-fold (turquoise, referred to as A375 CRISPRa-EGFR below) increases in expression levels (Fig 6A). It has previously been shown that, when EGFR is overexpressed to this degree, mechanisms of receptor endocytosis and degradation are saturated and EGFR becomes chronically rather than transiently active in the presence of ligand (Wiley, 1988; Lund

© 2023 The Authors

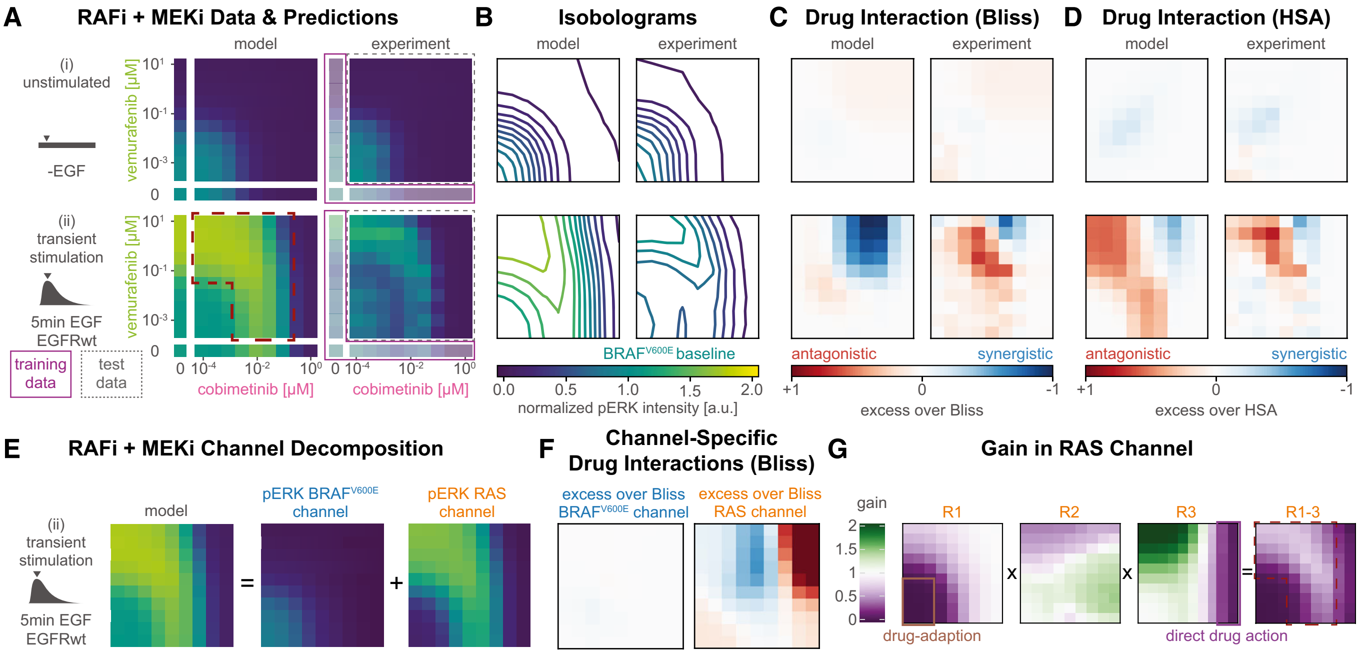

Figure 5. Prediction and analysis of drug combinations.

Experiments were performed in A375 cell lines in 5% FBS medium following 24 h of drug adaptation. Cells were stimulated with EGF at a final concentration of 100 ng/ml. Data are shown as average over technical replicates ($n = 2$).

- A Experimental data and model simulations (median over 50 best parameter sets) for pERK (color) combination response without EGF stimulation (top) and 5 min after EGF stimulation (bottom). Training data have lower opacity and purple outline. Test data have a gray, dashed outline.
- B Isobolograms of smoothed dose response surfaces from (A) (5 min after EGF addition). Concentrations and color scheme are the same as in (A). Smoothing was performed using a Gaussian filter with 0.75 as standard deviation in $\log_{10}$-concentration units.
- C, D Analysis of drug synergy according to excess over Bliss (C) and highest single agent (HSA; D). Concentrations are the same as in (A).
- E Decomposition of pERK model simulations at 5 min after EGF stimulation (left) in $BRAF^{V600E}$ (middle) and RAS (right) channels. Color and concentrations are the same as in (A).
- F Drug interaction analysis for decomposed channels. Concentrations are the same as in panel (A). Colors are the same as in panel (C). Drug effects were not normalized to baseline condition (see Materials and Methods).
- G Quantification of signaling gain in the RAS reaction channel. Pointwise multiplication is indicated by *x*. Purple and brown outlines indicate drug effect responsible for lower gain. Concentrations are the same as in (A).

et al, 1990; Kiyatkin *et al*, 2020). Consistent with this, we found that, upon ligand addition, pERK levels in RAFi-adapted CRISPRa-EGFR cells rose rapidly to a peak at ~30 min and then fell slightly to level at roughly ~75% of their levels in the absence of RAFi exposure; pERK remained at this level for at least 24 h in both experiments and simulations. Under these conditions, RAFi had substantially lower efficacy (EC_{max}; Fig 6B) and MEKi had lower potency (EC_{50}; Fig 6C) than in cells not stimulated with EGF. Channel decomposition (Fig 6B right panels) revealed an increase in pMEK and pERK levels in the RAS channel (orange) as a result of sustained EGFR activity. Analysis of pERK phase space with DUSP and SPRY mRNA and protein levels showed similar distributions at 8 h post EGF-stimulation in drug adapted CRISPRa-EGFR cells and pre EGF-stimulation in drug adapted $EGFR^{wt}$ cells, suggesting that a steady state had been reached 8 h post EGF-stimulation (Fig EV5D). Thus, sustained activation of the RAS channel is a sufficient explanation for the relative resistance of EGFR amplified cells to RAFi and MEKi.

When we predicted the pERK dose–response surface for combined RAFi and MEKi treatment of CRISPRa-EGFR cells (8 h after stimulation with EGF) using single drug training data (Fig 6D left), we observed incomplete pERK inhibition at high RAFi and medium MEKi concentrations. The resulting isobolograms had a convex shape (Fig 6E). Moreover, we observed minimal drug interaction by Bliss (Fig 6F) or HSA criteria (Fig 6G). This differs from what was observed with pulsatile RTK activation (Fig 5C and D bottom panels) and suggests that drug interactions in the case of pulsatile signaling were only possible due to timescale separation between drug adaption and direct drug action.

Structure-Based model formulation enables generalization across inhibitor classes

In MARM2.0, the thermodynamic parameter $\Delta\Delta G_{dim}$ describes changes in the stability of $(RAFi\text{-}RAF)_2$ complexes; these have been studied in detail via crystallographic structures (Rukhlenko *et al*, 2018). Negative $\Delta\Delta G_{dim}$ values manifest themselves as a loss of drug affinity by the second protomer in a RAF dimer. It is well-established that this leads to lower RAFi efficacy in the RAS channel when compared with the $BRAF^{V600E}$ channel (Fig 4C and F). However, due to energy conservation (Box 3), $\Delta\Delta G_{dim} < 0$ also results in a higher dissociation rate of RAF_2 complexes at high RAFi

 © 2023 The Authors

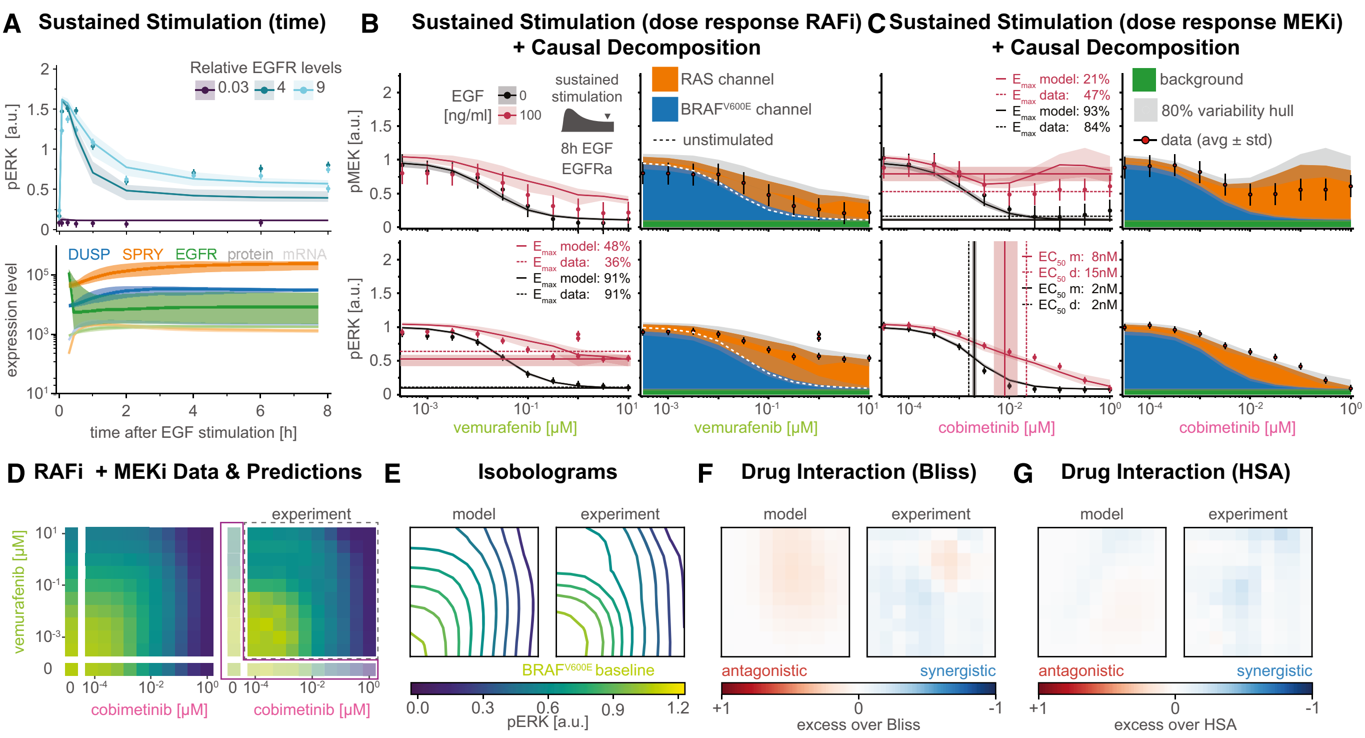

Figure 6. Drug resistance arising from EGFR upregulation.

A Simulation of time course data for three different clones (two overexpression, one knockdown). Solid lines show median values, shading indicates variability across 80% of 50 best parameter sets. Top plot shows pERK response. Bottom plot shows mRNA (light colors) and protein (dark color) expression level changes.

B, C Simulation of pERK (top) and pMEK (bottom) dose response data with and without EGF (at 8 h after stimulation) in response to vemurafenib (B), cobimetinib (C). Left panels show EGF stimulated (red) and unstimulated (black) conditions. Right panels show decomposed model simulations for EGF stimulated conditions as colored areas (blue: $BRAF^{V600E}$ channel, orange: RAS channel) and for unstimulated conditions as white dashed lines. Thick lines or stacked areas show median simulation values and shading indicates 80% percentiles over 50 best parameter sets. Thin vertical lines denote EC_{50} values, horizontal lines denote E_{max} values (data: dashed, model: solid).

D Experimental data (right) and model predictions (left, median over 50 best parameters) for pERK (color) in response to vemurafenib plus cobimetinib at 8 h after EGF stimulation. Training data have lower opacity and purple outline. Test data have a gray, dashed outline.

E Isobolograms of smoothed dose response surfaces from (A). Smoothing was performed using a Gaussian filter with 0.75 as standard deviation in log10-concentration units.

F, G Analysis of drug synergy according to excess over Bliss (F) and HSA (G).

concentrations (Fig EV4E). Thus, thermodynamically formulated models are ideal for describing the phenotypic effects of different kinase inhibitors based on their allosteric properties.

In contrast to type I½ RAF inhibitors, type II inhibitors (also called panRAFi; Box 2) such as LY3009120 and AZ-628 (Henry *et al*, 2015; Noeparast *et al*, 2018) inhibit both monomeric RAF in the $BRAF^{V600E}$ and dimeric RAF in the RAS channel with similar affinity. Crystallographic data suggest that this arises because panRAF inhibitors do not destabilize $(RAFi\text{-}RAF)_2$ complexes, i.e., they do not induce allosteric changes. To determine whether MARM2.0 correctly predicts response to type II inhibitors based on the absence of allostery, we calibrated MARM2.0 using data from A375 cells that were treated with LY3009120 (Fig 7A) or AZ-628 for 24 h (Fig EV5A), but not stimulated with EGF. This allowed estimation of drug affinity for monomeric RAF (ΔG); $\Delta\Delta G_{dim}$ was fixed to 0 to reflect loss of allostery. We then generated predictions for pMEK (top) and pERK (bottom) levels 8 h after EGF stimulation (red) in cells adapted to LY3009120 (Fig 7A left panels). Predictions matched experimental data under the same conditions and causal decomposition confirmed that RAF was strongly inhibited in the RAS channel (right panels). We also observed good agreement between model predictions and experimental data for LY3009120 in combination with cobimetenib in EGF-stimulated, drug adapted cells (Fig 7B). Analysis of drug interactions using HSA and Bliss criteria (Fig 7C) revealed a similar level of additivity (but little or no synergy) in model predictions and experimental data (note that the isoboles are curved not due to synergy but our use of logarithmic concentration axes). These data show that MARM2.0 can correctly predict the properties of different RAF inhibitors based on differences in their allosteric properties alone.

Successes and limitations in extending MARM2.0 to other resistance mechanisms

$NRAS^{Q61K}$ is a frequently observed resistance mutations found in melanoma patients treated with RAF/MEK therapy (Long *et al*, 2014; Shi *et al*, 2014). We modeled $NRAS^{Q61K}$ as RTK-independent activation of the RAS channel (Burd *et al*, 2014), with

© 2023 The Authors

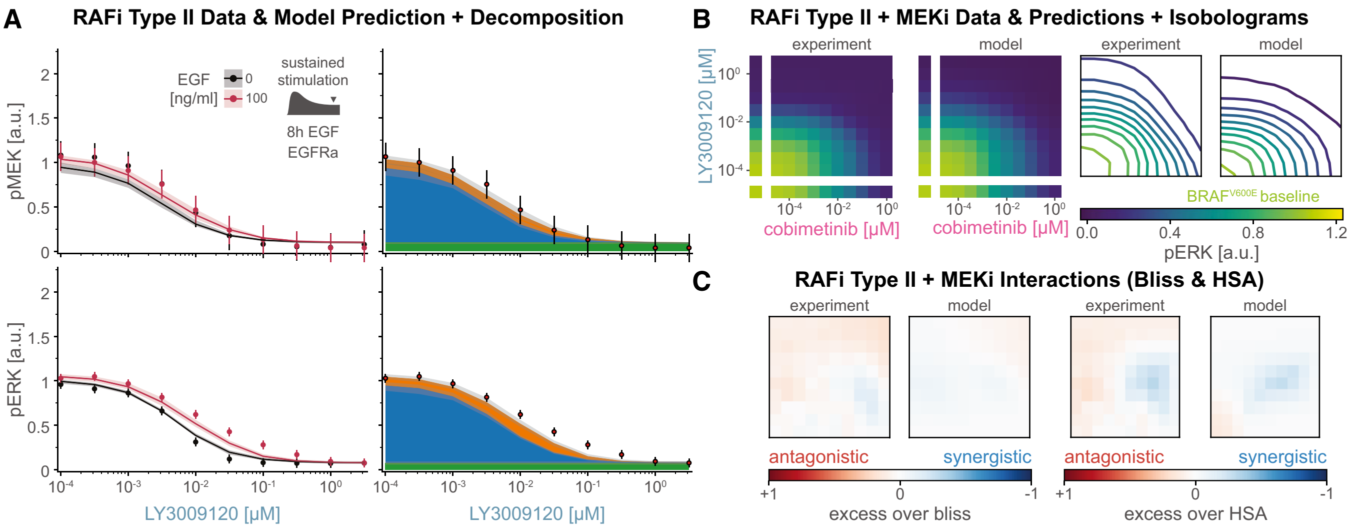

Figure 7. Prediction of response to panRAF inhibitor LY3009120.

Experiments were performed in CRISPRa-EGFR A375 cell lines in 5% FBS medium after 24 h of drug adaptation. Cells were stimulated with EGF at a final concentration of 100 ng/ml.

A Comparison of pMEK (top) and pERK (bottom) dose response predictions and experimental validation with and without 8 h of EGF stimulation. Left panels show EGF stimulated (red) and unstimulated (black) conditions. Right panels show decomposed model simulations for EGF stimulated conditions as colored areas (blue: $BRAF^{V600E}$ channel, orange: RAS channel) and for unstimulated conditions as white dashed lines. Thick lines or stacked areas show median simulation values and shading indicates 80% percentiles over 50 best parameter sets. Data are shown as point-ranges with average over technical replicates (n = 2) as point and estimated standard deviation (over all data points) as line.

B Experimental data (left panels) and predicted (right panels) pERK (color) for LY3009120 plus cobimetinib 8 h after EGF stimulation shown as heatmap (left panel group) and smoothed isobolograms (right panel group). Model simulations represent median values over 50 best parameter sets. Smoothing was performed using a Gaussian filter with 0.75 as standard deviation in log10-concentrations.

C Analysis of drug synergy according to excess over Bliss (left two panels) and HSA (right two panels) for data (left and model simulation (right) shown in (B).

baseline pERK levels inferred from drug-naïve $NRAS^{Q61K}$ $BRAF^{V600E}$ double mutant melanoma cells (Fig 8A). Under these conditions, simulations recapitulated higher baseline pERK and predicted 9-fold lower efficacy for RAFi ($NRAS^{Q61K}$, turquoise; left panels) and 21-fold lower potency for MEKi (Fig 8B, right panels) when compared with NRAS wild-type cells ($NRAS^{wt}$, purple). These predictions were confirmed in A375 cells engineered to conditionally express $NRAS^{Q61K}$ (Yao *et al*, 2015), but the observed loss of MEKi potency was even greater than modeling predicted (32-fold). Causal decomposition of (modeled) pERK activity in the presence of drug combinations (varying MEKi plus 1 μM RAF; Fig 8C) showed that 1 μM RAFi was sufficient to completely block activity in the $BRAF^{V600E}$ channel (blue) without affecting the RAS channel (Fig 8B and C). This made it possible to study $NRAS^{Q61K}$ signaling without interference from the $BRAF^{V600E}$ oncogene.

Based on this insight, we devised a triple combination experiment to study drug interactions between panRAFi and MEKi in the RAS channel alone (Fig 8D, top left panel). A375 $BRAF^{V600E}$ $NRAS^{Q61K}$ cells were grown in the presence of 1 μM vemurafenib plus different concentrations of LY3009120 and cobimetinib for 24 h and pERK levels then determined (top right panel). In contrast to the analogous experiment without 1 μM vemurafenib (Fig 7C), we observed pronounced synergy (blue) at low to medium concentrations of both inhibitors (~1–100 nM) by Bliss (bottom left panel) and HSA criteria (bottom right panel). Similar synergy has previously been observed in KRAS-driven cell lines of diverse origins (Yen *et al*, 2018). However, we found that the effects of combining three drugs in double mutant A375 cells were not accurately predicted by MARM2.0 (Fig EV5D). We hypothesized that drug synergy is likely to arise due to a combined allosteric effect of both drugs on RAS–RAF–MEK complexes, as similar interactions have been described for combined treatment of MEKi and APS-2-79, a type II inhibitor of the KSR scaffolding protein (Box 2; Dhawan *et al*, 2016). MARM2.0 does not include such allosteric effects and was not trained on combination data that would be necessary to infer the strength of the combined effect *a posteriori*. This limitation of MARM2.0 can be rectified in future studies, but serves to reveal how the subtleties of drug interactions can be relatively difficult to discern when multiple parallel reaction channels are active.

Model for melanoma cell line generalizes to colorectal cell line

$BRAF^{V600E}$ mutations are found in a variety of cancers other than melanoma, notably colorectal cancers. To investigate whether MARM2.0 could predict the responses of $BRAF^{V600E}$ colorectal cancers to RAFi, we collected data from HT29 cells, which carry a heterozygous $BRAF^{V600E}$ mutation and have high EGFR expression (similar to A375 EGFR-CRISPRa cells). We anticipated that the $BRAF^{V600E}$ channel would be a primary driver of pERK levels in the absence of EGF (Fig 9A) and the RAS channel in the presence of EGF (Fig 9B). To instantiate MARM2.0 for HT29 cells, we rescaled baseline protein and mRNA expression levels according to relative

© 2023 The Authors

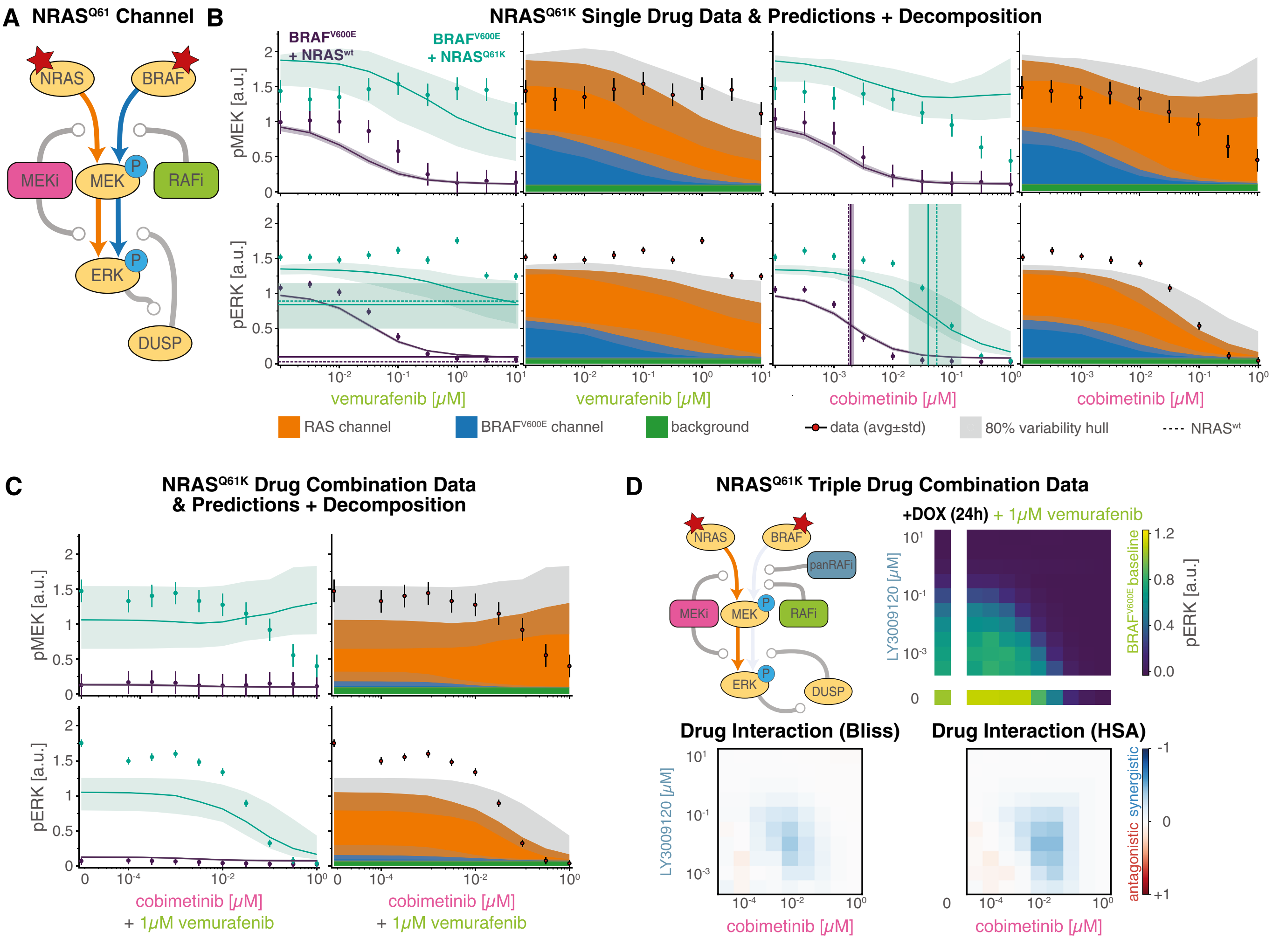

Figure 8. Prediction of drug response in the presence of an NRAS^{Q61K} mutations.

Experiments were performed in A375 cell line with a Dox-inducible NRAS^{Q61K} mutation in 5% FBS medium after 24 h of Dox induction (where applicable) plus 24 h of drug adaptation. EGF stimulation was added to 100 ng/ml. Data are shown as point-ranges with average over technical replicates (n = 2) as points and estimated standard deviation (over all datapoints) as lines.

A Sketch of simplified model topology induced by NRAS^{Q61K} mutation.

B, C Comparison of pMEK (top) and pERK (bottom) dose response predictions and experimental validation. Left panels show Dox induced (cyan) and uninduced (purple) conditions. Right panels show decomposed model simulations for Dox induced conditions as colored areas (blue: BRAF^{V600E} channel, orange: RAS channel) and for uninduced conditions as white dashed lines. Thick lines or stacked areas show median simulations and shading indicates 80% percentiles over 50 best parameter sets. Thin vertical lines indicate EC_{50} values, horizontal lines indicate E_{max} values (data: dashed, model: solid).

D Combination dose response to the triple combination of 1 μM RAFi (vemurafenib) plus varying doses of panRAFi (LY3009120) and MEKi (cobimetinib) in Dox induced cells. Drug interaction analysis via Bliss (bottom left) and HSA (bottom right).

abundances in proteomic and transcriptomic data from the Cancer Cell Line Encyclopedia (Barretina *et al*, 2012; Nusinow *et al*, 2020). For simplicity, we did not account for the heterozygosity of the BRAF^{V600E} mutation. We simulated pERK drug response for RAFi plus MEKi combinations for HT29 cells (bottom) and compared this with simulations for A375 CRISPRa-EGFR (top) and Dox inducible NRAS^{Q61K} A375 cells (middle). In all three cell lines, model predictions (left panels) demonstrated pERK inhibition in high-dose combinations, a result confirmed by experimental data (right panels; Fig 9C). Under conditions of EGF-stimulation, model predictions (left panels) and data (right panels) revealed drug-resistant ERK activation (Fig 9D) and an ~10-fold rightward shift in RAFi and MEKi dose–response curves (red arrows). Causal decomposition (Fig 9E) confirmed that these changes in drug potency are a consequence of profound differences between the BRAF^{V600E} (left) and RAS (right) reaction channels.

Discussion

In this article, we develop an ODE-based model (MARM2.0) and a quantitative framework for analyzing "pathway rewiring" during the acquisition of adaptive resistance to RAF and MEK inhibitors in BRAF^{V600E} melanoma. In addition to its translational importance, adaptive drug resistance represent an excellent setting in which to advance the state of the art in mechanistic modeling of intracellular

© 2023 The Authors

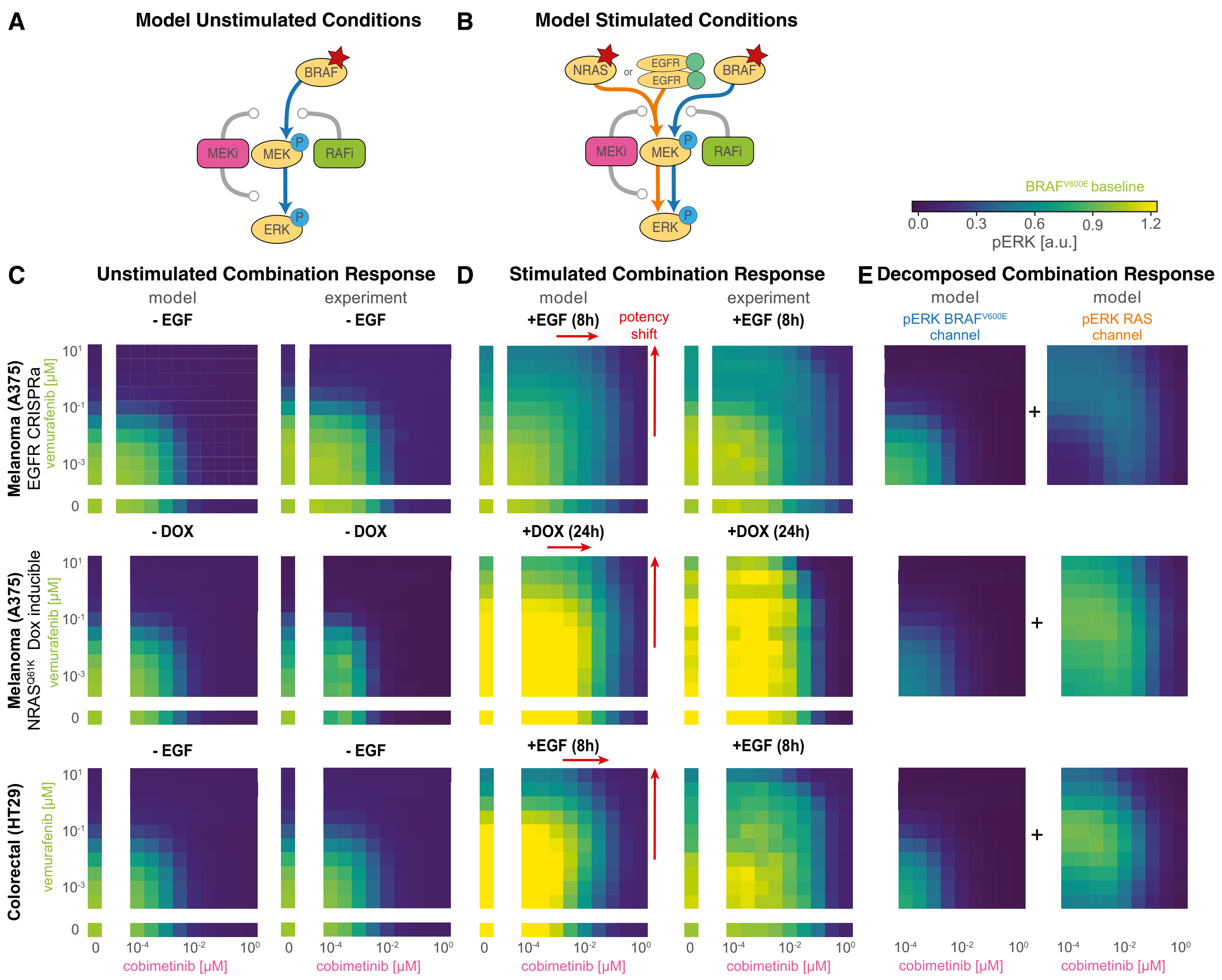

Figure 9. A unified model of drug resistance in BRAF-mutant cancers.

Experiments were performed in 5% (A375) or 10% (HT29) FBS medium after 24 h of Dox induction (where applicable) plus 24 h of drug adaptation. Cells were stimulated with EGF at a final concentration of 100 ng/ml. Data are shown as average over technical replicates (*n* = 2).

- A Schematic illustration of reaction channels under unstimulated conditions shown in (C)
- B Schematic illustration of reaction channels under stimulated conditions shown in (D)
- C, D Predicted (left) and measured (right) pERK in cobimentinib plus vemurafenib for (i) EGFR-CRISPRa amplified A375 melanoma cell line with (D) or without (C) 8 h of EGF stimulation (top row), (ii) NRAS^{Q61K} Dox-inducible A375 melanoma cell line with (D) or without (C) 24 h Dox induction (middle row) and (iii) HT29 colorectal cell line with (D) or without (C) 8 h EGF stimulation (bottom row).
- E Channel decomposition of model predictions shown in (D).

networks: it has been extensively studied using classical molecular biology methods (Solit *et al*, 2006; Hatzivassiliou *et al*, 2010; Poulikakos *et al*, 2010; Lito *et al*, 2012, 2014; Haling *et al*, 2014; Yao *et al*, 2015), which provide extensive structural, biochemical and cell-based data for model formulation. Using MARM2.0, we show that resistance to RAF and MEK inhibitors in BRAF^{V600E} cells can arise from the co-existence of two functionally distinct MAPK reaction channels. Signaling in one channel is initiated by the constitutive activity of oncogenic BRAF^{V600E} and signaling in the other by RAS, which is in turn activated by RTKs. Reaction channels in this context are conceptually similar to "rewired pathways" but the term "reaction channel" (from chemical physics) is a formalization in a mechanistic model of a dynamic state in which transient but distinct assemblies involving enzymes and other regulatory proteins are able to mediate different aspects of signal transduction. The proteins in these channels are largely the same, but they often have different conformations, modes of drug binding, and/or states of post-translational modification.

Depending on conditions, one or the other reaction channel can be dominant in regulating ERK, but the two channels can also operate concurrently, potentially masking each other's activity. For example, in EGF-treated cells, pMEK levels remain roughly constant over a 10^5-fold range of RAFi because MAPK signaling transitions from the BRAF^{V600E} to the RAS channel. The BRAF^{V600E} and RAS

 © 2023 The Authors

channels also influence each other indirectly via control over the synthesis of feedback regulators. An additional feature of these reactions is that they operate on multiple time scales; in the case of the RAS channel this includes: (i) a time scale of seconds to minutes involving post-translational modifications and the direct action of inhibitory drugs (ii) a time scale of tens of minutes involving receptor internalization, degradation and recycling and (iii) a time scale of hours involving changes in the levels of negative feedback regulators such as DUSPS and SPRY. Time-scale separation between signal propagation and transcriptional rewiring is necessary for pulsatile signaling to escape from negative feedback and homeostatic control.

In the treatment of melanoma, RAF and MEK inhibitors are used in combination, which is consistent with the more general use of drug combinations to improve reduce resistance to targeted therapy (Lehár *et al*, 2009). Simulation represents an effective way to investigate mechanisms of drug interactions (Fröhlich *et al*, 2018; Yuan *et al*, 2020) and it has been postulated, on theoretical grounds, that inhibition of enzymes acting sequentially in a pathway is a means to achieve synergistic drug interaction (Fitzgerald *et al*, 2006; Yin *et al*, 2014). However, both data and modeling show that the activities of RAF and MEK inhibitors in $BRAF^{V600E}$ cells are additive over the great majority of the dose–response landscape. In those rare conditions in which drug synergy or antagonism is observed, analysis suggests that transcriptional feedbacks and allosteric interactions —rather than the presence of a serial network motif *per se*—are responsible for drug interaction.

Methodological innovation

Methodological innovation in the current article focuses on combining rule-based modeling based on PySB and BNG with thermodynamic formalisms that exploit the fact that protein–protein and protein-small molecule binding and unbinding events do not consume energy. This builds on the work of Kholodenko on structure-based ODE models (Kholodenko, 2015) while creating a general-purpose framework for programmatically generating model families that make model calibration more efficient. In particular, submodels were generated in PySB to optimally exploit the perturbational structure of the training data (the inclusion or not of drugs and growth factors in each experiment) and combined this with multi-model parameter estimation in the pyPESTO toolbox to substantially accelerate model training, an important consideration with large ODE models and complex training data. Furthermore, PySB/BNG enabled us to implement a labelling scheme for causal network decomposition that traces how species such as activated pERK are generated by converging upstream sequences of activating events (which define downstream reaction channels). Analogous generation and analysis of causal traces ("stories") has been described in agent-based modeling (Boutillier *et al*, 2018) and their adaptation to the MARM2.0 ODE model was essential for formalizing the concept of network rewiring. As this approach provides a low dimensional, interpretable representation of "signal flow" in a model, we expect it to be generally useful for the analysis and interpretation of other large and complex ODE models. These methods are applicable to any PySB model (Lopez *et al*, 2013), and are complemented by even more general methods such as gain analysis.

Using energies (ΔG and ΔΔG values) to describe molecular interactions is a more natural and extendable framework for parameterizing biochemical models than kinetic rates and is likely to form the foundation of a structure-aware approach to dynamic modeling. Energetic landscapes can be estimated from structural studies, from mass-spectrometry measurements (Mason & Covert, 2018; de Souza & Picotti, 2020), and increasingly from folding and docking algorithms that combine biophysical understanding of protein structure with deep learning (AlQuraishi & Sorger, 2021; Jumper *et al*, 2021). Approximate energy values can also mitigate the parametric uncertainty that is pervasive in dynamical models: We anticipate that future use of measured or predicted energy values will make it possible to place fairly tight priors on parameter values during model calibration, generating more predictive and interpretable models. Moreover, the use of energy-based methods promises to bridge between fine-grained atomistic and structural data on single proteins and the more coarse-grained description of biomolecular interactions that are used for dynamical modeling of cellular networks. Ultimately, structure-informed dynamic models could be used to discover emergent cell-level properties such as drug resistance, closing the structure–function gap.

Limitations

At the current state of the art, ODE-based dynamical models are unable to include all known or suspected molecular mechanisms for even well-studied biological networks. This is most obvious at the level of protein and mRNA species: MARM2.0 includes only 11 proteins, three mRNA species and three small molecule drugs but it nonetheless involves a network of over 10^5 distinct biochemical reactions. This represents a substantial increase in complexity relative to previous models of MAPK signaling, but even so, the details of $BRAF^{V600E}$-BRAF-CRAF interaction in the presence and absence of drugs omit many structurally distinct but functionally and kinetically similar modes of kinase activation and inhibition (as described in the literature). Nonetheless, MARM2.0 is applicable not only to $BRAF^{V600E}$ melanoma cells (with and without EGFR overexpression) under a wide variety of conditions but also, with small modifications, to $BRAF^{V600E}$ $NRAS^{Q61K}$ melanoma and $BRAF^{V600E}$ colorectal cancer cells. The good agreement between MARM2.0 simulations and data across these four different settings suggests that the model provides a sufficient explanation of much of the underlying MAPK biochemistry. Conversely, we conclude that a substantial fraction of the detailed molecular mechanisms obtained using vitro studies with purified enzymes or inferred from crystal structures of drug-bound enzymes do not give rise to properties that can be measured at the level of cells, even with the aid of computational models. For example, the variety of phosphorylation-dependent negative feedback mechanisms operating on MAPK signaling are not readily distinguishable from each other under most conditions, making it difficult to discern their different roles. Thus, it is not only technical limitations in model simulation and training that introduces an unsatisfactory degree of personal choice into the formulation of dynamical network models but also experimental non-observability.

Our data contain several clues about conditions in which the MARM2.0 model breaks down and further extensions are necessary. For example, when cells are treated with multiple ligands or drugs in combination ($BRAF^{V600E}$ $NRAS^{Q61K}$ cells exposed to multiple kinase inhibitors, for example) simulations do not closely match empirical data. This is likely to do the absence of molecular

© 2023 The Authors

mechanisms such as KSR scaffolding proteins (McKay *et al*, 2009, 2011a, 2011b; Brennan *et al*, 2011; Dhawan *et al*, 2016; Khan *et al*, 2020), cross-inhibitor allosteric interactions (Rukhlenko *et al*, 2018) as well as higher order interactions between RAF, MEK and MEKis (Pino *et al*, 2021). Moreover, MARM2.0 is not currently able to distinguish between heterozygosity vs. homozygosity of $BRAF^{V600E}$ alleles or resistance mechanisms involving BRAF splice variants (Poulikakos *et al*, 2011), nuclear-cytoplasmic shuttling of MEK and ERK (Fujioka *et al*, 2006), or new drugs targeting RAS. The rule-based formulation of MARM2.0 makes it straightforward to extend the model to specific new use cases. A key challenge for models that include such additional mechanisms is to develop appropriate experimental assays and conditions for measuring their effects. A universal model of MAPK signaling that includes experimental data still remains out of reach since model training would currently be computationally intractable. Systematic methods for coarse graining that account for mechanistic details without explicitly encoding them in model equations will be crucial next step, as will means for more efficiently using multi-omic data in model training.

Materials and Methods

Cell lines and tissue culture

The following cell lines were used in this study with their source indicated in parenthesis: A375 (ATCC), A375 with CRISPRa EGFR overexpression (constructed from ATCC stock as reported in (Gerosa *et al*, 2020)), HT29 (Merrimack Pharmaceuticals) and A375 with doxycycline-inducible $NRAS^{Q61K}$ (Yao *et al*, 2015; provided by Neal Rosen's lab at Memorial Sloan Kettering Cancer Center). A375 cells were grown in Dulbecco's modified eagle medium with 4.5 g/l D-glucose, 4 mM L-glutamine, and 1 mM sodium pyruvate (DMEM; Corning), supplemented with 5% FBS. HT29 cells were grown in RPMI media with L-glutamine supplemented with 10% FBS (50 ml). All media were supplemented with 1% penicillin and streptomycin. Cells were tested for mycoplasma contamination using the MycoAlert mycoplasma detection kit (Lonza).

Drugs and growth factors

The following chemicals from MedChem Express were dissolved in dimethyl sulfoxide (DMSO) at 10 mM: vemurafenib, LY3009120, AZ-628, cobimetinib. EGF ligand was obtained from Peprotech (cat# 100-15) and prepared in media supplemented with 0.1% bovine serum albumin.

Experimental design for combined genetic, ligand and drug perturbations

A375 cells with CRISPRa EGFR overexpression and HT29 cells were treated with the indicated drugs for 24 h before being stimulated with EGF or mock-media for 8 h. A375 cells with doxycycline-inducible $NRAS^{Q61K}$ were treated with doxycycline (10 μM) or mock-media for 24 h before being treated with the indicated drugs for 24 h.

Immunofluorescence staining, quantitation, and analysis for cell cultures

The following primary and conjugated antibodies with specified vendor, animal sources and catalog numbers were used in immunofluorescence analysis of cells and tissues at the specified dilution ratios: p-ERKT202/Y204 rabbit mAb (Cell Signaling Technology, clone D13.14.4 E, Cat# 4370), 1:800; p-MEKS217/221 rabbit mAb (Cell Signaling Technology, Cat# 9121) 1:200, ANTI-FLAG® mouse mAb (Sigma Aldrich, Cat# F1804), 1:1,000. Immunofluorescence assays for cultured cells were performed using cells seeded in either 96-well plates (Corning Cat#3603) or 384-well plates (CellCarrier Cat#6007558) for 24 h and then treated with compounds or ligands either using a Hewlett-Packard D300 Digital Dispenser or by manual dispensing.

Cells were fixed in 4% PFA for 30 min at room temperature (RT) and washed with PBS with 0.1% Tween-20 (Sigma; PBS-T), permeabilized in methanol for 10 min at RT, rewashed with PBS-T, and blocked in Odyssey blocking buffer (OBB LI-COR Cat. No. 927401) for 1 h at RT. Cells were incubated overnight at 4°C with primary antibodies in OBB. Cells were then stained with rabbit and/or with mouse secondary antibodies from Molecular Probes (Invitrogen) labeled with Alexa Fluor 647 (Cat# A31573) or Alexa Fluor 488 (Cat# A21202) both at 1:2,000 dilution. Cells were washed with PBS-T and then PBS and were next incubated in 250 ng/ml Hoechst 33342 and 1:2,000 HCS CellMask™ Blue Stain solution (Thermo Scientific) for 20 min. Cells were washed twice with PBS and imaged with a 10× objective using a PerkinElmer Operetta High Content Imaging System. 9–11 sites were imaged in each well for 96-well plates and 4–6 sites for 384-well plates.

Image segmentation, analysis, and signal intensity quantitation were performed using the Columbus software (PerkinElmer). Cytosol and nuclear areas were identified by using two different thresholds on the CellMask™ Blue Stain (low intensity) and Hoechst channels (~100-fold more intense) were used to define cytosolic and nuclear cell masks, respectively. Cells were identified and enumerated according to successful nuclear segmentation. Unless otherwise specified, immunofluorescence quantifications are average signals of the cytosolic area. In the case of the doxycycline-inducible $NRAS^{Q61K}$ A375 cells, low FLAG intensity was used to remove from analysis cells not expressing FLAG-tagged $NRAS^{Q61K}$: in conditions with doxycycline addition FLAG intensity distributions were markedly bimodal with less than 40% of cells being FLAG negative. Population averages were obtained by averaging values from single-cell segmentation using custom MATLAB 2017a code.

MultiModel fitting

To the best of our knowledge, all state-of-the-art toolboxes only allow for fitting of individual models. To allow for simultaneous training of multiple models, we implemented the *AggregatedObjective* class in pyPESTO (https://github.com/ICB-DCM/pyPESTO), which implements the mapping between global optimization variables as well as respective gradients and local model parameter values and gradients.

To generate the individual model variants, we implemented the function *MARM.model.get_model_instance*, which uses PySB to programmatically remove subsets of initial values of EGF, RAFi and

 © 2023 The Authors

MEKi species. For network generation, we use BNG to construct differential equations only for species with non-zero concentrations. To further reduce computational burden, we implemented the function *MARM.model.cleanup_unused*, which programmatically inspects the generated model and removes unused rules, expressions, parameters and energy patterns.

Model calibration

Model optimization was performed using pyPESTO 0.2.11 (https://doi.org/10.5281/zenodo.5841204) with fides (Fröhlich & Sorger, 2022) version 0.7.5 (https://doi.org/10.5281/zenodo.6038127) as optimizer and AMICI (Fröhlich *et al*, 2021) version 0.11.28 (https://doi.org/10.5281/zenodo.6426308) as simulation engine. 10^3 optimization runs were performed using randomly sampled initial parameter values. Parameter boundaries that were used for initial value sampling and as constraints for optimization are provided in the function *MARM.estimation.get_problem* in the Code EV1. Initial parameter values where objective function values could not be evaluated were resampled until evaluation was possible. Optimization convergence settings were 10^{-12} as step-size tolerance and 10^{-4} as absolute gradient tolerance. Objective function gradients were computed using forward sensitivity analysis. Integration was limited to 10^6 steps and integration tolerances were set to 10^{-11} (absolute) and 10^{-9} (relative). Steady-state tolerances were set to 10^{-9} (absolute) and 10^{-7} (relative).

Causal signal decomposition

To track the causal origin of MEK and ERK phosphorylation, we introduced the concept of reaction channels, which combines ideas from causal pathway analysis (preprint: Babur *et al*, 2018) and causal lineage tracing (Boutillier *et al*, 2018): Causal pathway analysis explains the response to a perturbation by identifying a sequence of regulatory mechanisms consistent with experimental data. This is equivalent to finding a path in the causal analysis graph, constructed from the knowledge graph, that connects the perturbation with the experimentally observed quantity (preprint: Babur *et al*, 2018; Sharp *et al*, 2019). For rule-based models, the causal analysis graph is equivalent to the influence map. Agent based simulations of rule-based models can be represented as random walks on the influence map (Cristescu *et al*, 2019). Accordingly, causal relationships can be extracted by analyzing the traces of individual agents on the knowledge graph (Boutillier *et al*, 2018). As ODE representations of rule-based models describe the average of a population of agents, individual traces are not available and cannot be used to extract causal properties.

To assign phosphorylated MEK and ERK to the $BRAF^{V600E}$ and RAS channels, we added a “channel” site to MEK and ERK molecules, which acts as a tag to track the source of phosphorylation. Upon phosphorylation of MEK, this channel site is set according to the source of phosphorylation “phys” for phosphorylation by RAS bound RAF dimers and “onco” for phosphorylation by mutated BRAF. The rule-based model formulation ensures that the channel information is propagated on all subsequent modeling steps. For the phosphorylation of ERK, we implement two separate rule variants that set the channel site according to the value channel of the phosphorylating MEK molecule. For both pMEK and pERK, the label is set to “NA” during both dephosphorylation and initialization.

Signaling gain

In systems biology, strength of signal transmission is typically quantified as response coefficient or logarithmic gain

$$R = \frac{\frac{\Delta T}{T}}{\frac{\Delta S}{S}}$$

between an input S and an output T at steady-state. However, this definition is not applicable for transient, temporally resolved signals as the response coefficient does not account for the time dimension. As there typically are delays in signal transduction, a pointwise evaluation at individual timepoints does not yield meaningful results.

In signal processing, the gain of linear time invariant systems can be computed as norm of the transfer function G

$$\|G\| = \left\| \frac{L\{T(t)\}}{L\{S(t)\}} \right\|$$

which permits the computation of a gain even for time-resolved inputs $S(s)$ and outputs $T(t)$. However, for nonlinear systems, such as the model we developed, a transfer function generally does not exist. However, we here extend the idea of using functionals such as the Laplace function to map time-resolved input and outputs to scalar values which can then be used to compute the gain. Specifically, we propose the supremum norm

$$\|S\|_\infty = S(t)$$

as well as an L1 norm with exponential time transformation

$$\|S\|_1 = \int_{\log\, t_0}^{\log\, t_f} S(e^t)dt$$

The supremum norm effectively computes the gain evaluated at the peak of the signal, while the L1 norm computes the gain between the area under the curve, where the exponential time transformation aims to avoid problems when signals live on multiple timescales.

The natural scale of gains is the ratio of molecules or concentrations. However, pronounced parameter variability in the estimates for scaling factors, suggested that absolute molecular concentrations were subject to large uncertainties, which would propagate to these norm estimates. Accordingly, we normalized all gains such that baseline signal transmission had a gain of 1.

To numerically compute supremum and L1 norm, we used 50 log-uniformly spaced time points between 10^{-4} and 10^1 h. The integral was approximated using the *sklearn.auc* function, which uses the trapezoidal rule.

Despite substantial variability in parameter estimates (Fig EV2), we found that the variability in qualitative dependence of gain on RAFi and MEKi concentrations is low. We observed the highest variability in the gain from RAS-GTP to physiological pMEK. This is not surprising, as there is no experimental data on RAS-GTP levels. However, the variability appears to primarily affect the absolute levels of signaling gain and less the shape of the dose response curve. Overall, this indicates that our conclusions were not subject to parameter non-identifiability. Moreover, we found that the

© 2023 The Authors

signaling gain analysis is consistent across different RAFis and MEKis for L1 and L_∞ norms (Fig EV4A and B), further corroborating the validity of the approach.

Predictions for NRAS mutant cell lines

In lack of quantitative measurements of mutant NRAS protein abundances in cell lines with acquired or mutated NRAS, we inferred respective levels from baseline data. In the model, the NRAS mutation was implemented through a constitutive GTP loading reaction that activates RAF independent of upstream receptor activity. Only the rate of this reaction was estimated when retraining on baseline data from respective cell-lines, while all other parameters were kept fixed.

Computation of EC_{50} and EC_{max} values

EC_{50} and EC_{max} were computed by fitting a three-parameter hill function

$$\mathrm{EC_{min}} - \frac{\mathrm{EC_{min}} - \mathrm{EC_{max}}}{1 + \frac{\mathrm{EC_{50}}}{x}} - y(x)$$

to either experimental data or model simulations, where x are drug concentrations and y are pMEK or pERK levels. EC_{min} (search interval [0, 2.5], initial 0.5) and EC_{max} (search interval [0,1.5], initial $\min(\max(y(x_{\min}), 0), 2.5)$) were estimated on a linear scale while EC_{50} (search interval $[x_{\min}, x_{\max}]$, initial x_{median}) was estimated on a logarithmic scale. *scipy.optimize.least_squares* was used for curve fitting.

Computation of Bliss and highest single agent synergy scores

To compute drug interaction scores I according to a Bliss Independence null model, we used

$$I_{\mathrm{Bliss}} = E_{AB} - (E_A + E_B - E_A \times E_B),$$

where E_A and E_B are the effects of individual drugs and E_{AB} is the combined drug effects. For the overall pERK signal, the drug effect E_X was computed by normalizating drug perturbed pERK levels $pERK_X$ with baseline pERK levels $pERK_0$:

$$E_X = \frac{pERK_0 - pERK_X}{pERK_0}$$

When computing Bliss synergy scores for RAS channel pERK, normalization was omitted to avoid division by 0. Highest Single Agent interaction scores were computed according to the formula

$$I_{HSA} = E_{AB} - \max(E_A, E_B).$$

Data availability

The datasets and computer code produced in this study are available in the following databases:

- Modeling and visualization computer scripts: GitHub (github.com/labsyspharm/marm2-supplement)
- Modeling and visualization computer scripts: Zenodo (https://zenodo.org/record/6979792#.YvOTSy8RqAk)
- Model files: BioModels (https://www.ebi.ac.uk/biomodels/MODEL2207130001)
- Primary and Processed Immunofluorescence data: Synapse (synapse.org/#!Synapse:syn32830920)

Expanded View for this article is available online.

Acknowledgements

We thank Eduardo Sontag and Pencho Yordanov for helpful discussions. We thank Chris Chidley, Gabriela Sanchez and Sreeram Vallabhaneni for help with experiments. We thank Zhan Yao and Neal Rosen at MSKCC for providing the $NRAS^{Q61K}$ inducible A375 cell line. We thank the Nikon Imaging Center at HMS for assistance with microscopy and the O_2 High Performance Compute Cluster for computing support. The work was funded by National Cancer Institute grant U54-CA225088 (PKS), a Novartis Foundation fellowship (LG), Human Frontier Science Program grant LT000259/2019-L1 (FF).

Author contributions

Fabian Fröhlich: Conceptualization; software; formal analysis; funding acquisition; validation; visualization; methodology; writing – original draft; writing – review and editing. **Luca Gerosa:** Conceptualization; data curation; funding acquisition; validation; investigation; methodology; writing – original draft; writing – review and editing. **Jeremy Muhlich:** Software; writing – review and editing. **Peter K Sorger:** Resources; supervision; funding acquisition; writing – original draft; project administration; writing – review and editing.

Disclosure and competing interests statement

PKS is a member of the SAB or Board of Directors of Glencoe Software, Applied Biomath, and RareCyte Inc. and has equity in these companies; PKS is also a member of the SAB of NanoString and a consultant for Montai Health and Merck. LG is currently an employee of Genentech. PKS and LG declare that none of these relationships are directly or indirectly related to the content of this article. PKS is an editorial advisory board member. This has no bearing on the editorial consideration of this article for publication.

References

Aldridge BB, Burke JM, Lauffenburger DA, Sorger PK (2006) Physicochemical modelling of cell signalling pathways. *Nat Cell Biol* 8: 1195–1203

AlQuraishi M, Sorger PK (2021) Differentiable biology: using deep learning for biophysics-based and data-driven modeling of molecular mechanisms. *Nat Methods* 18: 1169–1180

Alwan HAJ, van Zoelen EJJ, van Leeuwen JEM (2003) Ligand-induced lysosomal epidermal growth factor receptor (EGFR) degradation is preceded by proteasome-dependent EGFR De-ubiquitination. *J Biol Chem* 278: 35781–35790

Arrhenius S (1889) Über die Reaktionsgeschwindigkeit bei der Inversion von Rohrzucker durch Säuren. *Z Für Phys Chem* 4U: 226–248

Babur Ö, Luna A, Korkut A, Durupinar F, Siper MC, Dogrusoz U, Aslan JE, Sander C, Demir E (2018) Causal interactions from proteomic profiles: molecular data meets pathway knowledge. *bioRxiv* https://doi.org/10.1101/258855 [PREPRINT]

 © 2023 The Authors

Baljuls A, Kholodenko BN, Kolch W (2013) It takes two to tango – signalling by dimeric Raf kinases. *Mol Biosyst* 9: 551–558

Barretina J, Caponigro G, Stransky N, Venkatesan K, Margolin AA, Kim S, Wilson CJ, Lehár J, Kryukov GV, Sonkin D *et al* (2012) The cancer cell line encyclopedia enables predictive modelling of anticancer drug sensitivity. *Nature* 483: 603–607

Batzer AG, Rotin D, Ureña JM, Skolnik EY, Schlessinger J (1994) Hierarchy of binding sites for Grb2 and Shc on the epidermal growth factor receptor. *Mol Cell Biol* 14: 5192–5201

Becker V, Schilling M, Bachmann J, Baumann U, Raue A, Maiwald T, Timmer J, Klingmüller U (2010) Covering a broad dynamic range: information processing at the erythropoietin receptor. *Science* 328: 1404–1408

Blinov ML, Faeder JR, Goldstein B, Hlavacek WS (2004) BioNetGen: software for rule-based modeling of signal transduction based on the interactions of molecular domains. *Bioinformatics* 20: 3289–3291

Blinov ML, Faeder JR, Goldstein B, Hlavacek WS (2006) A network model of early events in epidermal growth factor receptor signaling that accounts for combinatorial complexity. *Biosystems* 83: 136–151

Bliss CI (1939) The toxicity of poisons applied jointly. *Ann Appl Biol* 26: 585–615

Boutillier P, Maasha M, Li X, Medina-Abarca HF, Krivine J, Feret J, Cristescu I, Forbes AG, Fontana W (2018) The kappa platform for rule-based modeling. *Bioinformatics* 34: i583–i592

Brennan DF, Dar AC, Hertz NT, Chao WCH, Burlingame AL, Shokat KM, Barford D (2011) A Raf-induced allosteric transition of KSR stimulates phosphorylation of MEK. *Nature* 472: 366–369

Burd CE, Liu W, Huynh MV, Waqas MA, Gillahan JE, Clark KS, Fu K, Martin BL, Jeck WR, Souroullas GP *et al* (2014) Mutation-specific RAS Oncogenicity explains NRAS codon 61 selection in melanoma. *Cancer Discov* 4: 1418–1429

Burotto M, Chiou VL, Lee J-M, Kohn EC (2014) The MAPK pathway across different malignancies: a new perspective. *Cancer* 120: 3446–3456

Chen WW, Schoeberl B, Jasper PJ, Niepel M, Nielsen UB, Lauffenburger DA, Sorger PK (2009) Input–output behavior of ErbB signaling pathways as revealed by a mass action model trained against dynamic data. *Mol Syst Biol* 5: 239

Chis O-T, Banga JR, Balsa-Canto E (2011) Structural identifiability of systems biology models: a critical comparison of methods. *PLoS ONE* 6: e27755

Chou T-C, Tan Q-H, Sirotnak FM (1993) Quantitation of the synergistic interaction of edatrexate and cisplatin in vitro. *Cancer Chemother Pharmacol* 31: 259–264

Clarke CN, Kopetz ES (2015) BRAF mutant colorectal cancer as a distinct subset of colorectal cancer: clinical characteristics, clinical behavior, and response to targeted therapies. *J Gastrointest Oncol* 6: 660–667

Corbalan-Garcia S, Yang SS, Degenhardt KR, Bar-Sagi D (1996) Identification of the mitogen-activated protein kinase phosphorylation sites on human Sos1 that regulate interaction with Grb2. *Mol Cell Biol* 16: 5674–5682

Cristescu I, Fontana W, Krivine J (2019) Interactions between causal structures in graph rewriting systems. *Electron Proc Theor Comput Sci* 286: 65–78

Davies H, Bignell GR, Cox C, Stephens P, Edkins S, Clegg S, Teague J, Woffendin H, Garnett MJ, Bottomley W *et al* (2002) Mutations of the BRAF gene in human cancer. *Nature* 417: 949–954

de Souza N, Picotti P (2020) Mass spectrometry analysis of the structural proteome. *Curr Opin Struct Biol* 60: 57–65

Dessauges C, Mikelson J, Dobrzyński M, Jacques M-A, Frismantiene A, Gagliardi PA, Khammash M, Pertz O (2021) Optogenetic actuator/biosensor circuits for large-scale interrogation of ERK dynamics identify sources of MAPK signaling robustness. *bioRxiv* https://doi.org/10.1101/2021.07.27.453955 [PREPRINT]

Dhawan NS, Scopton AP, Dar AC (2016) Small molecule stabilization of the KSR inactive state antagonizes oncogenic Ras signalling. *Nature* 537: 112–116

Ding K-F, Finlay D, Yin H, Hendricks WPD, Sereduk C, Kiefer J, Sekulic A, LoRusso PM, Vuori K, Trent JM *et al* (2018) Network rewiring in cancer: applications to melanoma cell Lines and the cancer genome atlas patients. *Front Genet* 9: 228

Dougherty MK, Müller J, Ritt DA, Zhou M, Zhou XZ, Copeland TD, Conrads TP, Veenstra TD, Lu KP, Morrison DK (2005) Regulation of Raf-1 by direct feedback phosphorylation. *Mol Cell* 17: 215–224

English JM, Cobb MH (2002) Pharmacological inhibitors of MAPK pathways. *Trends Pharmacol Sci* 23: 40–45

Evans MG, Polanyi M (1935) Some applications of the transition state method to the calculation of reaction velocities, especially in solution. *Trans Faraday Soc* 31: 875–894

Eydgahi H, Chen WW, Muhlich JL, Vitkup D, Tsitsiklis JN, Sorger PK (2013) Properties of cell death models calibrated and compared using Bayesian approaches. *Mol Syst Biol* 9: 644

Eyring H (1935) The activated complex in chemical reactions. *J Chem Phys* 3: 107–115

Faeder JR, Blinov ML, Goldstein B, Hlavacek WS (2005) Combinatorial complexity and dynamical restriction of network flows in signal transduction. *Syst Biol* 2: 5–15

Fallahi-Sichani M, Becker V, Izar B, Baker GJ, Lin J, Boswell SA, Shah P, Rotem A, Garraway LA, Sorger PK (2017) Adaptive resistance of melanoma cells to RAF inhibition via reversible induction of a slowly dividing de-differentiated state. *Mol Syst Biol* 13: 905

Fitzgerald JB, Schoeberl B, Nielsen UB, Sorger PK (2006) Systems biology and combination therapy in the quest for clinical efficacy. *Nat Chem Biol* 2: 458–466

Flaherty KT, Infante JR, Daud A, Gonzalez R, Kefford RF, Sosman J, Hamid O, Schuchter L, Cebon J, Ibrahim N *et al* (2012) Combined BRAF and MEK inhibition in melanoma with BRAF V600 mutations. *N Engl J Med* 367: 1694–1703

Fröhlich F, Sorger PK (2022) Fides: reliable trust-region optimization for parameter estimation of ordinary differential equation models. *PLoS Comput Biol* 18: e1010322

Fröhlich F, Theis FJ, Hasenauer J (2014) Uncertainty analysis for non-identifiable dynamical systems: profile likelihoods, bootstrapping and more. In *Proceedings of the 12th international conference on computational methods in systems biology (CMSB 2014), Manchester, UK*, Mendes P, Dada JO, Smallbone KO (eds), pp 61–72. Cham: Springer International Publishing Switzerland

Fröhlich F, Thomas P, Kazeroonian A, Theis FJ, Grima R, Hasenauer J (2016) Inference for stochastic chemical kinetics using moment equations and system size expansion. *PLoS Comput Biol* 12: e1005030

Fröhlich F, Kaltenbacher B, Theis FJ, Hasenauer J (2017) Scalable parameter estimation for genome-scale biochemical reaction networks. *PLoS Comput Biol* 13: 1–18

Fröhlich F, Kessler T, Weindl D, Shadrin A, Schmiester L, Hache H, Muradyan A, Schütte M, Lim J-H, Heinig M *et al* (2018) Efficient parameter estimation enables the prediction of drug response using a mechanistic pan-cancer pathway model. *Cell Syst* 7: 567–579

Fröhlich F, Loos C, Hasenauer J (2019) Scalable inference of ordinary differential equation models of biochemical processes. In *Gene regulatory networks: methods and protocols*, Sanguinetti G, Huynh-Thu VA (eds), pp 385–422. New York, NY: Springer

Fröhlich F, Weindl D, Schälte Y, Pathirana D, Paszkowski Ł, Lines GT, Stapor P, Hasenauer J (2021) AMICI: high-performance sensitivity analysis for large ordinary differential equation models. *Bioinformatics* 37: 3676–3677

Fujioka A, Terai K, Itoh RE, Aoki K, Nakamura T, Kuroda S, Nishida E, Matsuda M (2006) Dynamics of the Ras/ERK MAPK Cascade as monitored by fluorescent probes. *J Biol Chem* 281: 8917–8926

Gao Y, Chang MT, McKay D, Na N, Zhou B, Yaeger R, Torres NM, Muniz K, Drosten M, Barbacid M *et al* (2018) Allele-specific mechanisms of activation of MEK1 mutants determine their properties. *Cancer Discov* 8: 648–661

Gawthrop PJ, Crampin EJ (2017) Energy-based analysis of biomolecular pathways. *Proc Math Phys Eng Sci* 473: 20160825

Gerosa L, Chidley C, Fröhlich F, Sanchez G, Lim SK, Muhlich J, Chen J-Y, Vallabhaneni S, Baker GJ, Schapiro D *et al* (2020) Receptor-driven ERK pulses reconfigure MAPK signaling and enable persistence of drug-adapted BRAF-mutant melanoma cells. *Cell Syst* 11: 478–494

Goldbeter A, Koshland DE (1981) An amplified sensitivity arising from covalent modification in biological systems. *Proc Natl Acad Sci USA* 78: 6840–6844

Gollub MG, Kaltenbach H-M, Stelling J (2021) Probabilistic thermodynamic analysis of metabolic networks. *Bioinformatics* 37: 2938–2945

Gutenkunst RN, Waterfall JJ, Casey FP, Brown KS, Myers CR, Sethna JP (2007) Universally sloppy parameter sensitivities in systems biology models. *PLoS Comput Biol* 3: 1871–1878

Haling JR, Sudhamsu J, Yen I, Sideris S, Sandoval W, Phung W, Bravo BJ, Giannetti AM, Peck A, Masselot A *et al* (2014) Structure of the BRAF-MEK complex reveals a kinase activity independent role for BRAF in MAPK signaling. *Cancer Cell* 26: 402–413

Hall-Jackson CA, Eyers PA, Cohen P, Goedert M, Tom Boyle F, Hewitt N, Plant H, Hedge P (1999) Paradoxical activation of Raf by a novel Raf inhibitor. *Chem Biol* 6: 559–568

Harris LA, Hogg JS, Tapia J-J, Sekar JAP, Gupta S, Korsunsky I, Arora A, Barua D, Sheehan RP, Faeder JR (2016) BioNetGen 2.2: advances in rule-based modeling. *Bioinformatics* 32: 3366–3368

Hatzivassiliou G, Song K, Yen I, Brandhuber BJ, Anderson DJ, Alvarado R, Ludlam MJC, Stokoe D, Gloor SL, Vigers G *et al* (2010) RAF inhibitors prime wild-type RAF to activate the MAPK pathway and enhance growth. *Nature* 464: 431–435

Hatzivassiliou G, Haling JR, Chen H, Song K, Price S, Heald R, Hewitt JFM, Zak M, Peck A, Orr C *et al* (2013) Mechanism of MEK inhibition determines efficacy in mutant KRAS- versus BRAF-driven cancers. *Nature* 501: 232–236

Henry JR, Kaufman MD, Peng S-B, Ahn YM, Caldwell TM, Vogeti L, Telikepalli H, Lu W-P, Hood MM, Rutkoski TJ *et al* (2015) Discovery of 1-(3,3-Dimethylbutyl)-3-(2-fluoro-4-methyl-5-(7-methyl-2-(methylamino)pyrido[2,3-d]pyrimidin-6-yl)phenyl)urea (LY3009120) as a pan-RAF inhibitor with minimal paradoxical activation and activity against BRAF or RAS mutant tumor cells. *J Med Chem* 58: 4165–4179

Hlavacek WS, Faeder JR, Blinov ML, Posner RG, Hucka M, Fontana W (2006) Rules for modeling signal-transduction systems. *Sci STKE* 2006: re6

Hogg JS (2013) Advances in rule-based modeling: compartments, energy, and hybrid simulation, with application to sepsis and cell signaling. *Undefined*

Honorato-Zimmer R, Harmer R, Danos V (2015) Thermodynamic graph-rewriting. *Log Methods Comput Sci* 11: https://doi.org/10.2168/LMCS-11(2:13)2015

Hunter T (2000) Signaling—2000 and Beyond. *Cell* 100: 113–127

Hyttinen A, Eberhardt F, Hoyer PO (2012) Learning linear cyclic causal models with latent variables. *J Mach Learn Res* 13: 3387–3439

Jumper J, Evans R, Pritzel A, Green T, Figurnov M, Ronneberger O, Tunyasuvunakool K, Bates R, Žídek A, Potapenko A *et al* (2021) Highly accurate protein structure prediction with AlphaFold. *Nature* 596: 1–11

Kamioka Y, Yasuda S, Fujita Y, Aoki K, Matsuda M (2010) Multiple decisive phosphorylation sites for the negative feedback regulation of SOS1 via ERK. *J Biol Chem* 285: 33540–33548

Karoulia Z, Gavathiotis E, Poulikakos PI (2017) New perspectives for targeting RAF kinase in human cancer. *Nat Rev Cancer* 17: 676–691

Kebebew E, Weng J, Bauer J, Ranvier G, Clark OH, Duh Q-Y, Shibru D, Bastian B, Griffin A (2007) The prevalence and prognostic value of BRAF mutation in thyroid cancer. *Ann Surg* 246: 466–471

Khan ZM, Real AM, Marsiglia WM, Chow A, Duffy ME, Yerabolu JR, Scopton AP, Dar AC (2020) Structural basis for the action of the drug trametinib at KSR-bound MEK. *Nature* 588: 509–514

Kholodenko BN (2015) Drug resistance resulting from kinase dimerization is rationalized by thermodynamic factors describing allosteric inhibitor effects. *Cell Rep* 12: 1939–1949

Kholodenko BN, Demin OV, Moehren G, Hoek JB (1999) Quantification of Short term signaling by the epidermal growth factor receptor. *J Biol Chem* 274: 30169–30181

Kiyatkin A, van Rosenburgh IKA, Klein DE, Lemmon MA (2020) Kinetics of receptor tyrosine kinase activation define ERK signaling dynamics. *Sci Signal* 13: eaaz5267

Kleiman LB, Maiwald T, Conzelmann H, Lauffenburger DA, Sorger PK (2011) Rapid phospho-turnover by receptor tyrosine kinases impacts downstream signaling and drug binding. *Mol Cell* 43: 723–737

Klosin A, Oltsch F, Harmon T, Honigmann A, Jülicher F, Hyman AA, Zechner C (2020) Phase separation provides a mechanism to reduce noise in cells. *Science* 367: 464–468

Kreutz C, Raue A, Timmer J (2012) Likelihood based observability analysis and confidence intervals for predictions of dynamic models. *BMC Syst Biol* 6: 120

Lao D-H, Chandramouli S, Yusoff P, Fong CW, Saw TY, Tai LP, Yu CY, Leong HF, Guy GR (2006) A Src homology 3-binding sequence on the C terminus of Sprouty2 is necessary for inhibition of the Ras/ERK pathway downstream of fibroblast growth factor receptor stimulation. *J Biol Chem* 281: 29993–30000

Lavoie H, Therrien M (2015) Regulation of RAF protein kinases in ERK signalling. *Nat Rev Mol Cell Biol* 16: 281–298

Lavoie H, Gagnon J, Therrien M (2020) ERK signalling: a master regulator of cell behaviour, life and fate. *Nat Rev Mol Cell Biol* 21: 607–632

Lee MJ, Ye AS, Gardino AK, Heijink AM, Sorger PK, MacBeath G, Yaffe MB (2012) Sequential application of anticancer drugs enhances cell death by rewiring apoptotic signaling networks. *Cell* 149: 780–794

Lehár J, Zimmermann GR, Krueger AS, Molnar RA, Ledell JT, Heilbut AM, Short GF, Giusti LC, Nolan GP, Magid OA *et al* (2007) Chemical combination effects predict connectivity in biological systems. *Mol Syst Biol* 3: 80

Lehár J, Krueger AS, Avery W, Heilbut AM, Johansen LM, Price ER, Rickles RJ, Short Iii GF, Staunton JE, Jin X *et al* (2009) Synergistic drug combinations tend to improve therapeutically relevant selectivity. *Nat Biotechnol* 27: 659–666

Lemmon MA, Schlessinger J (2010) Cell signaling by receptor-tyrosine kinases. *Cell* 141: 1117–1134

Lito P, Pratilas CA, Joseph EW, Tadi M, Halilovic E, Zubrowski M, Huang A, Wong WL, Callahan MK, Merghoub T *et al* (2012) Relief of profound feedback inhibition of Mitogenic signaling by RAF inhibitors attenuates their activity in BRAFV600E melanomas. *Cancer Cell* 22: 668–682

Lito P, Rosen N, Solit DB (2013) Tumor adaptation and resistance to RAF inhibitors. *Nat Med* 19: 1401–1409

Lito P, Saborowski A, Yue J, Solomon M, Joseph E, Gadal S, Saborowski M, Kastenhuber E, Fellmann C, Ohara K *et al* (2014) Disruption of CRAF-

© 2023 The Authors

mediated MEK activation is required for effective MEK inhibition in KRAS mutant tumors. *Cancer Cell* 25: 697–710

Loewe S (1928) Die quantitativen probleme der pharmakologie. *Ergeb Physiol* 27: 47–187

Long GV, Fung C, Menzies AM, Pupo GM, Carlino MS, Hyman J, Shahheydari H, Tembe V, Thompson JF, Saw RP *et al* (2014) Increased MAPK reactivation in early resistance to dabrafenib/trametinib combination therapy of BRAF-mutant metastatic melanoma. *Nat Commun* 5: 1–9

Lopez CF, Muhlich JL, Bachman JA, Sorger PK (2013) Programming biological models in python using PySB. *Mol Syst Biol* 9: 646

Lund KA, Opresko LK, Starbuck C, Walsh BJ, Wiley HS (1990) Quantitative analysis of the endocytic system involved in hormone-induced receptor internalization. *J Biol Chem* 265: 15713–15723

Marin-Bejar O, Rogiers A, Dewaele M, Femel J, Karras P, Pozniak J, Bervoets G, Raemdonck NV, Pedri D, Swings T *et al* (2021) Evolutionary predictability of genetic versus nongenetic resistance to anticancer drugs in melanoma. *Cancer Cell* 39: 1135–1149

Mason JC, Covert MW (2018) An energetic reformulation of kinetic rate laws enables scalable parameter estimation for biochemical networks. *J Theor Biol* 461: 145–156

McKay MM, Ritt DA, Morrison DK (2009) Signaling dynamics of the KSR1 scaffold complex. *Proc Natl Acad Sci USA* 106: 11022–11027

McKay MM, Freeman AK, Morrison DK (2011a) Complexity in KSR function revealed by Raf inhibitor and KSR structure studies. *Small GTPases* 2: 276–281

McKay MM, Ritt DA, Morrison DK (2011b) RAF inhibitor-induced KSR1/B-RAF binding and its effects on ERK Cascade signaling. *Curr Biol* 21: 563–568

Mooij JM, Janzing D, Schölkopf B (2013) From ordinary differential equations to structural causal models: the deterministic case. *ArXiv* https://doi.org/10.48550/arXiv.1304.7920 [PREPRINT]

Noeparast A, Giron P, De Brakeleer S, Eggermont C, De Ridder U, Teugels E, De Grève J (2018) Type II RAF inhibitor causes superior ERK pathway suppression compared to type I RAF inhibitor in cells expressing different BRAF mutant types recurrently found in lung cancer. *Oncotarget* 9: 16110–16123

Nusinow DP, Szpyt J, Ghandi M, Rose CM, McDonald ER, Kalocsay M, Jané-Valbuena J, Gelfand E, Schweppe DK, Jedrychowski M *et al* (2020) Quantitative proteomics of the cancer cell line encyclopedia. *Cell* 180: 387–402

Olivier BG, Rohwer JM, Hofmeyr J-HS (2005) Modelling cellular systems with PySCeS. *Bioinformatics* 21: 560–561

Ollivier JF, Shahrezaei V, Swain PS (2010) Scalable rule-based modelling of allosteric proteins and biochemical networks. *PLoS Comput Biol* 6: e1000975

Oren Y, Tsabar M, Cuoco MS, Amir-Zilberstein L, Cabanos HF, Hütter J-C, Hu B, Thakore PI, Tabaka M, Fulco CP *et al* (2021) Cycling cancer persister cells arise from lineages with distinct programs. *Nature* 596: 576–582

Pearl J, Dechter R (2013) Identifying independencies in causal graphs with feedback. *ArXiv* https://doi.org/10.48550/arXiv.1302.3595 [PREPRINT]

Peng S-B, Henry JR, Kaufman MD, Lu W-P, Smith BD, Vogeti S, Rutkoski TJ, Wise S, Chun L, Zhang Y *et al* (2015) Inhibition of RAF isoforms and active dimers by LY3009120 leads to anti-tumor activities in RAS or BRAF mutant cancers. *Cancer Cell* 28: 384–398

Pino GLG-D, Li K, Park E, Schmoker AM, Ha BH, Eck MJ (2021) Allosteric MEK inhibitors act on BRAF/MEK complexes to block MEK activation. *Proc Natl Acad Sci USA* 118: e2107207118

Poulikakos PI, Zhang C, Bollag G, Shokat KM, Rosen N (2010) RAF inhibitors transactivate RAF dimers and ERK signalling in cells with wild-type BRAF. *Nature* 464: 427–430

Poulikakos PI, Persaud Y, Janakiraman M, Kong X, Ng C, Moriceau G, Shi H, Atefi M, Titz B, Gabay MT *et al* (2011) RAF inhibitor resistance is mediated by dimerization of aberrantly spliced BRAF(V600E). *Nature* 480: 387–390

Pratilas CA, Taylor BS, Ye Q, Viale A, Sander C, Solit DB, Rosen N (2009) (V600E)BRAF is associated with disabled feedback inhibition of RAF-MEK signaling and elevated transcriptional output of the pathway. *Proc Natl Acad Sci USA* 106: 4519–4524

Prior IA, Lewis PD, Mattos C (2012) A comprehensive survey of Ras mutations in cancer. *Cancer Res* 72: 2457–2467

Raue A, Kreutz C, Maiwald T, Klingmüller U, Timmer J (2011) Addressing parameter identifiability by model-based experimentation. *IET Syst Biol* 5: 120–130

Reddy RJ, Gajadhar AS, Swenson EJ, Rothenberg DA, Curran TG, White FM (2016) Early signaling dynamics of the epidermal growth factor receptor. *Proc Natl Acad Sci USA* 113: 3114–3119

Resat H, Ewald JA, Dixon DA, Wiley HS (2003) An integrated model of epidermal growth factor receptor trafficking and signal transduction. *Biophys J* 85: 730–743

Ritt DA, Monson DM, Specht SI, Morrison DK (2010) Impact of feedback phosphorylation and Raf Heterodimerization on Normal and mutant B-Raf signaling. *Mol Cell Biol* 30: 806–819

Roskoski R (2016) Classification of small molecule protein kinase inhibitors based upon the structures of their drug-enzyme complexes. *Pharmacol Res* 103: 26–48

Rukhlenko OS, Khorsand F, Krstic A, Rozanc J, Alexopoulos LG, Rauch N, Erickson KE, Hlavacek WS, Posner RG, Gómez-Coca S *et al* (2018) Dissecting RAF inhibitor resistance by structure-based modeling reveals ways to overcome oncogenic RAS signaling. *Cell Syst* 7: 161–179

Russo M, Crisafulli G, Sogari A, Reilly NM, Arena S, Lamba S, Bartolini A, Amodio V, Magr131 A, Novara L *et al* (2019) Adaptive mutability of colorectal cancers in response to targeted therapies. *Science* 366: 1473–1480

Saha M, Carriere A, Cheerathodi M, Zhang X, Lavoie G, Rush J, Roux PP, Ballif BA (2012) RSK phosphorylates SOS1 creating 14-3-3-docking sites and negatively regulating MAPK activation. *Biochem J* 447: 159–166

Samatar AA, Poulikakos PI (2014) Targeting RAS–ERK signalling in cancer: promises and challenges. *Nat Rev Drug Discov* 13: 928–942

Sanchez-Vega F, Mina M, Armenia J, Chatila WK, Luna A, La KC, Dimitriadoy S, Liu DL, Kantheti HS, Saghafinia S *et al* (2018) Oncogenic signaling pathways in the cancer genome atlas. *Cell* 173: 321–337

Schöberl B, Pace EA, Fitzgerald JB, Harms BD, Xu L, Nie L, Linggi B, Kalra A, Paragas V, Bukhalid R *et al* (2009) Therapeutically targeting ErbB3: a key node in ligand-induced activation of the ErbB receptor-PI3K axis. *Sci Signal* 2: ra31

Schuh L, Saint-Antoine M, Sanford EM, Emert BL, Singh A, Marr C, Raj A, Goyal Y (2020) Gene networks with transcriptional bursting recapitulate rare transient coordinated high expression states in cancer. *Cell Syst* 10: 363–378

Sekar JAP, Hogg JS, Faeder JR (2016) Energy-based modeling in BioNetGen. In *2016 IEEE international conference on bioinformatics and biomedicine (BIBM)*, pp 1460–1467

Shaffer SM, Dunagin MC, Torborg SR, Torre EA, Emert B, Krepler C, Beqiri M, Sproesser K, Brafford PA, Xiao M *et al* (2017) Rare cell variability and drug-induced reprogramming as a mode of cancer drug resistance. *Nature* 546: 431–435

Sharp R, Pyarelal A, Gyori B, Alcock K, Laparra E, Valenzuela-Escárcega MA, Nagesh A, Yadav V, Bachman J, Tang Z *et al* (2019) Eidos, INDRA, & Delphi: from free text to executable causal models. In *Proceedings of the 2019*

conference of the north American chapter of the Association for Computational Linguistics (demonstrations), pp 42–47. Minneapolis, MN: Association for Computational Linguistics

Shi H, Hugo W, Kong X, Hong A, Koya RC, Moriceau G, Chodon T, Guo R, Johnson DB, Dahlman KB *et al* (2014) Acquired resistance and clonal evolution in melanoma during BRAF inhibitor therapy. *Cancer Discov* 4: 80–93

Sneddon MW, Faeder JR, Emonet T (2011) Efficient modeling, simulation and coarse-graining of biological complexity with NFsim. *Nat Methods* 8: 177–183

Solit DB, Garraway LA, Pratilas CA, Sawai A, Getz G, Basso A, Ye Q, Lobo JM, She Y, Osman I *et al* (2006) BRAF mutation predicts sensitivity to MEK inhibition. *Nature* 439: 358–362

Spirtes PL (2013) Directed cyclic graphical representations of feedback models. *ArXiv* https://doi.org/10.48550/arXiv.1302.4982 [PREPRINT]

Städter P, Schälte Y, Schmiester L, Hasenauer J, Stapor PL (2021) Benchmarking of numerical integration methods for ODE models of biological systems. *Sci Rep* 11: 2696

Stapor P, Weindl D, Ballnus B, Hug S, Loos C, Fiedler A, Krause S, Hroß S, Fröhlich F, Hasenauer J (2018) PESTO: parameter EStimation TOolbox. *Bioinformatics* 34: 705–707

Starbuck C, Lauffenburger DA (1992) Mathematical model for the effects of epidermal growth factor receptor trafficking dynamics on fibroblast proliferation responses. *Biotechnol Prog* 8: 132–143

Sullivan RJ, Flaherty K (2012) MAP kinase signaling and inhibition in melanoma. *Oncogene* 32: 2373–2379

Tsai C-J, Nussinov R (2014) A unified view of "how Allostery works". *PLoS Comput Biol* 10: e1003394

Tutuka CSA, Andrews MC, Mariadason JM, Ioannidis P, Hudson C, Cebon J, Behren A (2017) PLX8394, a new generation BRAF inhibitor, selectively inhibits BRAF in colonic adenocarcinoma cells and prevents paradoxical MAPK pathway activation. *Mol Cancer* 16: 112

Ullrich A, Schlessinger J (1990) Signal transduction by receptors with tyrosine kinase activity. *Cell* 61: 203–212

Villaverde AF, Fröhlich F, Weindl D, Hasenauer J, Banga JR (2019) Benchmarking optimization methods for parameter estimation in large kinetic models. *Bioinformatics* 35: 830–838

Wagle N, Emery C, Berger MF, Davis MJ, Sawyer A, Pochanard P, Kehoe SM, Johannessen CM, MacConaill LE, Hahn WC *et al* (2011) Dissecting therapeutic resistance to RAF inhibition in melanoma by tumor genomic profiling. *J Clin Oncol* 29: 3085–3096

Wegscheider R (1911) Über simultane Gleichgewichte und die Beziehungen zwischen Thermodynamik und Reactionskinetik homogener Systeme. *Monatshefte Für Chem Verwandte Teile Anderer Wiss* 32: 849–906

Wei Q, Qian Y, Yu J, Wong CC (2020) Metabolic rewiring in the promotion of cancer metastasis: mechanisms and therapeutic implications. *Oncogene* 39: 6139–6156

Wieland F-G, Hauber AL, Rosenblatt M, Tönsing C, Timmer J (2021) On structural and practical identifiability. *Curr Opin Syst Biol* 25: 60–69

Wiley HS (1988) Anomalous binding of epidermal growth factor to A431 cells is due to the effect of high receptor densities and a saturable endocytic system. *J Cell Biol* 107: 801–810

Wu P-K, Park J-I (2015) MEK1/2 inhibitors: molecular activity and resistance mechanisms. *Semin Oncol* 42: 849–862

Yao Z, Torres NM, Tao A, Gao Y, Luo L, Li Q, de Stanchina E, Abdel-Wahab O, Solit DB, Poulikakos PI *et al* (2015) BRAF mutants evade ERK-dependent feedback by different mechanisms that determine their sensitivity to pharmacologic inhibition. *Cancer Cell* 28: 370–383

Yao Z, Gao Y, Su W, Yaeger R, Tao J, Na N, Zhang Y, Zhang C, Rymar A, Tao A *et al* (2019) RAF inhibitor PLX8394 selectively disrupts BRAF dimers and RAS-independent BRAF-mutant-driven signaling. *Nat Med* 25: 284–291

Yen I, Shanahan F, Merchant M, Orr C, Hunsaker T, Durk M, La H, Zhang X, Martin SE, Lin E *et al* (2018) Pharmacological induction of RAS-GTP confers RAF inhibitor sensitivity in KRAS mutant tumors. *Cancer Cell* 34: 611–625

Yen I, Shanahan F, Lee J, Hong YS, Shin SJ, Moore AR, Sudhamsu J, Chang MT, Bae I, Dela Cruz D *et al* (2021) ARAF mutations confer resistance to the RAF inhibitor belvarafenib in melanoma. *Nature* 594: 418–423

Yin N, Ma W, Pei J, Ouyang Q, Tang C, Lai L (2014) Synergistic and antagonistic drug combinations depend on network topology. *PLoS ONE* 9: e93960

Yuan B, Shen C, Luna A, Korkut A, Marks DS, Ingraham J, Sander C (2020) CellBox: interpretable machine learning for perturbation biology with application to the Design of Cancer Combination Therapy. *Cell Syst* 12: 128–140

Zhang C, Spevak W, Zhang Y, Burton EA, Ma Y, Habets G, Zhang J, Lin J, Ewing T, Matusow B *et al* (2015) RAF inhibitors that evade paradoxical MAPK pathway activation. *Nature* 526: 583–586

License: This is an open access article under the terms of the Creative Commons Attribution License, which permits use, distribution and reproduction in any medium, provided the original work is properly cited.

© 2023 The Authors

Expanded View Figures

Figure EV1. Overview calibrated model simulation and experimental data.

Experiments were performed in A375 cell lines in 5% FBS medium following 24 h of drug adaptation (unless otherwise noted). EGF stimulation was at 100 ng/ml. Data are shown as point-ranges with average over technical replicates ($n = 2$) as point and estimated standard deviation (over all data points) as line. Data from different experiments (biological replicates) are shown separately. Median simulations are shown as thick lines and shading indicates 80% percentiles over 50 best parameter sets.

A Phospoproteomic training data (RAFi dose response). Experiments were performed after 24 h pretreatment with drug/DMSO without EGF stimulation.

B Proteomic training data (RAFi dose response). Experiments were performed after 24 h pretreatment with drug/DMSO without EGF stimulation.

C Transcriptomic training data. RAFi dose response (left three panels) and time course (right three panels). Dose response was performed after 24 h pretreatment with drug/DMSO without EGF stimulation. Time-course measurements were collected after 24 h pretreatment with 1 μM vemurafenib.

D Time resolved RAFi and MEKi drug response immunofluorescence data.

E Time course immunofluorescence data for different pretreatment times (for drug adaption). Pretreatment time indicates the time between drug treatment (1 μM vemurafenib) and EGF stimulation (100 ng/ml).

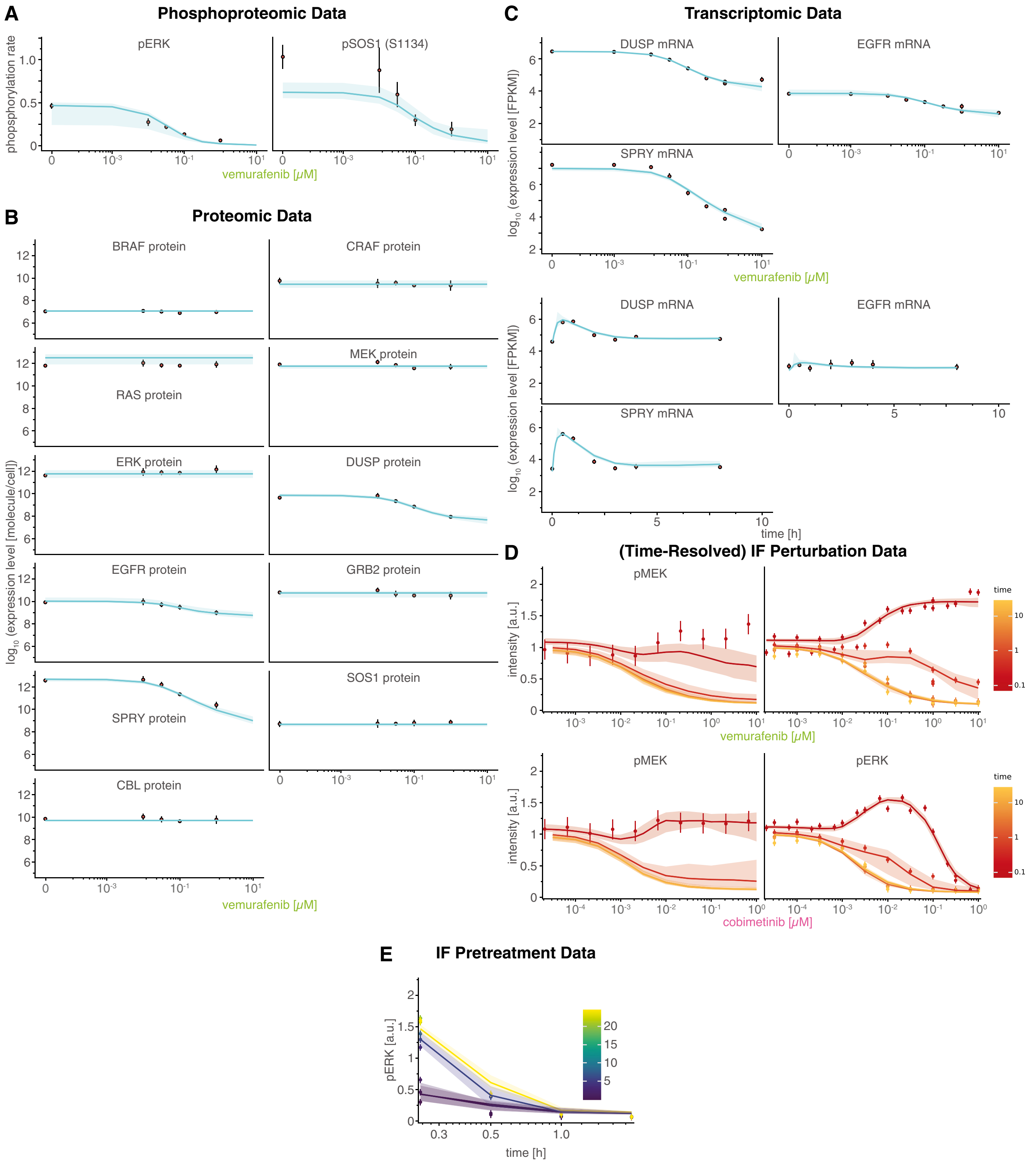

Figure EV1.

 © 2023 The Authors

Figure EV2. Variability in parameter estimates.

A Boxplot of parameter estimates for best 50 parameter sets. Optimization boundary is indicated as dashed lines. Type of parameters are indicated by suffix: _0 (expression level), _dG (thermodynamic encoding of affinity, not on log scale), _ddG (thermodynamic encoding of allosteric interactions, not on log scale), _eq (baseline expression level), _gexpslope (RNA synthesis scaling factor), _kM (pERK concentration at which 50% activation is achieved), _kbase (baseline phosphorylation rate), _kcat (catalytic rate), _kcatr (normalized kcat), _kdeg (degradation rate), _kf (binding rate), _offset (background intensity), _scale (observable scaling). Central band shows media, box extends from lower to upper quartile values and whiskers show full range excluding outliers (points more than 1.5 interquartile ranges away from lower and upper quartiles).

B Correlation plots of parameter estimates. Only statistically significant ($P > 0.05$, Bonferroni-Holm corrected two-tailed t-test) correlations are shown. Coloring shows positive (red)/negative (blue) correlation.

© 2023 The Authors

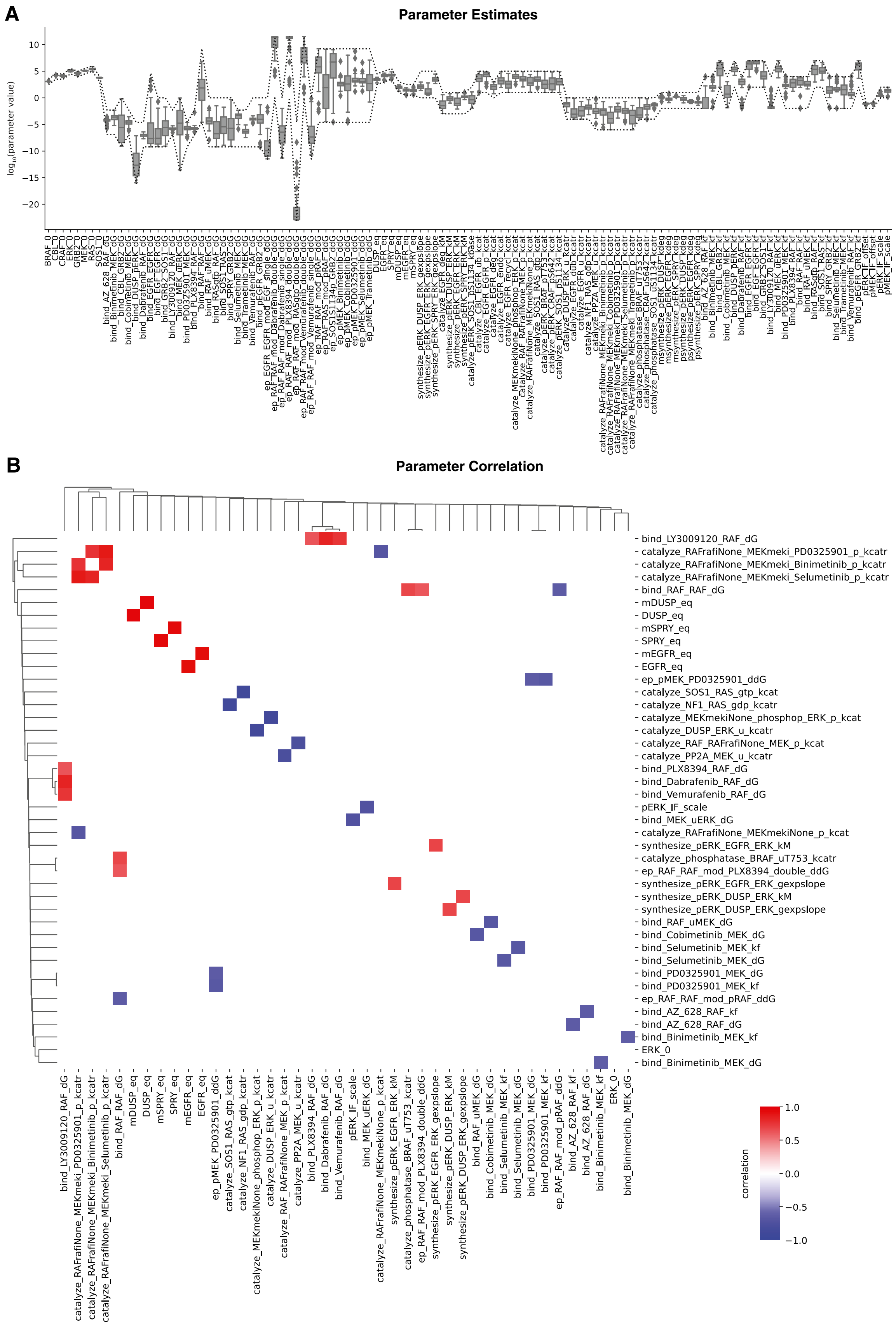

Figure EV2.

© 2023 The Authors

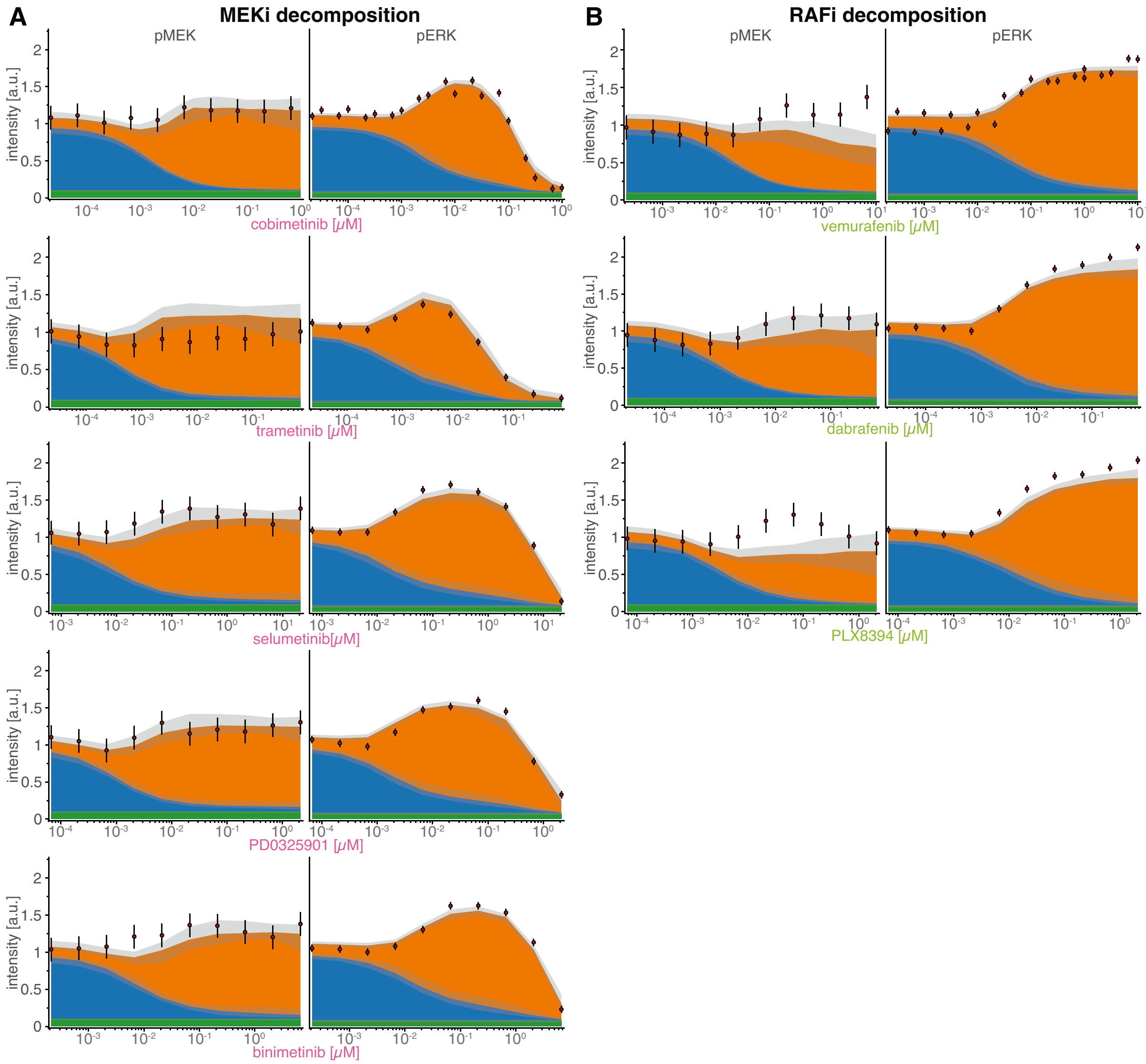

Figure EV3. Causal Decomposition of RAS and BRAF^{V600E} channels (extended).

Experiments were performed in A375 cell lines in 5% FBS medium after 24 h of drug adaptation. EGF stimulation was at 100 ng/ml.

A, B Comparison of experimental data and decomposed model simulations for pERK (left panels) and pMEK (right panels) at 5 min after EGF stimulation for five different MEK inhibitors (A) and three different RAF inhibitors (B). Data are shown as point-ranges with average over technical replicates ($n = 2$) as point and estimated standard deviation (over all data points) as line. Median (over 50 best parameter sets) simulations are shown as stacked areas with color corresponding to channels (blue: BRAF^{V600E}, orange: RAS). Shading indicates 80% percentiles over 50 best parameter sets.

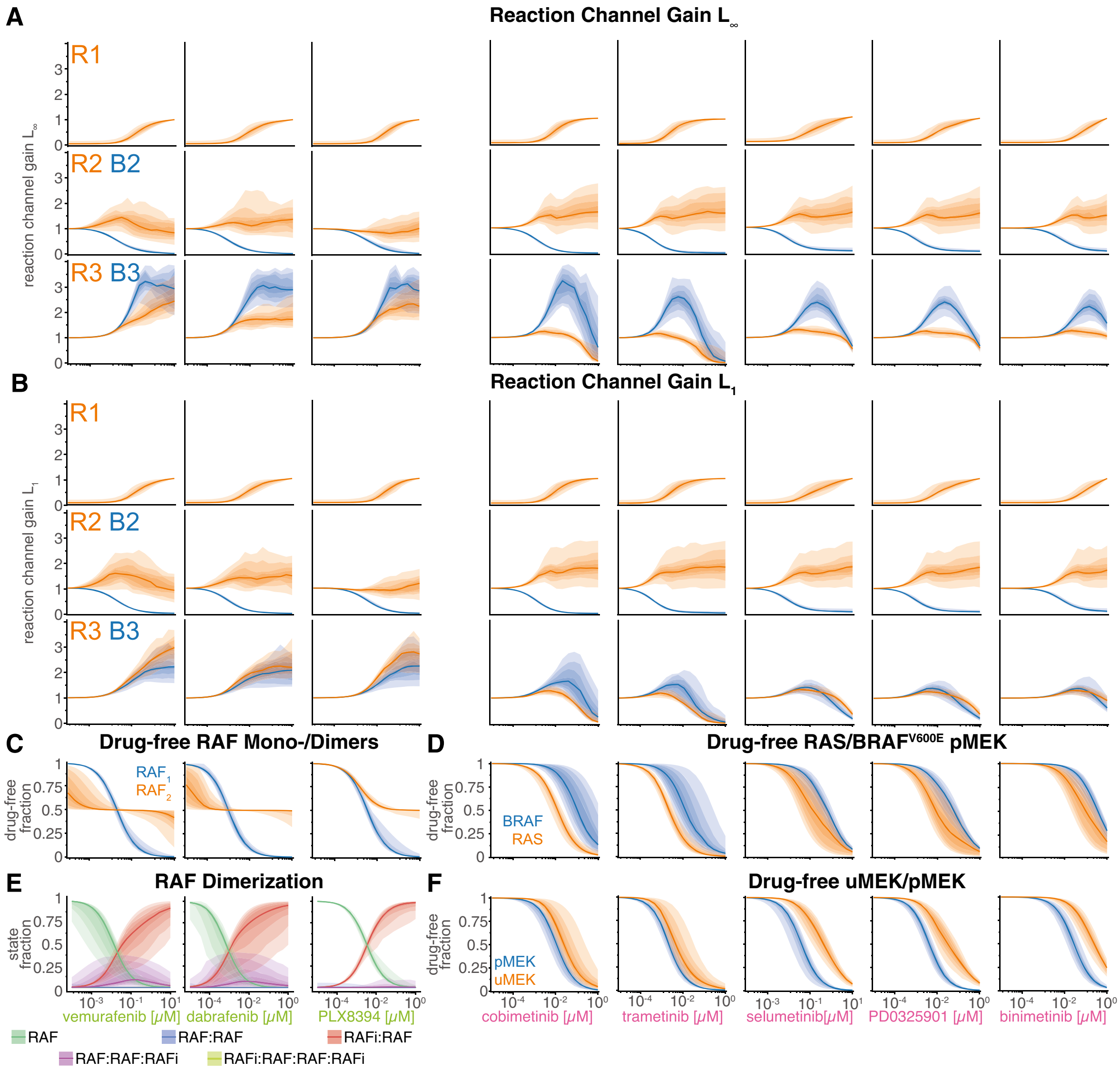

Figure EV4. Quantification of gain in RAS and BRAF^{V600E} channels (extended).

A, B Quantification of signal transmissions in terms of gain (L_1 and L_∞) along the edges of the simplified network in Fig 4A for different concentrations of three different RAF inhibitors (columns 1–3) and five different MEK inhibitors (columns 4–8). Color indicates the reaction channel (blue: BRAF^{V600E}, orange: RAS). Solid lines show median, and shading indicates 20, 40, 60 and 80% percentiles over 50 best parameter sets.

C, D Quantification of drug-free protomer fractions. Columns correspond to different RAFi/MEKi, as in A/B. Colors indicate different complexification (C), reaction channel (D) or post-translational states (F). Solid lines show median, and shading indicates 20, 40, 60 and 80% percentiles over 50 best parameter sets.

E Simulated Assembly of RAF-RAFi complexes in response to different RAFi. Each color corresponds to a different complex. Complex assembly was quantified for RAFi-adapted cells at 5 min after EGF stimulation. Solid line shows median, and shading indicates 20, 40, 60 and 80% percentiles over 50 best parameter sets.

F Quantification of drug-free protomer fractions. Columns correspond to different MEKi, as in A/B. Colors indicate different post-translational states. Solid lines show median, and shading indicates 20, 40, 60 and 80% percentiles over 50 best parameter sets.

 © 2023 The Authors

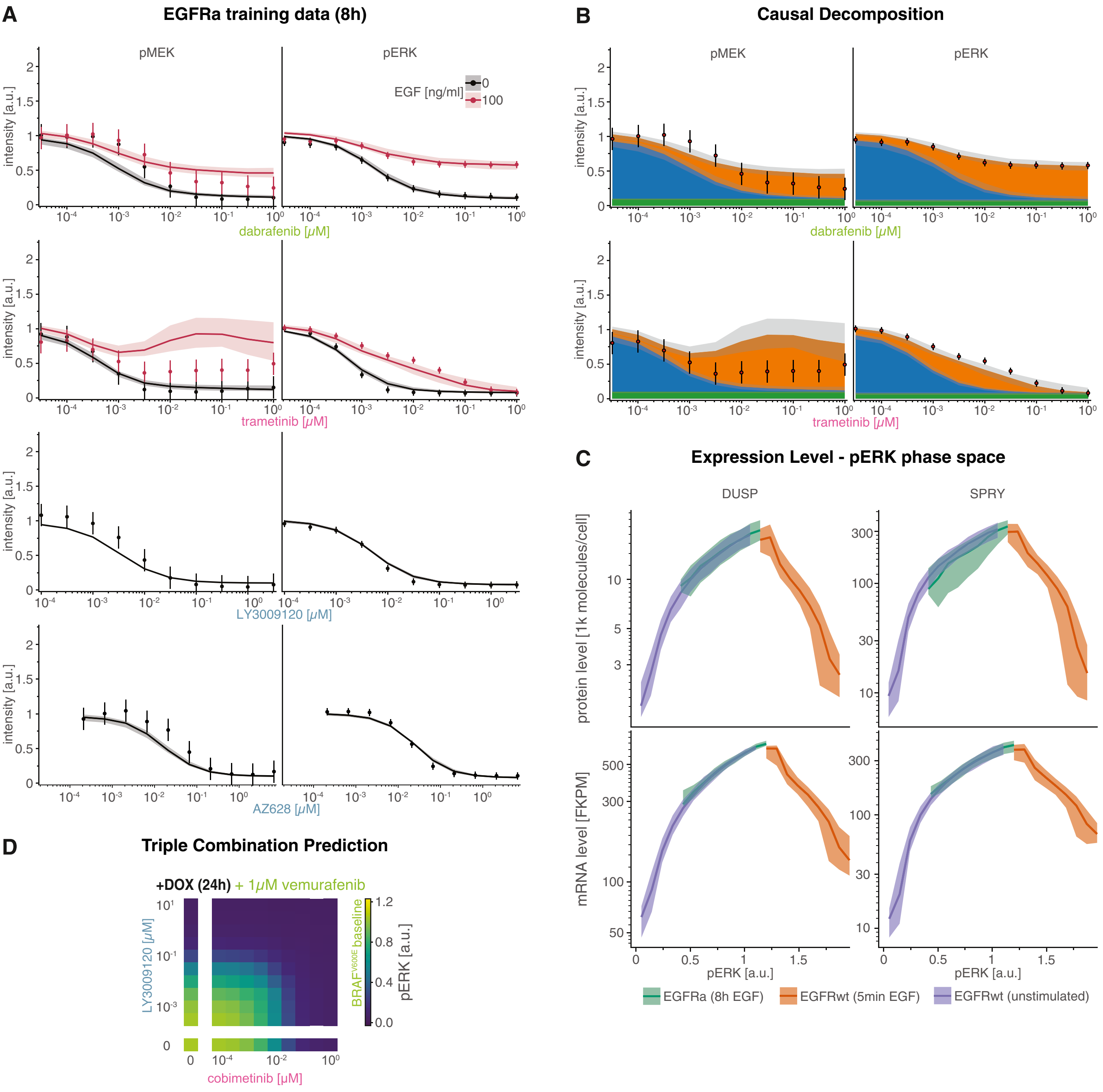

Figure EV5. Additional training data for EGFR upregulation and Causal Decomposition.

Experiments were performed in CRISPRa-EGFR A375 cell lines in 5% FBS medium after 24 h of drug adaptation. EGF stimulation was at 100 ng/ml. Data are shown as point-ranges with average over technical replicates ($n = 2$) as point and estimated standard deviation (over all data points) as line. Data from different experiments (biological replicates) are shown separately. Thick lines or stacked areas show median simulations and shading indicates 80% percentiles over 50 best parameter sets.

A Model simulations and experimental data for pMEK (left) and pERK (right) in EGF stimulated (8 h) and unstimulated conditions.

B Comparison of experimental data and decomposed model simulations at 5 min after EGF stimulation. Simulations are shown as stacked areas with color corresponding to channels (blue: $BRAF^{V600E}$, orange: RAS).

C Relationship between DUSP and SPRY expression levels and ERK phosphorylation levels under different experimental conditions (shown as different colors). pERK levels were binned into 20 equidistant discrete levels.

D Predicted dose response for combinations of LY3009120 and cobimetinib at 1 µM vemurafenib. Simulations were performed for $BRAF^{V600E}$ $NRAS^{Q61K}$ double mutant cells that were adapted to all three drugs.

© 2023 The Authors
