# Supplementary material for: Mechanistic model of MAPK signaling reveals how allostery and rewiring contribute to drug resistance
Source: Mol Syst Biol. 2023 Jan 26;19(2):e10988. doi: 10.15252/msb.202210988 (PMC9912026; doi:10.15252/msb.202210988)
Supplement: Supplementary file 5 [file MSB-19-e10988-s003.pdf]

molecular systems biology

# Mechanistic model of MAPK signaling reveals how allostery and rewiring contribute to drug resistance

Fabian Fröhlich, Luca Gerosa, Jeremy Muhlich, and Peter Sorger
**DOI: 10.15252/msb.202210988**

*Corresponding author(s): Peter Sorger (peter_k_sorger@hms.harvard.edu)*



## Review Timeline:

| | |
|---|---|
| Submission Date: | 17th Feb 22 |
| Editorial Decision: | 31st Mar 22 |
| Appeal Received: | 7th Jun 22 |
| Editorial Decision: | 9th Jun 22 |
| Revision Received: | 10th Aug 22 |
| Editorial Decision: | 11th Oct 22 |
| Revision Received: | 29th Nov 22 |
| Accepted: | 15th Dec 22 |

*Editor: Jingyi Hou*


## Transaction Report:

(Note: With the exception of the correction of typographical or spelling errors that could be a source of ambiguity, letters and reports are not edited. Depending on transfer agreements, referee reports obtained elsewhere may or may not be included in this compilation. Referee reports are anonymous unless the Referee chooses to sign their reports.)

## 1st Editorial Decision 31st Mar 2022

Thank you for submitting your work to Molecular Systems Biology. We have now heard back from the three reviewers who agreed to evaluate your manuscript. As you will see below, the reviewers raise substantial concerns about your work, which unfortunately preclude its publication in Molecular Systems Biology.

The reviewers acknowledge that the general topic of the study is relevant. While Reviewer #3 (whose expertise is in cell signaling and bioengineering with an emphasis on experimentation)is more positive, Reviewers #1 and #2 (who are both experts in MAPK signaling and mathematical modeling) raise substantial concerns about the assumptions and omissions in the model that potentially undermine the main conclusions of the study. Overall, I am afraid that both reviewers express limited enthusiasm and are not convinced of the conclusiveness and overall advance of the study. During our pre-decision cross-commenting process (in which the reviewers are given a chance to make additional comments, including on each other's reports), the reviewers made further comments about the potential shortcomings of the study, which I have included below.

Under these circumstances and given that the concerns raised by the reviewers are substantial, we see no other choice than to return the manuscript with the message that we cannot offer to publish it. In any case, thank you for the opportunity to examine your work. I hope that the points raised in the reports will be helpful to you and that you will not be discouraged from submitting future work to Molecular Systems Biology.

---

Reviewer #1:

The paper by Fabian Froehlich and colleagues 'Mechanistic model of MAPK signaling reveals how allostery and rewiring contribute to drug resistance' tries to obtain a comprehensive, contemporary portrait model of MAPK signaling. It distinguishes itself refreshingly from the large body of bioinformatics papers published by MSB. The paper integrates numerous structural, biochemical, and computational aspects of work on MAPK signaling including feedback regulation, thermodynamics and rule-based approaches. In particular, the authors use multi-model objective calibration to push the envelope in the calibration of large BioNetGen models from the available experimental data. At the same time, the manuscript and model have significant weaknesses which the authors need to address before publication can be recommended. Currently, their model does not generate a precise, quantitative description of MAPK signaling and its adaptive rewiring. Key statements and interpretation of the results should be revisited and clarified, as detailed below. The manuscript needs to be substantially re-worked and re-written to be recommended for publication in MSB.

Major points.
1. Abstract 'We demonstrate the presence of two parallel MAPK (RAF-MEK-ERK kinase) reaction channels in BRAFV600E melanoma cells that are differentially sensitive to RAF and MEK inhibitors. This arises from differences in protein oligomerization and allosteric regulation induced by oncogenic mutations and drug binding. As a result, the RAS-regulated MAPK channel can be active under conditions in which the BRAFV600E-driven channel is fully inhibited'
The lines 456-460 'Experimentally determined pMEK and pERK levels measure the sum of active MAPK kinases generated by oncogenic and chronically active BRAFV600E and by transiently active EGFR (Figure 2B). To decompose these two sources of MAPK activity, we modeled a "RAS reaction channel," which encompasses all reactions initiated by (RAS-GTP)2-RAF2 oligomers, and a "BRAFV600E reaction channel" encompassing all MAPK reactions downstream of the BRAF oncogene.'
- These statements are misleading and too simplistic. They neglect the complexity of the biology they claim to model. The authors must distinguish between a BRAFV600E monomer plus CRAF monomer signaling channel and a RAF dimer/oligomer signaling channel. The latter includes signaling by both BRAFV600E homo-/heterodimers and CRAF homodimers, which are free or bound to RAS-GTP. When RAS-GTP is activated by EGF, BRAFV600E dimerization is enhanced and therefore the total RAF kinase activity increases, even when the BRAFV600E protomer in many dimers is bound to Vemurafenib.
Within defined signaling channels it is difficult to see a place for CRAF monomer signaling in A375 cells. For other cells with heterozygous BRAF mutations (where one allele is WT BRAF and the other is BRAFV600E), the BRAFV600E monomer signaling channel does not account for signaling by WT BRAF and CRAF monomers. It also cannot be included in the so-called RAS reaction channel, which the authors define as exclusively RAF dimer/oligomer signaling. The authors then aimed to extend the model to HT29 cells with a heterozygous BRAFV600E mutation and high EGFR expression. These cells have active BRAF and CRAF monomers. Although signaling by WT BRAF and CRAF monomers has a much smaller output than signaling by BRAFV600E and RAF homo- and heterodimers, it is unclear why the authors introduce a new concept that cannot be generalized, whereas a well-known conceptual separation of RAS/RAF/MEK/ERK signaling into RAF monomer and RAF dimer/oligomer signaling channels would suffice to describe the overall signaling output correctly.
Please remove ambiguous terminology. After all, this is a systems biology paper where using undefined terms will do more harm than good. Systems biology is supposed to deliver precise models of natural biological processes. Thus, terms like 'causal decomposition' seem displaced. The manuscript should be re-written in a way that correctly distinguishes between BRAFV600E, BRAF WT (in heterozygous BRAF mutant cells) plus CRAF monomer signaling and RAF dimer signaling channel that includes dimers containing BRAFV600E. This publication is potentially very important to the field. Therefore, its concepts must be clear and generalizable.

2. The model does not include inhibiting phosphorylations of BRAFV600E and CRAF by ERK. Both BRAF(AA600~E,RBD,mek,raf,rafi) and CRAF(RBD,mek,raf,rafi) do not have inhibitory ERK phosphorylation sites. In the supplementary file "Model Documentation.ipynb" the authors write 'Some of the negative feedback mechanisms that were described in previous studies are omitted in this model, including Erk negative feedback phosphorylations on Mek, and Raf. As the timescale of phosphorylation reactions is in the order of seconds, a high inhibitory potency of these feedback mechanisms should give rise to phospho-Erk pulses on a timescale of seconds. Yet the experimentally observed timescale of phospho-Erk pulses was in the order of minutes, which suggests that it is unlikely that phosphorylations feedbacks are the primary determinant of the pulse shape. This hypothesis is backed by previous studies on phospho-turnover in Egfr mediated signaling. Accordingly we only expect a subtle effect of the negative feedback mechanisms, which would be difficult to be resolve in the model, given that no experimental data on respective phosphorylation sites or Ras-GTP levels were available.'
This statement is simply wrong. There are numerous papers demonstrating the importance of this feedback and the seconds vs, minutes argument has no traction in the face of the published literature. Omission of this feedback is a substantial structural failure of the model. A crucial role of the inhibitory feedback phosphorylation of BRAF, BRAF mutants and CRAF on multiple sites by ERK is well documented (see, e.g., doi:10.1016/j.molcel.2004.11.055; doi:10.1128/MCB.00569-09). In fact, ERK phosphorylation of RAFs controls both RAS-GTP-RAF binding and RAF dimerization, two key processes of physiological and pathological activation of RAF kinases. Its omission led to several incorrect statements about signaling by BRAF/CRAF monomers (which the authors termed as BRAFV600E reaction channel, that is a superficial term, as mentioned above), as for instance independence of this signaling from the pERK level. Inhibitory SOS1 phosphorylation by pERK, which disrupts Grb2-SOS1, cannot substitute pERK-mediated feedback to RAFs in a model. It must be incorporated in the model.
This brings me to another important point. It seems that critical model features are hidden in supplementary Python files. I think scientific integrity demands that critical model features are fully disclosed and described in the main text and transparently presented in the Methods section.

3. Lines 212 - 216. 'FDA approved MEK inhibitors such as cobimetinib, trametinib and binimetinib, are type III non-ATP competitive (allosteric) inhibitors that lock the MEK kinase in a catalytically inactive state, limit movement of the activation loop, and decrease phosphorylation by RAF (Wu & Park, 2015). These MEK inhibitors are more potent at preventing ERK activation by BRAFV600E/K than by RAF acting downstream of mutant RAS (Lito et al, 2014; Hatzivassiliou et al, 2013) or RTKs (Gerosa et al, 2020).'

- This stamen is incorrect. MEK inhibitors dramatically differ in terms of their ability to decrease MEK phosphorylation. Trametinib strongly suppresses MEK phosphorylation, its suppression by cobimetinib is significantly weaker, and binimetinib suppresses it even weaker (doi:10.1158/2159-8290.CD-20-1351). Moreover, in RAS-mutant cells cobimetinib increases MEK phosphorylation (doi:10.1038/nature12441). Diverse MEK inhibitors have different effects and properties. Their IC50 values for BRAFV600E-mutant and RAS-mutant cell lines differ by factors from 5 to 100, depending on their action mode (doi:10.1038/nature12441). Therefore and importantly, these inhibitors cannot be modelled in the same way. The model needs to be tailored to describe cobimetinib mode of action, and it must be adjusted to describe other MEK inhibitors.

4. Section title: 'Causal Decomposition untangles Intertwined BRAFV600E and RAS Driven Signaling.'
-This section leads the reader to wrong conclusions. BRAFV600E also makes homo- and heterodimers, and all effects described in this section can be explained by dimerization/oligomerization of mutant BRAFV600E.

5. Lines 476-484. 'The value of causal decomposition was illustrated when we investigated the observed increase in pERK levels in the BRAFV600E channel following EGF addition (blue, Figure 2E). This was unexpected, since, in MARM2.0, EGF only activates the RAS channel. We surmised that activation of the BRAFV600E channel might arise from retroactivity' ... 'Thus, activation of the RAS channel can activate the BRAFV600E channel by reducing the rate of DUSP dependent pERK dephosphorylation.'
- A. The two signaling channels are merely signaling by BRAFV600 and CRAF monomers (termed the BRAFV600E channel)

and by all RAF homo/hetero-dimers (termed the RAS channel).
- B. A biologist reading these claims will be puzzled because EGF activates not only RAS signaling but also BRAFV600E signaling via RAS-GTP-facilitated formation of BRAFV600E homo- and hetero-dimers. This again requires replacing two channels by a clear distinction between signaling by only BRAFV600E monomers vs signaling by RAF dimers/oligomers.

6. Lines 484 - 490 'A second example of causal decomposition involved experimental data showing that pMEK levels remain roughly constant over a 105-fold range of RAFi concentrations (as monitored at the 5-minute peak of an EGF-induced pulse, Figure 2F left).'
- I presume the authors mean Fig. 2E left.
'Causal decomposition showed that this unexpected behavior arose from a steady reduction in the activity of the BRAFV600E channel (blue) with increasing RAFi and a simultaneous and offsetting increase in signaling in the RAS channel (orange). This was true of all 3 RAFi and 5 MEKi tested (Figure S3) and represents a classic case of pathway rewiring that is obscured at the level of total MAPK activity.'
- The dose responses presented in Fig. S3 for different MEKi look similar and do not correlate with the reported different dose responses for different MEKi measured in many BRAFV600E cell types (e.g. doi: 10.1038/nature12441; doi:10.1158/2159-8290.CD-20-1351). The figure legend needs to provide more details. Currently, it fails to provide details about the time exposure to the inhibitors and conditions, e.g. if serum was present or absent in the media.

7. 'The resulting isobolograms had a convex shape (Figure 6E) with minimal drug interaction by Bliss (Figure 6F) or HSA criteria (Figure 6G). This differs from what was observed with pulsatile RTK activation (Figure 5C, D bottom panels) and suggests that drug interactions in the case of pulsatile signaling were only possible due to time scale separation between drug adaption and direct drug action.'
- In Figs. 5 - 7 isobolograms are given on log-log scales. On log-log scale, a straight line that represents drug additivity will be convex, making it impossible to distinguish drug antagonism from additivity. The Bliss criterion is a weak criteria used only for a preliminary screening of thousands of drug interactions. For instance, the Bliss criterion might show that a drug is synergistic or antagonistic with itself, although two doses of the same drug would merely be additive (see, e.g., doi: 10.1038/nrmicro2133). All isobolograms must be shown on linear scales where they would clearly show the areas of drug additivity, synergy and antagonism (PMID: 7568331).

8. Lines 741-743. 'To investigate whether MARM2.0 could predict the responses of BRAFV600E colorectal cancers to RAFi, we collected data from HT29 cells, which carry a BRAFV600E mutation and have high EGFR expression (similar to A375 EGFR-CRISPRa cells).
- Fig. 9 only shows simulations. Experimental data need to be obtained and imposed on simulated responses, as in other figures.

9. HT29 cells (unlike A375 cells) have a heterozygous BRAFV600E mutation, bearing a WT BRAF allele. Does A375 model describes cells with homo- and heterozygous BRAF mutations?

Minor points.
1. Figure legends must provide more details. Currently these legends are unhelpful. For instance, the legends to Fig. S2B,F do not tell if drug-naive cells were also kept for 24 hr after EGF addition. Additional clarity is needed for legends to Fig. S3 and others. Figures with isobolograms lack doses on the axes, and must be generated on linear scale.

2. Lines 307-308 'MARM2.0 nonetheless has over 15,000 biochemical reactions' and Lines 356-357 ... 'generated >2,200 molecular species and >30,000 biochemical reactions'
- these two statements must be reconciled, because the numbers are inconsistent.

3. Line 303: 'panRAFi'
- The term 'pan' refers to the ability of these RAFi bind both BRAFV600E and CRAF. However, Vemurafenib also binds both RAF isoforms with similar affinities (https://www.selleckchem.com/products/PLX-4032.html). Most importantly, these RAFi are Type II RAFi, whereas Vem and Dabrafenib are type I1/2 RAFi, which should be clarified in this piece.

4. Why do you assume that the decomposition into two different signaling fluxes can be termed causal?

5. Fig.2B must show RAS and indicate whether RAS facilitates the formation of BRAFV600E homo- and hetero-dimers.

6. Lines 531- 532 'The ratio of input to output signals in a network (the gain) is a fundamental property of a signal transduction system that can be used quantify rewiring.'
- Gain is the ratio of output to input signals.

7. A cartoon in Fig. 4A is misleading. RAS signals to MEK via RAF isoforms, monomers and dimers.

8. The Fig. 4A cartoon contradicts to the following sentence: 'We found that drug adaptation to RAFi and MEKi had a similar impact on the first step (R1) of both reaction channels (Figure 4C, top panels).

- Both channels, BRAF600E and CRAF monomers and all RAF dimers, signal to MEK, thus MEK must be explicitly shown for both channels'. In fact, the step R1 is shown only for the EGFR/RAS channel.

9. Line 572. 'Thus, even at 10μM, the highest vemurafenib concentration tested, and a value well above the clinically useful range, ...'
- Clinically, vemurafenib concentrations as high as 100μM in blood were reported (doi: 10.1093/annonc/mdv189).

10. 'Moreover, estimated ranges for $\Delta\Delta G$ were similar for the four other type I1/2 RAFi drugs we tested (Figure S1, S4).'
- What are these I1/2 RAFi drugs? In the experiments shown only Vemurafenib was tested.

11. 'In the absence of exogenous growth factors (Figure 5A(i)), we predicted a monotonic decrease in pERK levels'
- Was serum present in the media as it is normally present?

12. Line 627. '...recapitulating the non-monotonic response to MEKi in Figure 2A, in which pERK levels first rose and then fell with increasing drug concentration.'
- Fig. 2A shows the kinetics/time-course of the MEKi response. How can the steady-state isobolograms relate to details of the kinetics?

13. Lines 200-202. 'Thus, the structural differences between monomers and dimers (rather than differences in the ATP binding pocket) are the basis of the selectivity of clinically approved RAF inhibitors for cells transformed by BRAF mutant kinases.'
- There is other critical structural difference between RAF inhibitors. DFG-IN, αC-IN RAF inhibitors promote RAF dimerization much stronger than DFG-OUT, αC-IN RAF inhibitors (doi: 10.1016/j.ccell.2016.06.024), and this might be a reason why DFG-IN, αC-IN RAF inhibitors are no longer being developed.

14. The exact formulas for different synergy scores need to be presented in Methods, because different authors use slightly different equations for the same Bliss criteria, etc.

15. Model assumptions must be formulated in the main text and Methods, and not only in supplemental Python files.

Reviewer #2:

Fröhlich: In the present manuscript, Fröhlich and coauthors describe the development, parameterization and application of a detailed MAPK signal transduction model. The model is an extension of a previously published model that contains a bit more details (mainly compartmentalization, receptor endocytosis), and is trained on some additional data. Overall, it is conceptually similar to the previous model. As they write: "(the previous paper did) not involve any model analysis. Such analysis is the focus of the current paper and its updated model". Large parts of the results describe details of the model construction (which I think is carefully done) and optimization of the model calibration procedure that helps to reduce runtime for calibration. Though this is very technically, I think this is an important part and really the strength of the paper (though maybe more for a technical journal). Then the authors introduce what they call "causal decomposition". By introducing additional variables ("labels"), they can then trace back how much of the pERK signal derives from WT RAF / RAS and mutant RAF. I think this is the most important conceptual advance of the manuscript. By doing so, they provide a framework to analyze the dynamics of the very complex and large model in rather simple terms, i.e. they provide a narrative of how blocking mutant RAF enables RAS-mediated activation of MAPK signaling. However, mechanistically this has been known for a while and is also discussed in their previous paper that included the MAPK model. Thus, while it is a technical advance, its biological novelty is not really clear, and the quantitative validation of the model (which would be the advance over previous studies) is not really done. They then use the model to evaluate dose-response curves of multiple inhibitors and cell lines. While this is generally nicely done and models are mostly in good agreement with the response curves, it merely shows the existence of a feedback in the system, but a real and direct quantitation of the contributions is missing.

My main concern is that the presented analysis suggests a very quantitative framework to dissect contributions of the channels, and also the detailed models of signaling upstream of RAF suggests that the mechanisms are very precisely covered. Yet, the models do not contain some basic important mechanisms that may also contribute (or maybe even contribute much more), which is e.g. a very strong MAPK feedback to RAF, and obviously lack data to really quantitatively dissect contribution of different feedbacks. Thus, it remains unclear (apart from the technical advances) why and how such a detailed model contributes to our understanding or MAPK signaling or resistance.

Thus, while I applaud the authors for their technical rigor and the enormous amount of data and work that went into model calibration, I don't really see the major advance in our understanding that arises from this model.

Minor: In part, the manuscript uses very bold language that the authors should avoid given the level of evidence. E.g:

"to model the precise meachanism" (abstract). Isn't it rather one plausible mechanism?
"powerful constraints" - what is this exactly? What does powerful mean here?

Reviewer #3:

Frohlich et al. present an interesting manuscript that details an extension of the original MARM model previously published by the authors. MARM 2.0 is a thermodynamic model that leverages differences in free energies to implement a rule-based system that captures many more complex interactions than would be practical with simple ODE modeling. This new model allows a more detailed dissection of signal rewiring in BRAF V600E melanoma in the presence of single-agent or combination therapies, and leads to several non-intuitive insights that were verified experimentally. Among other highlights, the authors use their model to trace signal flow through two distinct but parallel channels of Ras-to-Erk signaling, and measure how the flux ratio changes as a function of drug dose. The model also allowed the calculation of the gain from one node to the next at every step in the pathway, and found instances of increased gain at nodes that correlate with network nodes observed to be activated during resistance. The collective transfer functions between nodes, together, could predict the signaling response to combinations of BRAF and MEK inhibitors. Finally, the model also predicted responses to drug combinations in the presence of EGFR overexpression and additional resistance-associated mutations.

Although I am not well-positioned to evaluate the details of the modeling or its implementation, the model addresses an interesting and important biological question of signal rewiring in BRAF V600E melanoma, and allows a detailed and testable breakdown of how signal flux is altered during drug treatment. Moreover the paper is thorough and is written with a high degree of clarity, which is challenging for such a complex topic and model. This work will allow better prediction of how targeted therapies rewire signaling in drug adapted cancer cells, which will expedite the process of combinatorial therapies to combat cancer resistance. In addition the model seems to represent several methodological advances that should be broadly applicable to various modeling scenarios. Thus I recommend publication with only a few minor comments below:

-Although there are callouts for boxes 1,2,and 3, I didn't see figures for Boxes 2 and 3 in the manuscript
-All experimental validations are performed with 100 ng/mL EGF, which is a very strong, superphysiological signal. Could the authors comment on how the model would perform at different stimulus concentrations?
-in some cases, figures are called out of order (eg Figure 1B is called out after 1C and 1D)
-line 428: the term "wall-time" wasn't familiar to me
-in figure 3B, it was difficult to see the differences between brown and purple shading of the arrows.
-starting on line 514, the switching of cyclic to acyclic networks is interesting. It would help if the authors would clarify why this is important. Is this to more easily be able to understand the causality in the network? Perhaps a sentence or two would help motivate the approach.
-Figure 1B - the title is "oligomerization", but it seems this is better described as "binding". Oligomerization to me implies a higher-order interaction.
-Fig 2F: legend says that data are represented as point ranges, which I understand to mean that the bars span the min and max measurements. But what are the points? And sometimes there are 2 points and sometimes one point. Same in Fig 6B. In general, the authors could include more details on data representation in the legends.
-Figure 4C: it took me a while to understand what the 3 rows of plots represented. Would be clearer if they were labelled "R1/B1", "R2/B2" or similar.
Figure 4D: the diagram is a nice way to summarize the data, but I don't think I understood it. Why is pEGFR becoming more transparent with added vemurafenib/MEKi? Why is BRAF (blue circle) not becoming more transparent with increasing vemurafenib?
Figure 5G: it would help clarify the comparison between 5A and 5G is the "L"
shape in 5G were outlined, as in 5A.
Figure 8D: It took me some time to understand here that NRAS is dox-inducible. This could be clarified in the figure or in the caption
Figure 8D, top right. In many figures, its not clear what the heatmap is showing. I think here it is pErk, but this should be made clear in every figure and caption.
-Figure S7 is called out in at least 2 different places (line 701 and line 731), but neither description fits what is in Figure S7, as far as I can tell (also the current S7 is labelled as S5)

-------------------------------------
***Referee Cross-commenting***

Reviewer #3:
Reviewers 2 and 3 bring up many strong points. In my view, the model appears to be a big technical achievement, and would warrant publication largely on these grounds. Although there is a lack of validation of a deep conceptual advance, as Rev. 2 points out, there are interesting predictions made that can shape the way we think about signal rewiring in this cancer system. Rev. 1 raises many important technical discrepancies or omissions/assumptions made by the model. Of course all models are by their nature simplifications, even ones as complex as the ones presented. In my view the authors could be reasonably expected to respond to these critiques and either better justify their assumptions, or alter their model to accommodate. I also agree that, given the complexity, a general increase in details is called for.

Reviewer #2:
I agree that the manuscript would be a big technical achievement - however, the previous version of the model is already published in the cell systems paper which is hardly different from the one reported here. Actually, the current model lacks a very important component (ERK to RAF feedback, see comment 2 of reviewer 1) that the previous model had included, so I don't see it as a major way forward but rather sideward.

Reviewer #1:
A major shortcoming of the current submission is a misleading and too simplistic division of RTK to ERK signaling into a BRAFV600E monomer signaling channel and a RAS signaling channel. Neither of these two channels account for signaling by WT BRAF (present in many BRAF mutant cells) monomers and CRAF monomers. It is also well known that BRAFV600E signaling is dramatically enhanced with increasing RAS-GTP levels because of the corresponding increase in BRAFV600E dimerization homo and hetero-dimers. Another critical omission is the lack of ERK inhibitory feedback to RAF isoforms, including BRAFV600E, which is known to play a crucial role in resistance to RAF inhibitors. If the authors fix their model's flaws and correct the ambiguous and improper terminology of two reaction channels, the manuscript can be published in MSB.

** As a service to authors, EMBO Press offers the possibility to directly transfer declined manuscripts to another EMBO Press title or to the open access journal Life Science Alliance launched in partnership between EMBO Press, Rockefeller University Press and Cold Spring Harbor Laboratory Press. The full manuscript and if applicable, reviewers' reports, are automatically sent to the receiving journal to allow for fast handling and a prompt decision on your manuscript. For more details of this service, and to transfer your manuscript please click on Link Not Available. **

**Detailed Response to Review**
**MSB-2022-10988**
Fröhlich, Sorger et al *Mechanistic model of MAPK signaling reveals how allostery and rewiring contribute to drug resistance*

Below, we provide a detailed point-by-point response that addresses all of the concerns raised by the reviewers. Reviewers comments is provided in boxes with yellow shading, our responses are in boxes with white shading. We provide both a “clean” and redlined version of our revised manuscript.

## Overview of the Response

We thank the reviewers for their detailed feedback on our manuscript. We have addressed all of their comments with changes to the model, analysis, figures and text.

We found *all* of the reviewer’s comments relevant and helpful. Because the reviews were so thorough, it may have appeared that our interpretations and conclusions are very different from their own, but we do not think this is the case. There does not exist a standardized vocabulary for describing the functional properties of dynamical biochemical models, structure-based models in particular, and it is therefore not surprising that disagreement about language arises. We have switched to their suggested terminology in some cases and in others explained our language more carefully.

The most important change in the revision is that we have now extended our model (and subsequent training and analysis) by adding the feedback mechanism whose admission concerned Review 1 (and the other reviewers in the cross-cutting discussion). This mechanism involves the direct inhibitory action of ERK on RAF. This interaction was also missing from the MARM1.0 model described in Gerosa et al 2021 (that is, we did not newly omit it in version 2.0 as Reviewer 1 suggested). Addition of ERK to RAF negative regulation did not substantially change model fit or alter any of our major conclusions. We nonetheless agree that this mechanism should have been included in our paper.

We have also been more careful about the concept of channel decomposition. Our understanding of these results seems congruent with the reviewer’s but we do believe that our description of functional “reaction channels” that assemble dynamically using exchangeable components is a useful conceptual framing. Our goal in this case is to bridge the sophisticated understanding that reviewers bring to the problem with the more common and static concept of pathway “wiring” and “rewiring.” With the substantial re-writes and more complete explanations now in the manuscript we hope that the reviewers will indulge our attempts to make the conclusions of the current study easier to understand. At the same time, we are happy to implement any final textual revisions that might arise on re-review.

# Reviewer #1

**Reviewer 1:**
The paper by Fabian Froehlich and colleagues 'Mechanistic model of MAPK signaling reveals how allostery and rewiring contribute to drug resistance' tries to obtain a comprehensive, contemporary portrait model of MAPK signaling. It distinguishes itself refreshingly from the large body of bioinformatics papers published by MSB. The paper integrates numerous structural, biochemical, and computational aspects of work on MAPK signaling including feedback regulation, thermodynamics and rule-based approaches. In particular, the authors use multi-model objective calibration to push the envelope in the calibration of large BioNetGen models from the available experimental data. At the same time, the manuscript and model have significant weaknesses which the authors need to address before publication can be recommended. Currently, their model does not generate a precise, quantitative description of MAPK signaling and its adaptive rewiring. Key statements and interpretation of the results should be revisited and clarified, as detailed below. The manuscript needs to be substantially re-worked and re-written to be recommended for publication in MSB.

**Response:** We thank the reviewer for the positive evaluation and we believe that we have now responded to all of the reviewers concerns about model structure and interpretation.

## Major points

**Reviewer 1 (Major Point 1B):**
Within defined signaling channels it is difficult to see a place for CRAF monomer signaling in A375 cells. For other cells with heterozygous BRAF mutations (where one allele is WT BRAF and the other is BRAFV600E), the BRAFV600E monomer signaling channel does not account for signaling by WT BRAF and CRAF monomers. It also cannot be included in the so-called RAS reaction channel, which the authors define as exclusively RAF dimer/oligomer signaling. The authors then aimed to extend the model to HT29 cells with a heterozygous BRAFV600E mutation and high EGFR expression. These cells have active BRAF and CRAF monomers. Although signaling by WT BRAF and CRAF monomers has a much smaller output than signaling by BRAFV600E and RAF homo- and heterodimers, it is unclear why the authors introduce a new concept that cannot be generalized, whereas a well-known conceptual separation of RAS/RAF/MEK/ERK signaling into RAF monomer and RAF dimer/oligomer signaling channels would suffice to describe the overall signaling output correctly.

**Response:**
We agree with the reviewer that CRAF monomer and WT BRAF signaling is not relevant for MAPK signaling in A375 cells that carry a homozygous BRAFV600E mutation. As only data from A375 cells was used for model calibration, the respective reactions were not included in the model. However, the rule-based model formulation of MARM2 makes it straightforward to include such reactions when modeling other cell lines (assuming new calibration data is available). Similarly, channel decomposition could easily be extended accordingly, potentially including additional channels. Based on the reviewer's comments, we will look more carefully at heterozygous melanoma cell lines in the future.

We thank the reviewer for mentioning that the BRAFV600E mutation in HT29 cells is heterozygous, since this was indeed *not* mentioned in our original (submitted) manuscript. To address the reviewers concern, we have updated the section on HT29 cells to reflect the fact that it carries a heterozygous BRAFV600E mutation and that we don't account for heterozygosity when making predictions. Moreoever, because we also use a single source of calibration data, we ignore the many other differences between colorectal (HT29) and melanoma (A375) cell lines that would need to be accounted for before we can conclude that we fully understand the differences between BRAF-mutant melanoma– in which RAF/MEK inhibition is therapeutically useful – and colorectal cancer, in which it is not. However, the fact that we were able to make accurate predictions without accounting only for differences EGFR expression level suggests that omission of WT BRAF signaling was not a critical weakness. Instead, EGFR overexpression itself emerges as a critical and sufficient (albeit not a necessary) explanation for the difference between colon and melanoma cells. We have re-written this section of the manuscript to better convey this conclusion,

**Reviewer 1 (Major Point 1C):**
Please remove ambiguous terminology. After all, this is a systems biology paper where using undefined terms will do more harm than good. Systems biology is supposed to deliver precise models of natural biological processes. Thus, terms like 'causal decomposition' seem displaced. The manuscript should be re-written in a way that correctly distinguishes between BRAFV600E, BRAF WT (in heterozygous BRAF mutant cells) plus CRAF monomer signaling and RAF dimer signaling channel that includes dimers containing BRAFV600E. This publication is potentially very important to the field. Therefore, its concepts must be clear and generalizable.

**Response:**
We certainly agree with the reviewer on this point but are not entirely sure where we have erred in our description of BRAF variants (other than in the case of HT29 cells; see above). We have scoured the paper and figures to catch ambiguities to the best of our ability.

As the reviewer appreciates, the monomer/dimer dichotomy described in the biochemical framework of this paper (and previous work by Kohlodenko), oncogenic monomers can signal in the absence of upstream dimerization but are drug sensitive. Dimers are substantially more drug resistant, This paper, and our previously experimental work (Gerosa et al. 2021) strongly implicate growth factor receptors in the formation of these dimers – to our knowledge this has not been explicitly explored previously. Starting from the perspective of the origin of the MAPK signal (an oncogene or a receptor) we then attempt to determine the properties of the downstream effectors. The concept of a reaction channels comprising transiently associated components provide a general conceptual framework to analyze signaling flow in an intertwined signaling network using overlapping molecular species and it specifically intended to formalize the concept of "rewiring" (which has several thousand citations in PubMed).

We chose the adjective "causal" based on the upstream reaction pattern that triggers a specific reaction in the model, i.e. the upstream causal event. The same "causal" notation was used in the context of causal tracing for agent-based models, which inspired the approach we described. Fontana, Faeder and others active in rules based modeling frequently use causal in this sense it seems appropriate even though it is a concept from agent-based rather than ODE simulation. We believe that the definition makes our findings more generalizable, as it is straightforward to apply to any rule-based model, including in settings where the model accounts for MEK phosphorylation by RAF WT monomers or BRAF WT dimers.

To address the reviewers concern, we have updated our description of signaling channels to include a more detailed explanation of why we chose `causal` as an adjective. Moreover, we are now contrasting the reaction channel concept to the more canonical, biochemical distinction of RAF monomer and dimer signaling.

**Reviewer 1 (Major Point 2A):**
2. The model does not include inhibiting phosphorylations of BRAFV600E and CRAF by ERK. Both BRAF(AA600~E,RBD,mek,raf,rafi) and CRAF(RBD,mek,raf,rafi) do not have inhibitory ERK phosphorylation sites. In the supplementary file "Model Documentation.ipynb" the authors write 'Some of the negative feedback mechanisms that were described in previous studies are omitted in this model, including Erk negative feedback phosphorylations on Mek, and Raf. As the timescale of phosphorylation reactions is in the order of seconds, a high inhibitory potency of these feedback mechanisms should give rise to phospho-Erk pulses on a timescale of seconds. Yet the experimentally observed timescale of phospho-Erk pulses was in the order of minutes, which suggests that it is unlikely that phosphorylations feedbacks are the primary determinant of the pulse shape. This hypothesis is backed by previous studies on phospho-turnover in Egfr mediated signaling. Accordingly we only expect a subtle effect of the negative feedback mechanisms, which would be difficult to be resolve in the model, given that no experimental data on respective phosphorylation sites or Ras-GTP levels were available.'
This statement is simply wrong. There are numerous papers demonstrating the importance of this feedback and the seconds vs, minutes argument has no traction in the face of the published literature. Omission of this feedback is a substantial structural failure of the model. A crucial role of the inhibitory feedback phosphorylation of BRAF, BRAF mutants and CRAF on multiple sites by ERK is well documented (see, e.g., doi:10.1016/j.molcel.2004.11.055; doi:10.1128/MCB.00569-09). In fact, ERK phosphorylation of RAFs controls both RAS-GTP-RAF binding and RAF dimerization, two key processes of physiological and pathological activation of RAF kinases. Its omission led to several incorrect statements about signaling by BRAF/CRAF monomers (which the authors termed as BRAFV600E reaction channel, that is a superficial term, as mentioned above), as for instance independence of this signaling from the pERK level. Inhibitory SOS1 phosphorylation by pERK, which disrupts Grb2-SOS1, cannot substitute pERK-mediated feedback to RAFs in a model. It must be incorporated in the model.

**Response:**
We apologize for this omission and have now included the requested negative regulatory mechanisms throughout the paper . As described in the overview to this response, the results of the simulations and analysis are largely unchanged and our manuscript's conclusions stand. However, the quality of fit is improved in the context of A375 cells with CRISPRa- amplified EGFR levels, so we are happy that the reviewer suggested this change.

One feature of our model that distinguishes it from previous models of MAPK signaling is that MARM2.0 assigns a very important role to transcriptionally-mediated negative regulation mediated by DUSP phosphatases and Sprouty. There is extensive evidence in the literature that these forms of negative regulation are important in both normal and tumor cells.

One uncertainty that neither previous genetic studies, our model, or work by Kholodenko and/or Wiley are able to fully resolve is the relative importance of direct negative feedback within the MAPK pathway and more indirect DUSP/Sprouty-mediated negative feedback. Genetic analysis is complicated by redundancy in the sites of protein phosphorylation and the multiplicity of phosphatases, Models are confounded by non-identifiability. It is likely that these various forms of feedback compensate for each both in knockout cells and in wt and counter-factual computational simulations.

**Reviewer 1 (Major Point 2B):**
This brings me to another important point. It seems that critical model features are hidden in supplementary Python files. I think scientific integrity demands that critical model features are fully disclosed and described in the main text and transparently presented in the Methods section.

**Response:**
We agree that we erred in not mentioning the model's Python notebook in the main text (i.e., in the description of the supplementary material), We have = revised the text to make the existence of the key document more obvious,

At the same time we are saddened to hear that the reviewer has interpreted our extensive documentation of the model as an attempt to hide crucial information, as our intent was quite the opposite. At the moment, the main text and the methods section seem very long to us, and we did not think that additional text would be helpful. Moreover, we think that providing detailed model documentation in the supplementary material using accepted interchange languages strategy is both common in the field and likely to lead to more effective exchange of information. We will of course deposit our model in recognized repositories, as well as in the supplements.

We speculate that the reviewer is most concerned about the failure to include the direct feedback of ERK on BRAF/CRAF. As described above, we have now rectified this.

**Reviewer 1 (Major Point 3):**
3. Lines 212 - 216. 'FDA approved MEK inhibitors such as cobimetinib, trametinib and binimetinib, are type III non-ATP competitive (allosteric) inhibitors that lock the MEK kinase in a catalytically inactive state, limit movement of the activation loop, and decrease phosphorylation by RAF (Wu & Park, 2015). These MEK inhibitors are more potent at preventing ERK activation by BRAFV600E/K than by RAF acting downstream of mutant RAS (Lito et al, 2014; Hatzivassiliou et al, 2013) or RTKs (Gerosa et al, 2020).'
- This stamen is incorrect. MEK inhibitors dramatically differ in terms of their ability to decrease MEK phosphorylation. Trametinib strongly suppresses MEK phosphorylation, its suppression by cobimetinib is significantly weaker, and binimetinib suppresses it even weaker (doi:10.1158/2159-8290.CD-20-1351). Moreover, in RAS-mutant cells cobimetinib increases MEK phosphorylation (doi:10.1038/nature12441). Diverse MEK inhibitors have different effects and properties. Their IC50 values for BRAFV600E-mutant and RAS-mutant cell lines differ by factors from 5 to 100, depending on their action mode (doi:10.1038/nature12441). Therefore and importantly, these inhibitors cannot be modelled in the same way. The model needs to be tailored to describe cobimetinib mode of action, and it must be adjusted to describe other MEK inhibitors

**Response:**
We appreciate and generally agree with the reviewers comment that different MEK inhibitors have different modes of action. In fact, the mechanisms mentioned by the reviewer are all described in our manuscript in the sentence following the sentence the reviewer cites. However, our treatment of MEK inhibitors derives not from cell line

studies the reviewer cites (which span a lot of lines and types of assays) but rather from extensive structural and enzymatic studies.

Accordingly, we included parameters that describe modulated phosphorylation rates and decreased affinity for all MEKi and estimated separate values for those parameters for every MEK inhibitor. This allowed the model to learn inhibitor specific modes of action, which we discuss in the section “MAPK Signaling is rewired by Drug Adaptation and Direct Inhibition”. We have now updated the text in the box to explicitly state that modes of action *are* inhibitor specific – precisely as the reviewer specifies.

Despite the known differences in mode of MEKi action, the net result is a higher potency for BRAFV600E compared to RAS-WT signaling for most MEKis considered in this study, which is supported by data from us and many others, including papers cited by the reviewer. However, as the reviewer correctly pointed out, doi:10.1038/nature12441 specifically mentions AZD6244 (Selumetinib) as having lower potency towards KRAS-mutant compared to BRAFV600E cells and we have updated our statement accordingly. This slightly deviates from our data on KRAS-WT mediated RAF dimer signaling, suggesting that KRAS mutations also contribute to allosteric interactions. However, confirming this will require additional work.

**Reviewer 1 (Major Point 4):**
4. Section title: 'Causal Decomposition untangles Intertwined BRAFV600E and RAS Driven Signaling.' -This section leads the reader to wrong conclusions. BRAFV600E also makes homo- and heterodimers, and all effects described in this section can be explained by dimerization/oligomerization of mutant BRAFV600E.

**Response:**
We believe that this comment is that the result of a misunderstanding of our definition of reaction channels and hope that the rewrites described in point 1 above resolve this issue. However, we remain convinced that the analysis in Figures S1F and S6C demonstrate the correctness of our conclusions. If the reviewer identifies additional flaws in the analysis or we misunderstood the reviewer’s comments, we are happy to revise this section again.

**Reviewer 1 (Major Point 5):**
5. Lines 476- 484. 'The value of causal decomposition was illustrated when we investigated the observed increase in pERK levels in the BRAFV600E channel following EGF addition (blue, Figure 2E). This was unexpected, since, in MARM2.0, EGF only activates the RAS channel. We surmised that activation of the BRAFV600E channel might arise from retroactivity' ... 'Thus, activation of the RAS channel can activate the BRAFV600E channel by reducing the rate of DUSP dependent pERK dephosphorylation.'
- A. The two signaling channels are merely signaling by BRAFV600 and CRAF monomers (termed the BRAFV600E channel) and by all RAF homo/hetero-dimers (termed the RAS channel).
- B. A biologist reading these claims will be puzzled because EGF activates not only RAS signaling but also BRAFV600E signaling via RAS-GTP-facilitated formation of BRAFV600E homo- and hetero-dimers. This again requires replacing two channels by a clear distinction between signaling by only BRAFV600E monomers vs signaling by RAF dimers/oligomers.

**Response:**
Please see the response above – we think there is a misunderstanding here. It is certainly true that BRAFV600E can participate in homo and heterodimers, but in our model EGF will only directly activate the RAS channel (which includes RAS activated BRAFV600E dimers). We have rephrased the text to reflect that EGF does not *activate* the BRAFV600E channel, but *amplifies* signaling by decreasing phosphorylation. We think this is an interesting conclusion that derives directly from model analysis.

**Reviewer 1 (Major Point 6):**
6. Lines 484 - 490 'A second example of causal decomposition involved experimental data showing that pMEK levels remain roughly constant over a 105-fold range of RAFi concentrations (as monitored at the 5-minute peak of an EGF-induced pulse, Figure 2F left).'
- I presume the authors mean Fig. 2E left.

'Causal decomposition showed that this unexpected behavior arose from a steady reduction in the activity of the BRAFV600E channel (blue) with increasing RAFi and a simultaneous and offsetting increase in signaling in the RAS channel (orange). This was true of all 3 RAFi and 5 MEKi tested (Figure S3) and represents a classic case of pathway rewiring that is obscured at the level of total MAPK activity.'
- The dose responses presented in Fig. S3 for different MEKi look similar and do not correlate with the reported different dose responses for different MEKi measured in many BRAFV600E cell types (e.g. doi: 10.1038/nature12441; doi:10.1158/2159-8290.CD-20-1351). The figure legend needs to provide more details. Currently, it fails to provide details about the time exposure to the inhibitors and conditions, e.g. if serum was present or absent in the media.

**Response:**
The reviewer is correct in his assessment that we were referring to the wrong figure panel and we have updated the text accordingly. We have also updated the figures and legends to include the additional data requested by the reviewer, including by describing the serum concentration and exposure times to drugs. For the vast majority of experiments, we used 5% FBS mediums, 100 ng/ml EGF stimulation and 24h drug adaptation prior to EGF stimulation.

The data presented in 10.1038/nature12441 concerns the three type II MEK inhibitors GDC-0973 (cobimetinib), GDC-0623 (G-868) and G-573, of which we only examine cobimetinib (because the other molecules did not lead to therapeutic drugs). For cobimetinib, the authors consistently observed higher potency towards BRAFV600E cells lines compared to KRAS mutant lines, which is consistent with our findings in RAS WT and NRAS mutant cell lines. The data presented in doi:10.1158/2159-8290.CD-20-1351 concerns the five type II MEK inhibitors trametinib, TAK 733, PD0325901, cobimetinib, CH5126766 and selumetinib, of which we did not consider TAK 733 and CH5126766. For Trametinib and PD0325901 the authors did not observe any pronounced difference between RAS mutant and BRAF mutant cell lines. For cobimetinib and selumentinib, they appear to observe less inhibition in BRAF mutant compared to RAS mutant cell lines, but it is unclear whether these differences are statistically significant. However, the authors of that study employed concentrations (20uM and 200uM) that are much higher than those at which we observed differences between BRAF mutant and RAS WT cells ($10^{-3}$ to 1uM).

The papers cited by the reviewer involve *KRAS mutant lines* rather than RAS WT lines (as in our paper) and the MAPK-activating signal arises in the from KRAS rather than EGF-EGFR in our lines. These are very different forms of pathway activation (EGF-EGFR is inherently transient). Nonetheless, if we compare the MEK inhibitors that are studied in the both our paper and the papers cited by the reviewer, the findings are broadly consistent and our dose response measurements actually show that seemingly conflicting findings in previous studies (e.g., for cobimetinib) were the result of only considering drug response at one or two concentrations.

The reviewer has nonetheless raised an important point that we now address in our discussion – namely the differences that are likely to arise when MAPK pathways are activated by different oncogenic or physiological stimuli. We agree that this will be an interesting area to explore in the future.

**Reviewer 1 (Major Point 7):**
7. 'The resulting isobolograms had a convex shape (Figure 6E) with minimal drug interaction by Bliss (Figure 6F) or HSA criteria (Figure 6G). This differs from what was observed with pulsatile RTK activation (Figure 5C, D bottom panels) and suggests that drug interactions in the case of pulsatile signaling were only possible due to time scale separation between drug adaption and direct drug action.'
- In Figs. 5 - 7 isobolograms are given on log-log scales. On log-log scale, a straight line that represents drug additivity will be convex, making it impossible to distinguish drug antagonism from additivity. The Bliss criterion is a weak criteria used only for a preliminary screening of thousands of drug interactions. For instance, the Bliss criterion might show that a drug is synergistic or antagonistic with itself, although two doses of the same drug would merely be additive (see, e.g., doi: 10.1038/nrmicro2133). All isobolograms must be shown on linear scales where they would clearly show the areas of drug additivity, synergy and antagonism (PMID: 7568331).

**Response:**
We thank the reviewer for this comment. It was not our intention to use isobolograms for analysis of drug sensitivity, but rather to visualize the qualitative shape of the dose response surface. The comment about convexity of the surface and it is juxtaposition with analysis of drug interactions was erroneously introduced during editing of the manuscript and we have now removed it.

We are familiar with all of the points raised by reviewer about isobolograms but wish to point out that plots on a linear scale suffer from the problem that they can cover only a narrow range of drug doses. The reviewer will also appreciate that we evaluated drug interaction numerically, not visually, so the way the data are plotted does not affect our conclusions.

It is well known that the Bliss criteria does not satisfy the sham principle, but we do not agree with the conclusion that this makes it a “weak criteria”, and this conclusion is also not supported by the referenced literature. The Bliss model is generally thought to be directly applicable to understanding cell killing by cancer cells, and is widely referenced in the literature with respect to concepts such as log-killing and chemotherapy.

Moreover, Greco *et al.* (cited above, PMID: 7568331) write “Loewe additivity and Bliss independence are included as reference models, because each has some logical basis, and especially because each has its own coterie of staunch advocates who have successfully defended their preferred model against repeated vicious attacks”. We did not want to favor one null model over another, but, as we explain in manuscript (l. 631-633), we found ourselves unable to analyze drug interactions according to Loewe Additivity as we observed disconnected isoboles for some of the experiments (**Figure 5B**). If the reviewer has any ideas how to quantify drug interactions according to Loewe in such settings, we are happy to include the respective analysis.

We now provide isobolograms on a linear scale in the supplementary material.

**Reviewer 1 (Major Point 8):**
8. Lines 741-743. 'To investigate whether MARM2.0 could predict the responses of BRAFV600E colorectal cancers to RAFi, we collected data from HT29 cells, which carry a BRAFV600E mutation and have high EGFR expression (similar to A375 EGFR-CRISPRa cells).
- Fig. 9 only shows simulations. Experimental data need to be obtained and imposed on simulated responses, as in other figures.

**Response:**
We are not sure how the reviewer arrived at the conclusion that Figure 9 does not include experimental data. Experimental validation for all predictions is shown in the right panels in of Figure 9C and Figure 9D. We hypothesized that it was possibly unclear what left/right in the text was referring to and have updated the respective text make it clear that this was referring to figure panels. We have also updated the caption of Figure 9.

**Reviewer 1 (Major Point 9):**
9. HT29 cells (unlike A375 cells) have a heterozygous BRAFV600E mutation, bearing a WT BRAF allele. Does A375 model describes cells with homo- and heterozygous BRAF mutations?

**Response:**
We thank the reviewer for this comment. The model currently does not account for heterozygous BRAFV600E mutations, but, as described in the response to Major Point 1B, EGFR over-expression is a sufficient explanation for the data we describe. While it would be easy to extend the model we have not collected HT29 training data (precisely because we wanted to highlight the key role of EGFR over-expression). We have updated the results and the discussion to reflect these conclusions and limitations.

## Minor points

**Reviewer 1 (Minor Point 1):**

1. Figure legends must provide more details. Currently these legends are unhelpful. For instance, the legends to Fig. S2B,F do not tell if drug-naive cells were also kept for 24 hr after EGF addition. Additional clarity is needed for legends to Fig. S3 and others. Figures with isobolograms lack doses on the axes, and must be generated on linear scale.

**Response:**
We thank the reviewer for these comments and have carefully revised all figure captions to provide more details about model simulations and experimental details. Figures S2B,F do not include any EGF response data, but drug-naïve cells were pretreated with DMSO for 24h.

**Reviewer 1 (Minor Point 2):**
2. Lines 307-308 'MARM2.0 nonetheless has over 15,000 biochemical reactions' and Lines 356-357 ... 'generated >2,200 molecular species and >30,000 biochemical reactions'
- these two statements must be reconciled, because the numbers are inconsistent.

**Response:**
We thank the author for identifying this inconsistency. The number 15.000 was referring to an earlier version of the model and we have updated both numbers to reflect the increased size of the updated model.

**Reviewer 1 (Minor Point 3):**
3. Line 303: 'panRAFi'
- The term 'pan' refers to the ability of these RAFi bind both BRAFV600E and CRAF. However, Vemurafenib also binds both RAF isoforms with similar affinities (https://www.selleckchem.com/products/PLX-4032.html). Most importantly, these RAFi are Type II RAFi, whereas Vem and Dabrafenib are type I1/2 RAFi, which should be clarified in this piece.

**Response:**
We thank the author pointing this out. The concept and modes of action of type II (and the name panRAFi) as well as type I1/2 RAFi are explained in Box 2, which is unfortunately only referenced in a later section of the text (see line 692). We have included a reference to this Box in the paragraph the reviewer referred to as a more detailed explanation of panRAFis would have interrupted the flow of the manuscript.

**Reviewer 1 (Minor Point 4):**
4. Why do you assume that the decomposition into two different signaling fluxes can be termed causal?

**Response:**
We use the term causal based on the terminology of "causal tracing" that was first described in the context of agent-based models and which the labelling strategy that we developed is based on. The method is `causal` because it accounts for the causality encoded in the reaction patterns of the rules that were used to define the start of the respective reaction channels (please see response to Major Point 1C).

**Reviewer 1 (Minor Point 5):**
5. Fig.2B must show RAS and indicate whether RAS facilitates the formation of BRAFV600E homo- and hetero-dimers.

**Response:**
The goal of Figure 2B is to show which channels are active under the conditions shown in Figure 2A. This is illustrated based on the perturbations and mutations that activate respective channels. We wonder whether adding details about RAF homodimerization would be helpful in this setting – this detail is found elsewhere in the

paper. In this figure we are trying to make our findings more accessible to individuals in the cancer field more familiar with concepts such as “rewiring.” We have updated the figure caption to better explain what we are aiming to illustrate in this panel.

**Reviewer 1 (Minor Point 6):**
6. Lines 531- 532 'The ratio of input to output signals in a network (the gain) is a fundamental property of a signal transduction system that can be used quantify rewiring.'
- Gain is the ratio of output to input signals.

**Response:**
We thank the reviewer for pointing us to this slip of the pen. We have corrected the description in the text and verified that gain was indeed correctly computed in our analysis.

**Reviewer 1 (Minor Point 7):**
7. A cartoon in Fig. 4A is misleading. RAS signals to MEK via RAF isoforms, monomers and dimers.

**Response:**
The goal of Figure 4A is to introduce a simplified model topology that is then used in the following panels of the same figure. As a simplification, the cartoon cannot show every detail of the underlying implementation. The distinction into RAF monomers and dimers is shown in Figure 4F and discussed in the respective text. We have updated the figure caption to better explain what we are aiming to illustrate in this panel.

**Reviewer 1 (Minor Point 8):**
8. The Fig. 4A cartoon contradicts to the following sentence: 'We found that drug adaptation to RAFi and MEKi had a similar impact on the first step (R1) of both reaction channels (Figure 4C, top panels).
- Both channels, BRAF600E and CRAF monomers and all RAF dimers, signal to MEK, thus MEK must be explicitly shown for both channels'. In fact, the step R1 is shown only for the EGFR/RAS channel.

**Response:**
We thank the reviewer for pointing out this mistake. Instead of reaction channels, this sentence should have “for both drugs” and we have updated the text accordingly. MEK is explicitly shown for both channels (see Figure 4B). There is no step B1 since BRAFV600E need not have any upstream activators.

**Reviewer 1 (Minor Point 9):**
9. Line 572. 'Thus, even at 10μM, the highest vemurafenib concentration tested, and a value well above the clinically useful range, ...'
- Clinically, vemurafenib concentrations as high as 100μM in blood were reported (doi:10.1093/annonc/mdv189).

**Response:**
The reviewer is correct that concentrations above 10μM have been measured in patients. This is well above the *intended* concentration, but given the ambiguity we have removed this statement from the text. The key issue, of course, is the concentration in tumor cells, and this remains largely unknown (as an aside, we want to mention that the article the reviewer is referring to reports plasma concentrations in μg/ml, not μM).

**Reviewer 1 (Minor Point 10):**
10. 'Moreover, estimated ranges for $\Delta\Delta G$ were similar for the four other type I1/2 RAFi drugs we tested (Figure S1, S4).'

- What are these I1/2 RAFi drugs? In the experiments shown only Vemurafenib was tested.

**Response:**
We thank the reviewer for pointing to this careless mistake, the model of course only contains two other type I1/2 RAFi (Dabrafenib and PLX8394). We have updated the text accordingly and now explicitly mention the respective RAFis.

**Reviewer 1 (Minor Point 11):**
11. 'In the absence of exogenous growth factors (Figure 5A(i)), we predicted a monotonic decrease in pERK levels'
- Was serum present in the media as it is normally present?

**Response:**
We mention serum concentration in the Methods section "Drugs and growth factors". A375 cells were cultured under low serum concentrations (5%) and HT29 cells were cultured under normal serum concentrations (10%). Thus, the reviewer is correct and growth factors may be presented even when no exogenous EGF is added, however it remains true that cells remain responsive to EGF under these conditions. There reviewer will appreciate that there is a lot controversy about the use of serum starvation for experiments such as ours, and we therefore avoid it. However, it is likely that cells consume much of the EGF naturally present in the medium prior to the start of the experiments we describe.

**Reviewer 1 (Minor Point 12):**
12. Line 627. '...recapitulating the non-monotonic response to MEKi in Figure 2A, in which pERK levels first rose and then fell with increasing drug concentration.'
- Fig. 2A shows the kinetics/time-course of the MEKi response. How can the steady-state isobolograms relate to details of the kinetics?

**Response:**
Response: The isobolograms shown in Figure 5B are not at steady-state, but quantify combination response at the peak of the pERK pulse at 5 minutes after stimulation with EGF. We have updated the respective text accordingly.

**Reviewer 1 (Minor Point 13):**
13. Lines 200-202. 'Thus, the structural differences between monomers and dimers (rather than differences in the ATP binding pocket) are the basis of the selectivity of clinically approved RAF inhibitors for cells transformed by BRAF mutant kinases.'
- There is other critical structural difference between RAF inhibitors. DFG-IN, αC-IN RAF inhibitors promote RAF dimerization much stronger than DFG-OUT, αC-IN RAF inhibitors (doi: 10.1016/j.ccell.2016.06.024), and this might be a reason why DFG-IN, αC-IN RAF inhibitors are no longer being developed.

**Response:**
We discuss the difference in strength of dimer induction between type I½ DFG-IN, αC-OUT and type II DFG-OUT, αC-IN inhibitors in the text of the same box. DFG-IN, αC-IN RAF inhibitors are not mentioned in the manuscript and given the complexity of the manuscript and their clinical irrelevance we would rather avoid doing so.

**Reviewer 1 (Minor Point 14):**
14. The exact formulas for different synergy scores need to be presented in Methods, because different authors use slightly different equations for the same Bliss criteria, etc.

**Response:**
This is a good point and we agree. We have added a section to the Methods that provides formulas for drug interaction scores according to Bliss Independence and Highest Single Agent null models.

**Reviewer 1 (Minor Point 15):**
15. Model assumptions must be formulated in the main text and Methods, and not only in supplemental Python files.

**Response:**
Please see our response above; we expect that including a description of the model and its assumptions in the main manuscript that is as detailed as what we provide in the supplementary materials would easily double or triple the length of the manuscript. If the editor agrees that this would improve the manuscript and is something that MSB allows, we are happy to revise the manuscript accordingly.

# Reviewer #2

**Reviewer 2:**
In the present manuscript, Fröhlich and coauthors describe the development, parameterization and application of a detailed MAPK signal transduction model. The model is an extension of a previously published model that contains a bit more details (mainly compartmentalization, receptor endocytosis), and is trained on some additional data. Overall, it is conceptually similar to the previous model. As they write: "(the previous paper did) not involve any model analysis. Such analysis is the focus of the current paper and its updated model". Large parts of the results describe details of the model construction (which I think is carefully done) and optimization of the model calibration procedure that helps to reduce runtime for calibration. Though this is very technically, I think this is an important part and really the strength of the paper (though maybe more for a technical journal). Then the authors introduce what they call "causal decomposition". By introducing additional variables ("labels"), they can then trace back how much of the pERK signal derives from WT RAF / RAS and mutant RAF. I think this is the most important conceptual advance of the manuscript. By doing so, they provide a framework to analyze the dynamics of the very complex and large model in rather simple terms, i.e. they provide a narrative of how blocking mutant RAF enables RAS-mediated activation of MAPK signaling. However, mechanistically this has been known for a while and is also discussed in their previous paper that included the MAPK model. Thus, while it is a technical advance, its biological novelty is not really clear, and the quantitative validation of the model (which would be the advance over previous studies) is not really done.

**Response:** We thank the reviewer for the encouraging words. However, we are not sure why the reviewer concludes that we do not quantitatively validate the MARM2.0 model with extensive data unique to this manuscript (and not included in our more qualitative 2021 study Gerosa et al). We quantitatively validate model predictions on data for (i) type II RAF inhibitors (**Figure 7A, B**), (ii) EGFR amplified A375 cells (**Figures 6D**) (iii) NRAS mutated A375 cells (**Figures 8B,C, 9D**) and (iv) HT29 cells (**Figure 9D**). None of these datasets that were considered in the previous manuscript and we discuss these validations in detail, which, in our view, manifests a rather extensive validation of the model.

Since, the reaction channels are the result a conceptual frame and do not have any direct physical manifestation, these datasets of course do not provide a direct validation of reaction channel idea. However, we carefully selected experimental conditions under which the different reaction channels can be independently activated and deactivated and thus provide means to indirectly validate of the reaction channel concept.

**Reviewer 2:**
They then use the model to evaluate dose-response curves of multiple inhibitors and cell lines. While this is generally nicely done and models are mostly in good agreement with the response curves, it merely shows the existence of a feedback in the system, but a real and direct quantitation of the contributions is missing.

**Response:** We agree with the reviewer that a direct quantitation of contributions of these forms of feedback control would be interesting. However, discriminating among overlapping feedback mechanisms is very difficult (see above and **Figure 4C**). From the perspective of genetics, the problem is that each circuit involves multiple redundant phosphorylation sites and multiple phosphatases – some constitutively expressed and some inducible.

As we describe in Section “Slow Transcriptional Feedbacks Imprint Drug-Adapted State and Unravel Cyclic Causal Dependencies”, the quantification of “contributions” of feedback mechanisms from the perspective of a computational model is in general non-trivial due to the cyclic structure they impose on the underlying causal model. Rigorous quantification of causal contributions can only be achieved using counterfactual analysis, which is not directly applicable to ODE models (without other adjustments for model complexity). Counterfactuals operate at the level of individual events (see doi.org/10.24963/ijcai.2018/260). The ODE formulation marginalizes over individual events, rendering counterfactuals, poorly defined. Nevertheless, it is, in principle, possible to define similar operations on ODEs by altering model parameters or structure (see, e.g., variable interventions in doi.org/10.48550/arXiv.2106.12430). However, in this study, we impose a steady-state constraint to account for drug adaptation, which in the presence of cycles in the causal structure, makes it impossible to define interventions that have the surgical precision of counterfactuals. For example, to quantify the contribution of the feedback through DUSP, we would have to fix DUSP protein expression levels to some reference value (e.g., baseline level without drugs/growth factors) independent of pERK levels. However, this would always also alter the pERK levels, thus affecting the strength of other feedback mechanisms. This makes it *de facto* impossible to

quantify the contribution of the DUSP feedback without extensive training data from mutant cell lines that are not currently available.

To improve the logical flow of the manuscript and address the reviewer's concerns at more general level, we have revised the section "Slow Transcriptional Feedbacks Imprint Drug-Adapted State and Unravel Cyclic Causal Dependencies" to better explain how the time-scale separation and causal analysis is conceptually required to decompose the contributions of individual feedback mechanisms.

## Major Points

**Reviewer 2 (Major Point 1):**
My main concern is that the presented analysis suggests a very quantitative framework to dissect contributions of the channels, and also the detailed models of signaling upstream of RAF suggests that the mechanisms are very precisely covered. Yet, the models do not contain some basic important mechanisms that may also contribute (or maybe even contribute much more), which is e.g. a very strong MAPK feedback to RAF, and obviously lack data to really quantitatively dissect contribution of different feedbacks. Thus, it remains unclear (apart from the technical advances) why and how such a detailed model contributes to our understanding or MAPK signaling or resistance.

Thus, while I applaud the authors for their technical rigor and the enormous amount of data and work that went into model calibration, I don't really see the major advance in our understanding that arises from this model.

**Response:**
We thank the reviewer for raising these points.

With respect to RAF feedback, we agree with the reviewer have included it in a revised version of the model. Repeating model analysis did not result in any notable changes in simulations and predictions and, thus, did not affect conclusions of the manuscript (as described above)

We are not quite sure what a novel biological insight might mean in this context other than a better understanding of the interplay between individual protein activities under different conditions of pathway activation and inhibition. Decades of experimental work on the MAPK pathway have yielded a good understanding of key components and their individual activities. The purpose of models such as ours to demonstrate that a quantitative and mechanistically sufficient understanding of a process has been achieved. Sufficiency is important in this regard because mammalian signal transduction suffers from a surfeit - not a shortage - of possible mechanisms. Dynamical modeling helps to reveal which among a sea of proposed interactions is quantitatively significant.

More specifically, the response to reviewer 1 (and our revised paper) includes detailed explanations of (i) the choice reaction channels (see response to **Reviewer 1 Major Point 1A,C**), (ii) the net actions of MEK inhibitors (see response **Reviewer 1 Major Point 3,6**) (iii) the approach to quantify contributions of feedback mechanisms (see response above) are all new conceptual insights into MAPK signaling as is the (iv) observation and explanation of drug interactions that specific to pulsatile signaling. We also demonstrate a dependence on time-scale separation by demonstrating that drug interactions disappear in sustained signaling settings (**Figure 6D-G**). Moreover, the fact that Reviewer 1 found some of these ideas controversial is one sign that they are new!

An additional aspect of this work is that it required the development of new model analysis methods to uncover the properties of reaction channels. Thus, the "technical rigor is tightly coupled to conceptual innovations.

## Minor Points

**Reviewer 2 (Minor Point 1):**
Minor: In part, the manuscript uses very bold language that the authors should avoid given the level of evidence. E.g.:

"to model the precise meachanism" (abstract). Isn't it rather one plausible mechanism? "powerful constraints" - what is this exactly? What does powerful mean here?

**Response:**
We agree with the reviewer that the language in the referenced text was perhaps to strong (we were aiming for clarity not overstatement) and we have revised the manuscript. We now no longer claiming the mechanisms are “precise” and describe the thermodynamic constraints as “rigorous” rather than “powerful”.

# Reviewer #3:

**Reviewer 3:**
Frohlich et al. present an interesting manuscript that details an extension of the original MARM model previously published by the authors. MARM 2.0 is a thermodynamic model that leverages differences in free energies to implement a rule-based system that captures many more complex interactions than would be practical with simple ODE modeling. This new model allows a more detailed dissection of signal rewiring in BRAF V600E melanoma in the presence of single-agent or combination therapies, and leads to several non-intuitive insights that were verified experimentally. Among other highlights, the authors use their model to trace signal flow through two distinct but parallel channels of Ras-to-Erk signaling, and measure how the flux ratio changes as a function of drug dose. The model also allowed the calculation of the gain from one node to the next at every step in the pathway, and found instances of increased gain at nodes that correlate with network nodes observed to be activated during resistance. The collective transfer functions between nodes, together, could predict the signaling response to combinations of BRAF and MEK inhibitors. Finally, the model also predicted responses to drug combinations in the presence of EGFR overexpression and additional resistance-associated mutations.
Although I am not well-positioned to evaluate the details of the modeling or its implementation, the model addresses an interesting and important biological question of signal rewiring in BRAF V600E melanoma, and allows a detailed and testable breakdown of how signal flux is altered during drug treatment. Moreover the paper is thorough and is written with a high degree of clarity, which is challenging for such a complex topic and model. This work will allow better prediction of how targeted therapies rewire signaling in drug adapted cancer cells, which will expedite the process of combinatorial therapies to combat cancer resistance. In addition the model seems to represent several methodological advances that should be broadly applicable to various modeling scenarios. Thus I recommend publication with only a few minor comments below:

**Response:**
We thank the reviewer for this positive summary of our work and the positive evaluation.

## Minor Points

**Reviewer 3 (Minor Point 1):**
-Although there are callouts for boxes 1,2,and 3, I didn't see figures for Boxes 2 and 3 in the manuscript

**Response:**
The text of Boxes 1, 2 and 3 is provided in the Section “RESULTS - TEXT BOXES 1 TO 3”. To give a better visual indication, have now surrounded the respective text with boxes similar to what these boxes will look like in the final manuscript at MSB.

**Reviewer 3 (Minor Point 2):**
-All experimental validations are performed with 100 ng/mL EGF, which is a very strong, superphysiological signal. Could the authors comment on how the model would perform at different stimulus concentrations?

**Response:**
The experiments in this paper we performed on cells grown in the continuous presence of 5-10% serum, without starvation. Under these circumstances, 100 ng/mL EGF elicits a response similar to serum-starved cells treated with EGF concentrations 100-1000 lower. None of the conditions used in cancer cell culture are optimally representative of cells growing in actual tumors, but we decided that continuous culture conditions were more realistic than serum starvation, particularly when 24 hr of drug pre-treatment was included in many experiments.

One other important point is that we use high-dose EGF to force all cells to be synchronous in their behaviors. In this case a population-average ODE-based model is a reasonable representation of a single cell. Single cell analysis of data we have previously collected (doi:10.1016/j.cels.2020.10.002 Figure 3I) show that single cells respond to low concentrations of EGF (down to ~0.01 ng/mL) in a non-uniform manner. Population average transcript profiling and proteomic data of the type we use to calibrate our model are not good representations of cells under these conditions. Moreover, under normal conditions of drug adaptation (i.e. without added EGF) drug-

adapted MAPK in A375 cells pulse very irregularly, often only once every few days. It would be very nice to model this single-cell behavior directly but we do not yet know how – in part because we are not yet able to measure or model the growth factor gradients that appear to drive MAPK pulsing.

We believe that pulsing derives from hypersensitivity to EGFR expression levels and sporadic growth factor release. High EGFR expression levels may lead to a preforming of EGFR dimers that have higher affinity to EGF and may thus sense EGF at lower concentrations. The energetic formulation description of these allosteric interactions is already included in the model. However, depletion of EGF molecules will also influence duration and shape of the pERK pulse at lower EGF concentrations, which is currently not accounted for in the model.

We have revised the text to make these assumptions and limitations in our approach more clear.

**Reviewer 3 (Minor Point 3):**
-in some cases, figures are called out of order (e.g. Figure 1B is called out after 1C and 1D)

**Response:**
We thank the reviewer for spotting this mistake and have added a reference to Figure 1B before 1C and 1D and have changed the order of Figure S1 and S2.

**Reviewer 3 (Minor Point 4):**
-line 428: the term "wall-time" wasn't familiar to me

**Response:**
Wall-time, is a common term in computational studies that refers to the time in the real world, in contrast to CPU time, which also accounts for parallelization on multiple cores. We updated the text and now refer to 'real-time' to make the text more accessible for a biological audience.

**Reviewer 3 (Minor Point 5):**
-in figure 3B, it was difficult to see the differences between brown and purple shading of the arrows.

**Response:**
We have doubled the opacity of the shading and it should now be easier to tell the difference between the colors.

**Reviewer 3 (Minor Point 6):**
-starting on line 514, the switching of cyclic to acyclic networks is interesting. It would help if the authors would clarify why this is important. Is this to more easily be able to understand the causality in the network? Perhaps a sentence or two would help motivate the approach.

**Response:**
Yes, the reviewer suspicion is correct. Establishing acyclicity of the causal diagram is one of the conceptual prerequisite for the subsequent analysis presented in Figure 4. We have revised the respective section accordingly (see also response to **Reviewer 2**)

**Reviewer 3 (Minor Point 7):**
-Figure 1B - the title is "oligomerization", but it seems this is better described as "binding". Oligomerization to me implies a higher-order interaction.

**Response:**
The reviewer is correct, while the model of course describes oligomerization including higher-order interactions, this is not shown in the figure panel and we have updated the title accordingly.

**Reviewer 3 (Minor Point 8):**
-Fig 2F: legend says that data are represented as point ranges, which I understand to mean that the bars span the min and max measurements. But what are the points? And sometimes there are 2 points and sometimes one point. Same in Fig 6B. In general, the authors could include more details on data representation in the legends.

**Response:**
The bars/lines show **±**1 standard deviation and the points show averages over multiple technical replicates. Two points indicate data from separate experiments, i.e. biological replicates. We have revised all figure captions to provide more details.

**Reviewer 3 (Minor Point 9):**
-Figure 4C: it took me a while to understand what the 3 rows of plots represented. Would be clearer if they were labelled "R1/B1", "R2/B2" or similar.

**Response:**
We agree with the reviewer and have added the labels as suggested and updated the legend to provide more details about what is shown.

**Reviewer 3 (Minor Point 10):**
Figure 4D: the diagram is a nice way to summarize the data, but I don't think I understood it. Why is pEGFR becoming more transparent with added vemurafenib/MEKi? Why is BRAF (blue circle) not becoming more transparent with increasing vemurafenib?

**Response:**
It sounds like the reviewer correctly understood the visualization approach, but did not connect this to the transcriptional feedback from pERK on EGFR expression levels. This is perhaps understandable given the overall complexity of the manuscript. Lower EGFR levels with increasing drug concentrations result in a decrease in pEGFR levels. We have added an explanation to the text that describes the figure and apologize for the previous lack of clarity,

**Reviewer 3 (Minor Point 11):**
Figure 5G: it would help clarify the comparison between 5A and 5G is the "L" shape in 5G were outlined, as in 5A.

**Response:**
We thank the reviewer for this comment and fully agree with it. We have added the same L-shaped region to Figure 5G.

**Reviewer 3 (Minor Point 12):**
Figure 8D: It took me some time to understand here that NRAS is dox-inducible. This could be clarified in the figure or in the caption

**Response:**
We have updated the figure caption to clearly mention that NRAS is dox-inducible.

**Reviewer 3 (Minor Point 13):**
Figure 8D, top right. In many figures, it’s not clear what the heatmap is showing. I think here it is pErk, but this should be made clear in every figure and caption.

**Response:**
The axis label for the colorbar in Figures 8D and S7 were indeed missing, which we have fixed. Moreover, we have clarified in all Figure captions that  the heatmaps depict pERK.

**Reviewer 3 (Minor Point 14):**
-Figure S7 is called out in at least 2 different places (line 701 and line 731), but neither description fits what is in Figure S7, as far as I can tell (also the current S7 is labelled as S5)

**Response:**
We thank the reviewer for noticing this oversight and apologize for the error. The first reference was referring to a nonexistent figure and we have removed the respective text. However, the reference in line 731 was indeed supposed to reference Figure S7 in the submission (which is indeed mislabeled as S5, an error we have corrected), This missing figure showed model simulations corresponding to experimental data shown in Figure 8D.

# Referee Cross-commenting

**Reviewer 3:**
Reviewers 2 and 3 bring up many strong points. In my view, the model appears to be a big technical achievement, and would warrant publication largely on these grounds. Although there is a lack of validation of a deep conceptual advance, as Rev. 2 points out, there are interesting predictions made that can shape the way we think about signal rewiring in this cancer system. Rev. 1 raises many important technical discrepancies or omissions/assumptions made by the model. Of course all models are by their nature simplifications, even ones as complex as the ones presented. In my view the authors could be reasonably expected to respond to these critiques and either better justify their assumptions, or alter their model to accommodate. I also agree that, given the complexity, a general increase in details is called for.

**Response:**
We thank the reviewer for his comments regarding the simplifying nature of all models. We hope that our response to reviewer 2 shows that we do provide new conceptual insights, which we validate with experimental data. Similarly, we hope that our response to reviewer 1 shows that a large fraction of the comments are the results of disagreements about terminology rather than factual omissions in the model.

**Reviewer 2:**
I agree that the manuscript would be a big technical achievement - however, the previous version of the model is already published in the cell systems paper which is hardly different from the one reported here. Actually, the current model lacks a very important component (ERK to RAF feedback, see comment 2 of reviewer 1) that the previous model had included, so I don't see it as a major way forward but rather sideward.

**Response:**
We do not think that the model itself constitutes a technical achievement. The technical advances we make are improvements to model calibration and the introduction of novel approaches for model analysis, none of which have been described in the cell systems paper.

We also want to note that the ERK to RAF feedback was not included in the previous model published in cell systems. The “previous studies” in the quote in comment 2 by reviewer 1 refers to literature in the field in general and not our own work specifically.

**Reviewer 1:**
A major shortcoming of the current submission is a misleading and too simplistic division of RTK to ERK signaling into a BRAFV600E monomer signaling channel and a RAS signaling channel. Neither of these two channels account for signaling by WT BRAF (present in many BRAF mutant cells) monomers and CRAF monomers. It is also well known that BRAFV600E signaling is dramatically enhanced with increasing RAS-GTP levels because of the corresponding increase in BRAFV600E dimerization homo and hetero-dimers. Another critical omission is the lack of ERK inhibitory feedback to RAF isoforms, including BRAFV600E, which is known to play a crucial role in resistance to RAF inhibitors. If the authors fix their model's flaws and correct the ambiguous and improper terminology of two reaction channels, the manuscript can be published in MSB.

**Response:**
We have revised the description of the two reaction channels based on the reviewer’s feedback and hope that it is no longer ambiguous or improper. Moreover, we acknowledge the importance of the RAF feedback and have included it in a revised version of the model.

## Appeal- Editorial Decision 9th Jun 2022

Thank you for your message asking us to reconsider our decision regarding your manuscript MSB-2022-10988. I have carefully read the referee reports once again and have also discussed your point-by-point response with my colleagues. Based on the response you provided, we think that the proposed revisions sound reasonable. As such, we would welcome the submission of a revised version for further consideration.

As you may already know, our editorial policy allows in principle a single round of major revision. As acceptance or rejection of the manuscript will depend on another round of review, your responses and revisions should be as complete as possible.

On a more editorial level, we would ask you to address the following issues:

Thank you for sending us your revised manuscript. We have now heard back from the three reviewers who agreed to evaluate your study. From the comments below, you will see that Reviewer #3 was satisfied with the revisions. The other two reviewers still raised a number of issues, while Reviewer #2 is overall more supportive. Given the balance of these evaluations, we feel that we can consider a revision of your manuscript if you can address the following issues that have been raised.

- Both Reviewers #1 and #2 raised concerns about the model and were not convinced that the model generates a precise and quantitative description of MAPK signaling. Therefore, we think it is essential to discuss the limitation of the model (in light of Reviewer #1's comments) and tone down the claim about the model being quantitative.

- Considering the technical advance of the study, which Reviewer #2 thought was the strength and value of the manuscript, we would ask you to revise the manuscript to focus more on the technical aspect.

--------------------------------------------------------------------------------

Reviewer #1:

Fabian Froehlich and colleagues put considerable effort into the manuscript revision. They clarified key definitions and incorporated the missing inhibitory RAF phosphorylation by ERK in the model. Although I think that the paper can be eventually published, my main previous concerns were not addressed. The authors' use of 'creative' language, such as 'causal decomposition', and a concept of two reaction channels creates the illusion of understanding but does little to deepen biological insights. This language also accomplishes little to convince biologists to see how dynamic model predictions can help them in their efforts to understand why the ERK signaling in cells is genetically resistant to RAF/MEK inhibitors or rapidly acquires resistance.

Major point 1.
In the revised manuscript, the authors properly clarified their definition of "two reaction channels". However, this concept in my opinion does not really help us understanding the mechanism of the ERK pathway signaling and the development of drug resistance.
Lines 369-383 of the main text and the author's code define a 'RAS reaction channel' that involves RAF dimers bound to RAS. A 'BRAFV600E reaction channel' involves BRAFV600E monomers and dimers that are not bound to RAS or dimers with only one molecule RAF bound to RAS. However, even if the RAF dimer is not bound to RAS, it is still resistant to αC-OUT, DFG-IN RAF inhibitors (RAFi). Thus, the BRAFV600E reaction channel is also partially resistant to αC-OUT, DFG-IN RAFi. Moreover, the part of the BRAFV600E channel that consists of free dimers unbound to RAS changes depending on the RAFi. Almost all RAFi will increase the amount of such dimers. In the case of p61 BRAFV600E splice variants such dimers will be dominant, making the BRAFV600E reaction channel completely resistant to αC-OUT, DFG-IN RAF inhibitors.
Thus, the concept of reaction channels fails to clearly distinguish the molecular complexes resistant to αC-OUT, DFG-IN RAF inhibitors and the molecular complexes sensitive to αC-OUT, DFG-IN RAF inhibitors. Moreover, this formal definition of reaction channels does not cover the known resistance mechanisms and also differs from the accepted concept of RAF monomers and dimers. Therefore, the usefulness of this concept is questionable.

Major point 2.
It was previously shown that both RAF monomers and dimers have non-zero kinase activity [doi:10.1128/MCB.26.6.2262-2272.2006]. It was also shown that BRAF-CRAF dimers have higher kinase activity than BRAF-BRAF and CRAF-CRAF dimers [doi:10.1128/MCB.26.6.2262-2272.2006]. Neither of these facts is incorporated into the present model. RAF monomers, except BRAFV600E, have no kinase activity in the model. In the model, the kinase activity of BRAFV600E monomers and all RAF dimers depends on the so-called channel (that is on whether the RAF dimer is bound to RAS-GTP) whereas it also critically depends on the composition of a RAF dimer, as mentioned above.

Major point 3.
The authors do not consider dimer asymmetry in the model, having only one factor g, instead of introducing three factors, g_i, for an asymmetric dimer - inhibitor interactions This shortcoming can lead to incorrect descriptions of RAFi binding and RAF dimerization reactions in the model.
For example, in the Figure EV5D (in the updated manuscript the authors refer to Figure EV5E in line 645, but I think this is EV5D), the authors attempted to predict the effect of combining three drugs: LY3009120, Cobimetinib, and Vemurafenib. When incorporating structurally different RAF inhibitors to the model (in this case type II and type I1/2), we need at least four independent thermodynamic factors for each RAFi. In the model, the authors implement the allosteric effect caused by RAFi as follows (cell 15 in Model Documentation.ipynb, RAF inhibition section):

```
## Single RAFi bound Raf dimers
EnergyPattern('ep_BRAF_BRAF_mod_RAFi_single',
BRAF(raf=1) % BRAF(raf=1, rafi=2) % RAFi(raf=2),
ep_RAF_RAF_mod_RAFi_single_ddG)
EnergyPattern('ep_BRAF_CRAF_mod_RAFi_single',
BRAF(raf=1) % CRAF(raf=1, rafi=2) % RAFi(raf=2),
ep_RAF_RAF_mod_RAFi_single_ddG)
EnergyPattern('ep_CRAF_BRAF_mod_RAFi_single',
CRAF(raf=1) % BRAF(raf=1, rafi=2) % RAFi(raf=2),
ep_RAF_RAF_mod_RAFi_single_ddG)
EnergyPattern('ep_CRAF_CRAF_mod_RAFi_single',
CRAF(raf=1) % CRAF(raf=1, rafi=2) % RAFi(raf=2),
ep_RAF_RAF_mod_RAFi_single_ddG)

## Double RAFi bound Raf dimers
EnergyPattern('ep_BRAF_BRAF_mod_RAFi_double',
RAFi(raf=2) % BRAF(raf=1, rafi=2) % BRAF(raf=1, rafi=3) % RAFi(raf=3),
ep_RAF_RAF_mod_RAFi_double_Gf)
EnergyPattern('ep_BRAF_CRAF_mod_RAFi_double',
RAFi(raf=2) % BRAF(raf=1, rafi=2) % CRAF(raf=1, rafi=3) % RAFi(raf=3),
ep_RAF_RAF_mod_RAFi_double_Gf)
EnergyPattern('ep_CRAF_CRAF_mod_RAFi_double',


RAFi(raf=2) % CRAF(raf=1, rafi=2) % CRAF(raf=1, rafi=3) % RAFi(raf=3),
ep_RAF_RAF_mod_RAFi_double_Gf);
```

In the supplemental Jupyter Notebook the authors wrote:
> In this implementation, C-Raf and B-Raf both correspond to kinase monomers R of the Kholodenko Core Model (KCM). K1 of the KCM is equivalent to the parameter bind_RAF_RAF_dG, which was introduced in the previous Section. K2 in the KCM is equivalent to bind_RAFi_RAF_dG, f to ep_RAF_RAF_mod_RAFi_single_ddG and g to ep_RAF_RAF_mod_RAFi_double_ddG.

Here the number of independent thermodynamic factors is two: ep_RAF_RAF_mod_RAFi_single_ddG and ep_RAF_RAF_mod_RAFi_double_ddG.

Therefore, the model presented in this study cannot quantitatively describe the effect of combination treatment with two structurally different RAFi and MEKi. This effect is not negligible because a combination of type II RAFi and a type I1/2 RAFi is highly synergistic (doi: 10.1016/j.cels.2018.06.002). Even if the association/dissociation rates of the RAFi-RAF complex is fitted to be RAFi-specific via parameter estimation, the model does not describe the change in the affinity of second RAFi binding caused by the first structurally-different RAFi binding. This can lead to wrong conclusions about inhibitor synergy/additivity/antagonism. Thus, the text and figure related to triple combination predictions must be deleted, or the model should be amended.

Major point 4.
The authors did not directly address the previous major comment 3, and the model still does not describe different modes of action for every MEK inhibitor (MEKi).
According to the estimated parameters (https://github.com/labsyspharm/marm2-supplement/blob/main/MARM/parameters/RTKERK_pRAF_EGF_EGFR_MEKi_PRAFi_RAFi.csv), the MEKi-specific parameters are followings:

- bind_MEKi_MEK_dG (ΔG in energy rule, equivalent to Kd)
- bind_MEKi_MEK_kf (binding rate for MEK-MEKi)
- catalyze_RAFrafiNone_MEKi_p_kcatr (scaling factor for the phosphorylation rate in BRAFV600E reaction channel when substrate MEK is bound to MEKi)
- ep_pMEK_MEKi_ddG (difference in the affinity between MEK-MEKi and pMEK-MEKi)

Thus, the authors do not consider whether MEKi induces or disrupts the RAF-MEK complex. The thermodynamics of RAF-MEK binding and the changes in the RAF-MEK Kd induced by allosteric MEKi binding effects must be included in the model, if the authors claim that their model correctly describes modes of action of different MEKi.

Reviewer #2:

The authors made quite some efford to improve the manuscript. Most importantly they included the ERK-RAF feedback. Yet, as also referee 1 noted, there is still a discrepancy between the very detailed nature of the model and the claims of beeing very quantitative on the one hand and lack of important details (see reviewer 1) on the other hand. (e.g. from the abstract: "Causal tracing demonstrates that this provides a quantitative explanation for initial and acquired responses to multiple different RAF and MEK inhibitors individually and in combination."). Of course a model cannot and should not cover every detail, but when a model covers details, they should be right (here I am with reviewer 1). For instance, in this model the feedback that causes "adaptive" resistance. This would not neccessarily need such a very detailed model, and groups reported this previously with much simpler models (only in MSB: earliest: doi: 10.1038/msb.2013.29. latest: doi: 10.15252/msb.202110670).

I see the value of the paper mainly in its technical contributions (which are the strength of the paper and which are important).

Reviewer #3:

The authors sufficiently answered the extensive reviewer comments, and the paper is fit to publish.

**Detailed Response to Review**
**MSB-2022-10988**
Fröhlich, Sorger et al *Mechanistic model of MAPK signaling reveals how allostery and rewiring contribute to drug resistance*

The point-by-point response review below reviewer's comments are in boxes with yellow shading; our responses are in boxes with no shading.

## Editorial Comments

**Editor:**

Thank you for sending us your revised manuscript. First of all, please accept my apologies for the slow process, which was due to the late arrival of reviewer reports. We have now heard back from the three reviewers who agreed to evaluate your study. From the comments below, you will see that Reviewer #3 was satisfied with the revisions. The other two reviewers still raised a number of issues, while Reviewer #2 is overall more supportive. Given the balance of these evaluations, we feel that we can consider a revision of your manuscript if you can address the following issues that have been raised.

**Response:**
We thank the editor for this evaluation and have revised the text of the manuscript based on the editor's recommendations as described below. We believe that we have successfully responded to all of the outstanding issues.

**Editor:**
- Both Reviewers #1 and #2 raised concerns about the model and were not convinced that the model generates a precise and quantitative description of MAPK signaling. Therefore, we think it is essential to discuss the limitation of the model (in light of Reviewer #1's comments) and tone down the claim about the model being quantitative.

**Response:**
By quantitative we meant that we have described MAPK signaling with a calibrated mathematical models that yields precise predictions that can be compared to experimental data. This contrasts with a descriptive word model. In this commons sense of "quantitative" we believe that we have met the commonly accepted standard.

Nonetheless, to address this concern, have revised the paper so that it no longer make any statement about having a quantitative model. We have also revised the description of the model formulation as well as the description of the model limitations based on feedback by reviewer 1.

**Text Changes:** Abstract, page 15 paragraph 1, page 31 paragraph 2, page 32 paragraph 1

**Editor:**
- Considering the technical advance of the study, which Reviewer #2 thought was the strength and value of the manuscript, we would ask you to revise the manuscript to focus more on the technical aspect.

**Response:**
We thank the editor for this suggestion and have rephrased parts of the introduction and discussion accordingly.

**Text Changes:** Abstract, page 6 paragraph 2, page 30 paragraph 2/3, page 31 paragraph 1

## Reviewer #1

**Reviewer 1:**
Fabian Froehlich and colleagues put considerable effort into the manuscript revision. They clarified key definitions and incorporated the missing inhibitory RAF phosphorylation by ERK in the model. Although I think that the paper can be eventually published, my main previous concerns were not addressed. The authors' use of 'creative' language, such as 'causal decomposition', and a concept of two reaction channels creates the illusion of understanding but does little to deepen biological insights. This language also accomplishes little to convince biologists to see how dynamic model predictions can help them in their efforts to understand why the ERK signaling in cells is genetically resistant to RAF/MEK inhibitors or rapidly acquires resistance.

**Response:**
We thank the reviewer for noticing the extensive changes we met in the last revision of the manuscript. The reviewer will notice that our addition of a "missing" but ultimately non-identifiable inhibitory RAF phosphorylation, a major concern in the last round of review, did not substantially change any of our conclusions or predictions. Thus, we feel that changes the reviewer is arguing primarily concern the stylistic feature of quantitative models. Style is important of course, and we apologize that we have been unable to adjust our style to match the reviewer's preferences.

In particular we remain convinced that the concept of a reaction channel is generally helpful we attempt to describe multiple simultaneous processes that occur in cells involving may of the same components. We have once again substantially revised the section of the manuscript that introduces the reaction channels concept as well as its conclusions in the discussion. We hope that this, together with the more detailed changes described below addresses the reviewers concerns.

**Text Changes:** Page 19 paragraph 2, page 20 paragraph 1, page 30 paragraph 2

### Major points

**Reviewer 1 (Major Point 1):**
In the revised manuscript, the authors properly clarified their definition of "two reaction channels". However, this concept in my opinion does not really help us understanding the mechanism of the ERK pathway signaling and the development of drug resistance.
Lines 369-383 of the main text and the author's code define a 'RAS reaction channel' that involves RAF dimers bound to RAS. A 'BRAFV600E reaction channel' involves BRAFV600E monomers and dimers that are not bound to RAS or dimers with only one molecule RAF bound to RAS. However, even if the RAF dimer is not bound to RAS, it is still resistant to αC-OUT, DFG-IN RAF inhibitors (RAFi). Thus, the BRAFV600E reaction channel is also partially resistant to αC-OUT, DFG-IN RAFi. Moreover, the part of the BRAFV600E channel that consists of free dimers unbound to RAS changes depending on the RAFi. Almost all RAFi will increase the amount of such dimers. In the case of p61 BRAFV600E splice variants such dimers will be dominant, making the BRAFV600E reaction channel completely resistant to αC-OUT, DFG-IN RAF inhibitors.
Thus, the concept of reaction channels fails to clearly distinguish the molecular complexes resistant to αC-OUT, DFG-IN RAF inhibitors and the molecular complexes sensitive to αC-OUT, DFG-IN RAF inhibitors. Moreover, this formal definition of reaction channels does not cover the known resistance mechanisms and also differs from the accepted concept of RAF monomers and dimers. Therefore, the usefulness of this concept is questionable.

**Response:**
The reaction channels in this paper were not formulated around resistance mechanisms but around modes MAPK of activation – the BRAF oncogene in one case and RTKs in the other. Given the diversity of mechanisms used but drugs targeting MAPK components we agree with the reviewer that two channels are unlikely to be sufficient to fully capture how RAF/RAS signaling responds to inhibition. In particular, the concerns raised about the extent to which RAF dimers unbound or incompletely bound to RAS can be assigned to a specific reaction channel is valid. However, we note that it would be similarly confusing to include these RAF dimers in the RAS reaction channel, as any kind of (RAS-independent) RAF dimerization would then appear to inhibit BRAFV600E signaling.

When preparing this most recent set of revisions, we considered the possibility of adding a third reaction channel as a means to resolve ambiguity in functions of various RAS complexes. However, both the analysis described in manuscript as well as additional simulation experiments we performed showed that the RAF dimer species of concern to the reviewer do not appreciably contribute to MEK and ERK signaling. We decided that introducing a third channel that does not really contribute to overall signaling dynamics would increase complexity and confuse readers without deepening insight. However, the possibility that additional reaction channels could be added to add detail to a model suggests that the concerns raised by the reviewer are not shortcomings of the reaction channel concept itself, but rather of our "stylistic" choice of sticking with a simpler formulation.

To address the reviewer's concerns, we have once again substantially revised the section of the manuscript that introduces the reaction channels concept as well as its conclusions in the discussion.

**Text Changes:** Page 19 paragraph 2, page 20 paragraph 1, page 30 paragraph 2

**Reviewer 1 (Major Point 2):**
It was previously shown that both RAF monomers and dimers have non-zero kinase activity [doi:10.1128/MCB.26.6.2262-2272.2006]. It was also shown that BRAF-CRAF dimers have higher kinase activity than BRAF-BRAF and CRAF-CRAF dimers [doi:10.1128/MCB.26.6.2262-2272.2006]. Neither of these facts is incorporated into the present model. RAF monomers, except BRAFV600E, have no kinase activity in the model. In the model, the kinase activity of BRAFV600E monomers and all RAF dimers depends on the so-called channel (that is on whether the RAF dimer is bound to RAS-GTP) whereas it also critically depends on the composition of a RAF dimer, as mentioned above.

**Response:**
We agree with the reviewer that these are known mechanisms that have not been incorporated in the model, but we have no evidence that these mechanisms are relevant for the observations presented in this study, nor that respective rate parameters could be inferred from the available data. As stated by reviewer 2, we have chosen to limit the extent to which we describe mechanisms that remain non-identifiable in the model.

We also want to note that the kinase activity of BRAFV600E and RAF dimers does not depend on the reaction channel, but that the reaction channels recapitulate the causal structure of the underlying model.

To address the reviewer's concerns, we have introduced an additional section to the discussion that examines the limitations of the model formulation.

**Text Changes:** page 30 paragraph 2/3, page 31 paragraph 1.

**Reviewer 1 (Major Point 3):**
The authors do not consider dimer asymmetry in the model, having only one factor g, instead of introducing three factors, g_i, for an asymmetric dimer - inhibitor interactions This shortcoming can lead to incorrect descriptions of RAFi binding and RAF dimerization reactions in the model.
For example, in the Figure EV5D (in the updated manuscript the authors refer to Figure EV5E in line 645, but I think this is EV5D), the authors attempted to predict the effect of combining three drugs: LY3009120, Cobimetinib, and Vemurafenib. When incorporating structurally different RAF inhibitors to the model (in this case type II and type I1/2), we need at least four independent thermodynamic factors for each RAFi. In the model, the authors implement the allosteric effect caused by RAFi as follows (cell 15 in Model

Documentation.ipynb, RAF inhibition section):
## Single RAFi bound Raf dimers
EnergyPattern('ep_BRAF_BRAF_mod_RAFi_single', BRAF(raf=1) % BRAF(raf=1, rafi=2) % RAFi(raf=2), ep_RAF_RAF_mod_RAFi_single_ddG)
EnergyPattern('ep_BRAF_CRAF_mod_RAFi_single', BRAF(raf=1) % CRAF(raf=1, rafi=2) % RAFi(raf=2), ep_RAF_RAF_mod_RAFi_single_ddG)
EnergyPattern('ep_CRAF_BRAF_mod_RAFi_single', CRAF(raf=1) % BRAF(raf=1, rafi=2) % RAFi(raf=2), ep_RAF_RAF_mod_RAFi_single_ddG)
EnergyPattern('ep_CRAF_CRAF_mod_RAFi_single', CRAF(raf=1) % CRAF(raf=1, rafi=2) % RAFi(raf=2), ep_RAF_RAF_mod_RAFi_single_ddG)

## Double RAFi bound Raf dimers
EnergyPattern('ep_BRAF_BRAF_mod_RAFi_double', RAFi(raf=2) % BRAF(raf=1, rafi=2) % BRAF(raf=1, rafi=3) % RAFi(raf=3), ep_RAF_RAF_mod_RAFi_double_Gf)
EnergyPattern('ep_BRAF_CRAF_mod_RAFi_double', RAFi(raf=2) % BRAF(raf=1, rafi=2) % CRAF(raf=1, rafi=3) % RAFi(raf=3), ep_RAF_RAF_mod_RAFi_double_Gf)
EnergyPattern('ep_CRAF_CRAF_mod_RAFi_double', RAFi(raf=2) % CRAF(raf=1, rafi=2) % CRAF(raf=1, rafi=3) % RAFi(raf=3), ep_RAF_RAF_mod_RAFi_double_Gf);

In the supplemental Jupyter Notebook the authors wrote:
> In this implementation, C-Raf and B-Raf both correspond to kinase monomers R of the Kholodenko Core Model (KCM). K1 of the KCM is equivalent to the parameter bind_RAF_RAF_dG, which was introduced in the previous Section. K2 in the KCM is equivalent to bind_RAFi_RAF_dG, f to ep_RAF_RAF_mod_RAFi_single_ddG and g to ep_RAF_RAF_mod_RAFi_double_ddG.

Here the number of independent thermodynamic factors is two: ep_RAF_RAF_mod_RAFi_single_ddG and ep_RAF_RAF_mod_RAFi_double_ddG.

Therefore, the model presented in this study cannot quantitatively describe the effect of combination treatment with two structurally different RAFi and MEKi. This effect is not negligible because a combination of type II RAFi and a type I1/2 RAFi is highly synergistic (doi: 10.1016/j.cels.2018.06.002). Even if the association/dissociation rates of the RAFi-RAF complex is fitted to be RAFi-specific via parameter estimation, the model does not describe the change in the affinity of second RAFi binding caused by the first structurally-different RAFi binding. This can lead to wrong conclusions about inhibitor synergy/additivity/antagonism. Thus, the text and figure related to triple combination predictions must be deleted, or the model should be amended.

**Response:**
We that the reviewer for bringing up this important point. While we were certainly aware of the work by Kholodenko, we failed to mention that respective asymmetric terms were not included in the model.

However, the primary purpose of Figure EV5D was to show that the model as currently constituted is **not** able to predict the data and will require extension. Thus, we are effectively confirming the reviewer's hypothesis that there are relevant mechanisms still missing from the model. However, we think it unlikely that this can be attributed to the asymmetric thermodynamic terms mentioned by the reviewer – particularly since the "missing synergy" we observe lies in the MEKi/panRAFi plane, not the RAFi/panRAFi plane.

To address the reviewer's concern, we have now included the missing asymmetric in the list of molecular mechanisms not included in the model. The figure reference was indeed to a wrong, which we have corrected.

**Text Changes: P**age 31 paragraph 1

**Reviewer 1 (Major Point 4):**
The authors did not directly address the previous major comment 3, and the model still does not describe different modes of action for every MEK inhibitor (MEKi).
According to the estimated parameters ([https://github.com/labsyspharm/marm2-supplement/blob/main/MARM/parameters/RTKERK_pRAF_EGF_EGFR_MEKi_PRAFi_RAFi.csv](https://github.com/labsyspharm/marm2-supplement/blob/main/MARM/parameters/RTKERK_pRAF_EGF_EGFR_MEKi_PRAFi_RAFi.csv)), the MEKi-specific parameters are followings:

- bind_MEKi_MEK_dG (ΔG in energy rule, equivalent to Kd)
- bind_MEKi_MEK_kf (binding rate for MEK-MEKi)

- catalyze_RAFrafiNone_MEKi_p_kcatr (scaling factor for the phosphorylation rate in BRAFV600E reaction channel when substrate MEK is bound to MEKi)
- ep_pMEK_MEKi_ddG (difference in the affinity between MEK-MEKi and pMEK-MEKi)

Thus, the authors do not consider whether MEKi induces or disrupts the RAF-MEK complex. The thermodynamics of RAF-MEK binding and the changes in the RAF-MEK Kd induced by allosteric MEKi binding effects must be included in the model, if the authors claim that their model correctly describes modes of action of different MEKi.

**Response:**
We thank the reviewer for this comment, as we have to appear to have misunderstood his comments. In our understanding, major point 3 in the previous round of revisions referred to the concern that MEK inhibitors have to be modeled differently across different inhibitors, which has been the case in all versions of the model and the manuscript. We now see that the reviewer is also unhappy with the way in which we modeled specific interactions, which we will now comment on below.

In contrast to RAFi, the exact mode of action of MEKis is far less well understood and potentially substantially more involved. To the best of our knowledge, there are at least five different hypothetical mechanisms that contribute to type III MEKi mode of action, including (i) allosteric modulation of RAF-MEK affinity, (ii) allosteric modulation of RAF-pMEK affinity, (iii) difference in uMEK vs pMEK affinity, (iv) inhibition of MEK phosphorylation and (v) inhibition of ERK phosphorylation. While all these mechanisms have been experimentally validated with purified components, the extent to which they actually influence phosphorylation dynamics in a cell is not really understood. This is because the apparent effects of many of these mechanisms are indistinguishable when experimentally quantified. For example, RAF-MEK complex assembly can be quantified using co-immunoprecipitation experiments (as done in, e.g., doi:10.1158/2159-8290.CD-20-1351 or doi:10.1016/j.ccr.2014.07.007), but any MEKi dependent changes in RAF-MEK complex assembly could be the direct result of (a) allosteric modulation of the RAF-MEK complex assembly itself (i.e., mechanism (i)) or the indirect result of (b) allosteric modulation of product (pMEK) release (i.e., mechanism (ii)), (c) modulation of MEK phosphorylation rates and resulting changes in product (pMEK) release (i.e., mechanism (iv)) or (d) disruption of negative phosphorylation feedback on RAF mediated by pERK and the resulting changes in MEK/RAF affinity, (i.e., mechanism (v)). This means that a measured changes in apparent RAF-MEK affinity is not *ipso facto* evidence of allosteric modulation of the RAF-MEK complex assembly. Moreover, and more importantly, it means that the disruption of RAF-MEK complexes is not necessarily the causal mechanism that determines the inhibition strength of MEKis. In fact, doi: 10.1073/pnas.2107207118, reports allosteric modulation of the RAF-MEK complex, but concludes “that allosteric MEKi do not act primarily by preventing binding of uMEK to RAF”. Therefore, we find it justified not to include this mechanism in the model. We have verified that the model does in indeed recapitulate the dissociation of RAF-MEK complexes upon MEKi addition despite not including the respective allosteric mechanism.

Mechanism (iii) was not included as product release is not explicitly modeled, but we find it reasonable to assume that the respective effect can be accounted for by the scaling factor for the MEKi bound phosphorylation rate.

We acknowledge that these decisions we took during model formulation have not been sufficiently motivated in the manuscript and, to address the reviewer’s concerns, we have now extended respective passages in the paper.

**Text Changes:** Page 15 paragraph 1

## Reviewer #2

**Reviewer 2:**
The authors made quite some effort to improve the manuscript. Most importantly they included the ERK-RAF feedback. Yet, as also referee 1 noted, there is still a discrepancy between the very detailed nature of the model and the claims of being very quantitative on the one hand and lack of important details (see reviewer 1) on the other hand. (e.g. from the abstract: "Causal tracing demonstrates that this provides a quantitative explanation for initial and acquired responses to multiple different RAF and MEK inhibitors individually and in combination."). Of course a model cannot and should not cover every detail, but when a model covers details, they should be right (here I am with reviewer 1). For instance, in this model the feedback that causes "adaptive" resistance. This would not necessarily need such a very detailed model, and groups reported this previously with much simpler models (only in MSB: earliest: doi: 10.1038/msb.2013.29. latest: doi: 10.15252/msb.202110670).

I see the value of the paper mainly in its technical contributions (which are the strength of the paper and which are important).

**Response:**
We thank the reviewer for these comments and appreciate his feedback. While we generally agree with the reviewer’s position, we find it difficult to assess at which point a detail that we put in the model is “right”. In fact, virtually any published systems biology model could be subjected to the same criticism, as model construction remains a subjective process. None of the concerns that reviewer 1 raises described a situation where we implemented a mechanism in the model that was not supported by literature, i.e., where the model would clearly be wrong. Instead, as far as we understand the criticisms, the issue is always that we omitted certain mechanisms that we deemed unimportant but that the reviewer deemed important. In this regard, we note that our reworking of the paper in the previous round of revision to ERK-RAF did not result in any significant changes in the results or otherwise change our conclusions. This is most likely because the mechanism is non-identifiable in the context of other feedback mechanisms operating on similar time scales.

We are forced to accept the somewhat idiosyncratic nature of the model formulation process because we currently lack systematic methods for coarse graining that allow us to account for mechanistic details without explicitly encoding them in model equations. Thus, even with the advances we made in this manuscript, we find ourselves unable to train a model that accounts for every last (relevant) detail because the model simply becomes too large and unwieldy. Thus, while it would certainly be feasible to include some more of the mechanisms that were mentioned by Reviewer 1, but would require a non-trivial amount of effort and compute time, without clear evidence that the model would be substantially more “quantitative” or that we would really gain any new insight.

Importantly, we want to stress that it is not our intention to claim that this model is an unbiased representation of everything that we know about EGFR and ERK signaling, but rather that it is a continuation of the work on structure-based modeling that was pioneered by Kholodenko, Faeder and colleagues. Accordingly, we appreciate the feedback that we should emphasize the technical contributions of the paper. We have carefully revised the paper again, toning down the language in some places as well as rewriting some parts of the abstract, introduction and discussion (see point by point response to editor).

Moreover, we have addressed the concerns about the model raised by reviewer 1 through textual changes (see point by point response to reviewer).

## Reviewer #3:

**Reviewer 3:**
The authors sufficiently answered the extensive reviewer comments, and the paper is fit to publish.

**Response:**
We thank the reviewer for this positive evaluation.

## 2nd Revision - Editorial Decision

**15th Dec 2022**

Thank you again for sending us your revised manuscript. We are now satisfied with the modifications made and I am pleased to inform you that your paper has been accepted for publication.

## EMBO Press Author Checklist

| | |
|---|---|
| Corresponding Author Name: Peter Sorger | |
| Journal Submitted to: Molecular Systems Biology | |
| Manuscript Number: MSB-2022-10988 | |

**USEFUL LINKS FOR COMPLETING THIS FORM**
The EMBO Journal - Author Guidelines
EMBO Reports - Author Guidelines
Molecular Systems Biology - Author Guidelines
EMBO Molecular Medicine - Author Guidelines

**Reporting Checklist for Life Science Articles (updated January**
This checklist is adapted from Materials Design Analysis Reporting (MDAR) Checklist for Authors. MDAR establishes a minimum set of requirements in transparent reporting in the life sciences (see Statement of Task: 10.31222/osf.io/9sm4x). Please follow the journal's guidelines in preparing your
**Please note that a copy of this checklist will be published alongside your article.**

**Abridged guidelines for figures**
**1. Data**
The data shown in figures should satisfy the following conditions:
- the data were obtained and processed according to the field's best practice and are presented to reflect the results of the experiments in an accurate and unbiased manner.
- ideally, figure panels should include only measurements that are directly comparable to each other and obtained with the same assay.
- plots include clearly labeled error bars for independent experiments and sample sizes. Unless justified, error bars should not be shown for technical
- if n<5, the individual data points from each experiment should be plotted. Any statistical test employed should be justified.
- Source Data should be included to report the data underlying figures according to the guidelines set out in the authorship guidelines on Data

**2. Captions**
Each figure caption should contain the following information, for each panel where they are relevant:
- a specification of the experimental system investigated (eg cell line, species name).
- the assay(s) and method(s) used to carry out the reported observations and measurements.
- an explicit mention of the biological and chemical entity(ies) that are being measured.
- an explicit mention of the biological and chemical entity(ies) that are altered/varied/perturbed in a controlled manner.
- the exact sample size (n) for each experimental group/condition, given as a number, not a range;
- a description of the sample collection allowing the reader to understand whether the samples represent technical or biological replicates (including how many animals, litters, cultures, etc.).
- a statement of how many times the experiment shown was independently replicated in the laboratory.
- definitions of statistical methods and measures:
  - common tests, such as t-test (please specify whether paired vs. unpaired), simple χ2 tests, Wilcoxon and Mann-Whitney tests, can be unambiguously identified by name only, but more complex techniques should be described in the methods section;
  - are tests one-sided or two-sided?
  - are there adjustments for multiple comparisons?
  - exact statistical test results, e.g., P values = x but not P values < x;
  - definition of 'center values' as median or average;
  - definition of error bars as s.d. or s.e.m.

**Please complete ALL of the questions below.**
**Select "Not Applicable" only when the requested information is not relevant for your study.**

**Materials**

| Newly Created Materials | Information included in the manuscript? | In which section is the information available? (Reagents and Tools Table, Materials and Methods, Figures, Data Availability Section) |
|---|---|---|
| New materials and reagents need to be available; do any restrictions apply? | Not Applicable | |

| Antibodies | Information included in the manuscript? | In which section is the information available? (Reagents and Tools Table, Materials and Methods, Figures, Data Availability Section) |
|---|---|---|
| For **antibodies** provide the following information:<br>- Commercial antibodies: RRID (if possible) or supplier name, catalogue number and or/clone number<br>- Non-commercial: RRID or citation | Yes | Materials and Methods |

| DNA and RNA sequences | Information included in the manuscript? | In which section is the information available? (Reagents and Tools Table, Materials and Methods, Figures, Data Availability Section) |
|---|---|---|
| **Short novel DNA or RNA including primers, probes**: provide the sequences. | Not Applicable | |

| Cell materials | Information included in the manuscript? | In which section is the information available? (Reagents and Tools Table, Materials and Methods, Figures, Data Availability Section) |
|---|---|---|
| **Cell lines:** Provide species information, strain. Provide accession number in repository **OR** supplier name, catalog number, clone number, and/**OR** RRID. | Yes | Material and Methods |
| **Primary cultures:** Provide species, strain, sex of origin, genetic modification status. | Not Applicable | |
| Report if the cell lines were recently **authenticated** (e.g., by STR profiling) and tested for mycoplasma contamination. | Yes | Material and Methods |

| Experimental animals | Information included in the manuscript? | In which section is the information available? (Reagents and Tools Table, Materials and Methods, Figures, Data Availability Section) |
|---|---|---|
| **Laboratory animals or Model organisms:** Provide species, strain, sex, age, genetic modification status. Provide accession number in repository **OR** supplier name, catalog number, clone number, **OR** RRID. | Not Applicable | |
| **Animal observed in or captured from the field:** Provide species, sex, and age where possible. | Not Applicable | |
| Please detail **housing and husbandry conditions**. | Not Applicable | |

| Plants and microbes | Information included in the manuscript? | In which section is the information available? (Reagents and Tools Table, Materials and Methods, Figures, Data Availability Section) |
|---|---|---|
| **Plants:** provide species and strain, ecotype and cultivar where relevant, unique accession number if available, and source (including location for collected wild specimens). | Not Applicable | |
| **Microbes:** provide species and strain, unique accession number if available, and source. | Not Applicable | |

| Human research participants | Information included in the manuscript? | In which section is the information available? (Reagents and Tools Table, Materials and Methods, Figures, Data Availability Section) |
|---|---|---|
| If collected and within the bounds of privacy constraints report on age, sex and gender or ethnicity for all study participants. | Not Applicable | |

| Core facilities | Information included in the manuscript? | In which section is the information available? (Reagents and Tools Table, Materials and Methods, Figures, Data Availability Section) |
|---|---|---|
| If your work benefited from core facilities, was their service mentioned in the acknowledgments section? | Yes | Acknowledgements |

**Design**

| Study protocol | Information included in the manuscript? | In which section is the information available? (Reagents and Tools Table, Materials and Methods, Figures, Data Availability Section) |
|---|---|---|
| If study protocol has been **pre-registered, provide DOI in the manuscript**. For clinical trials, provide the trial registration number **OR** cite DOI. | Not Applicable | |
| Report the **clinical trial registration number** (at ClinicalTrials.gov or equivalent), where applicable. | Not Applicable | |

| Laboratory protocol | Information included in the manuscript? | In which section is the information available? (Reagents and Tools Table, Materials and Methods, Figures, Data Availability Section) |
|---|---|---|
| Provide DOI OR other citation details if **external detailed step-by-step protocols** are available. | Not Applicable | |

| Experimental study design and statistics | Information included in the manuscript? | In which section is the information available? (Reagents and Tools Table, Materials and Methods, Figures, Data Availability Section) |
|---|---|---|
| Include a statement about **sample size** estimate even if no statistical methods were used. | Not Applicable | |
| Were any steps taken to minimize the effects of subjective bias when allocating animals/samples to treatment (e.g. **randomization procedure**)? If yes, have they been described? | Not Applicable | |
| Include a statement about **blinding** even if no blinding was done. | Not Applicable | |
| Describe **inclusion/exclusion criteria** if samples or animals were excluded from the analysis. Were the criteria pre-established?<br><br>If sample or data points were omitted from analysis, report if this was due to attrition or intentional exclusion and provide justification. | Not Applicable | |
| For every figure, are **statistical tests** justified as appropriate? Do the data meet the assumptions of the tests (e.g., normal distribution)? Describe any methods used to assess it. Is there an estimate of variation within each group of data? Is the variance similar between the groups that are being statistically compared? | Not Applicable | |

| Sample definition and in-laboratory replication | Information included in the manuscript? | In which section is the information available? (Reagents and Tools Table, Materials and Methods, Figures, Data Availability Section) |
|---|---|---|
| In the figure legends: state number of times the experiment was **replicated** in laboratory. | Yes | |
| In the figure legends: define whether data describe **technical or biological replicates**. | Yes | |

## Ethics

| Ethics | Information included in the manuscript? | In which section is the information available? (Reagents and Tools Table, Materials and Methods, Figures, Data Availability Section) |
|---|---|---|
| Studies involving **human participants**: State details of **authority granting ethics approval** (IRB or equivalent committee(s), provide reference number for approval. | Not Applicable | |
| Studies involving **human participants**: Include a statement confirming that **informed consent** was obtained from all subjects and that the experiments conformed to the principles set out in the WMA Declaration of Helsinki and the Department of Health and Human Services Belmont Report. | Not Applicable | |
| Studies involving **human participants:** For publication of **patient photos**, include a statement confirming that consent to publish was obtained. | Not Applicable | |
| Studies involving experimental **animals**: State details of **authority granting ethics approva**l (IRB or equivalent committee(s), provide reference number for approval. Include a statement of compliance with ethical regulations. | Not Applicable | |
| Studies involving **specimen and field samples:** State if relevant **permits** obtained, provide details of authority approving study; if none were required, explain why. | Not Applicable | |

| Dual Use Research of Concern (DURC) | Information included in the manuscript? | In which section is the information available? (Reagents and Tools Table, Materials and Methods, Figures, Data Availability Section) |
|---|---|---|
| Could your study fall under dual use research restrictions? Please check biosecurity documents and list of **select agents and toxins** (CDC): https://www.selectagents.gov/sat/list.htm | Not Applicable | |
| If you used a select agent, is the security level of the lab appropriate and reported in the manuscript? | Not Applicable | |
| If a study is subject to dual use research of concern regulations, is the name of the **authority granting approval and reference number** for the regulatory approval provided in the manuscript? | Not Applicable | |

## Reporting

The MDAR framework recommends adoption of discipline-specific guidelines, established and endorsed through community initiatives. Journals have their own policy about requiring specific guidelines and recommendations to complement MDAR.

| Adherence to community standards | Information included in the manuscript? | In which section is the information available? (Reagents and Tools Table, Materials and Methods, Figures, Data Availability Section) |
|---|---|---|
| State if relevant guidelines or checklists (e.g., **ICMJE, MIBBI, ARRIVE, PRISMA**) have been followed or provided. | Not Applicable | |
| For **tumor marker prognostic studies,** we recommend that you follow the **REMARK** reporting guidelines (see link list at top right). See author guidelines, under 'Reporting Guidelines'. Please confirm you have followed these guidelines. | Not Applicable | |
| For **phase II and III randomized controlled trials**, please refer to the **CONSORT** flow diagram (see link list at top right) and submit the CONSORT checklist (see link list at top right) with your submission. See author guidelines, under 'Reporting Guidelines'. Please confirm you have submitted this list. | Not Applicable | |

## Data Availability

| Data availability | Information included in the manuscript? | In which section is the information available? (Reagents and Tools Table, Materials and Methods, Figures, Data Availability Section) |
|---|---|---|
| Have **primary datasets** been deposited according to the journal's guidelines (see 'Data Deposition' section) and the respective accession numbers provided in the Data Availability Section? | Yes | Data Availability |
| Were **human clinical and genomic datasets** deposited in a public access-controlled repository in accordance to ethical obligations to the patients and to the applicable consent agreement? | Not Applicable | |
| Are **computational models** that are central and integral to a study available without restrictions in a machine-readable form? Were the relevant accession numbers or links provided? | Yes | Data Availability |
| If publicly available data were reused, provide the respective **data citations in the reference list**. | Not Applicable | |
